# 3-Cyanoallyl boronates are versatile building blocks in the synthesis of polysubstituted thiophenes†
†Electronic supplementary information (ESI) available. See DOI: 10.1039/c7sc00831g



**DOI:** 10.1039/c7sc00831g

**Published:** 2017-04-18

**Authors:** Wenjie Shao, Sherif J. Kaldas, Andrei K. Yudin

**Affiliations:** a Davenport Research Laboratories , Department of Chemistry , University of Toronto , 80 St. George St. , Toronto , ON M5S 3H6 , Canada . Email: ayudin@chem.utoronto.ca

## Abstract

We report the preparation of hitherto unprecedented 3-cyanoallyl boronates using condensation of the parent α-boryl aldehyde and nitriles. The resulting allyl boronates have been used to generate a wide range of borylated thiophenes, which represent a valuable class of heterocycles in modern drug discovery. Subsequent Suzuki–Miyaura cross-coupling enabled the synthesis of pharmaceutically important 3,5-disubstituted aminothiophenes. Moreover, late stage functionalization gave access to borylated bromothiophene and thieno[2,3-*b*]pyridines.

## Abstract 2

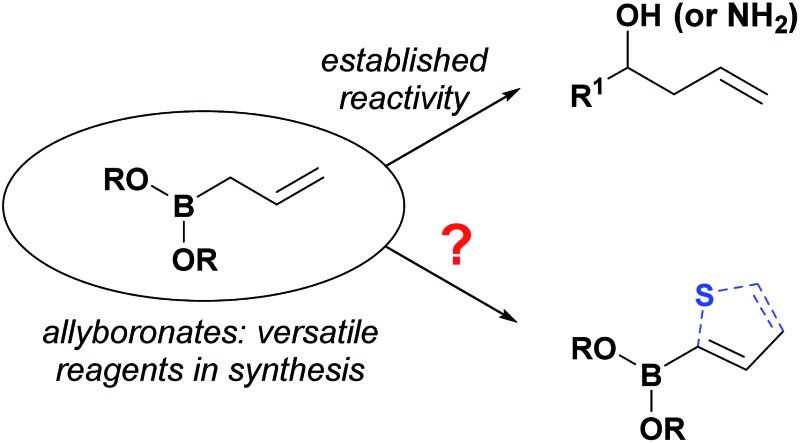
The Knoevenagel condensation between α-MIDA boryl acetaldehyde and active methylene compounds enables synthesis of novel electron-poor allylboronates and facilitates access to synthetically challenging thiophene derivatives.

## 

Allylboronates^1^ are among the most widely used building blocks in organic synthesis and are commonly employed in drug discovery. Despite the widespread application of allylboron reagents in chemical synthesis, their use has been largely limited to the corresponding allylation reactions, which typically utilize electron-rich allylboranes or boronates. In contrast, the preparation and application of electron-poor allylboronates has not received enough attention in organic synthesis to date. Most of the electron-withdrawing groups have been limited to the halogens^2^ or their position was restricted at C-2.^3^ We have come across a single example^4^ wherein a C-3 amide-containing allylboronate was isolated as a byproduct in 35% yield. Attempts were made to access the C-3 ester-containing electron-poor allylboronates, but both^5^ failed to give desired products. We wondered whether the C-3 substituted electron-poor allylboronates could be generally obtained from amphoteric α-(MIDA)boryl aldehydes,^6^ as MIDA-protected boron species showed enhanced stability^7^ that allow quick and facile access to previously inaccessible boron compounds.^6*f*^ If such allylboronates were accessible, the four-carbon unit arising from these allylboronate species could serve as the foundation in the synthesis of heterocycles (Scheme 1). To the best of our knowledge, heterocycle annulation from allylboronates has not received attention in synthesis. The boron motif found in the borylated heterocycle derivatives could enable late-stage cross-coupling with suitable partners or provide a handle for site-selective appendage of a boron-containing heterocycle using recently described protocols.^6*d*^

**Scheme 1:**
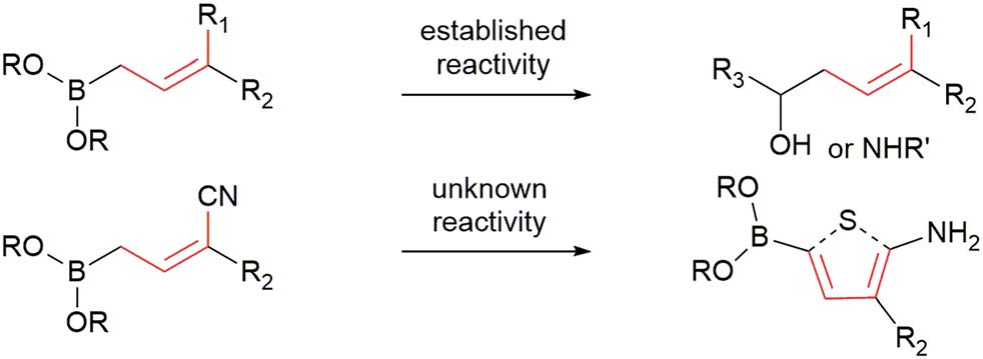
Contrasting typical reactivity of allyl boronates in allylation reactions with synthesis of heterocycles.

## Results and discussion

We commenced our study with the preparation of substituted 3-cyanoallyl boronates by reacting the α-boryl aldehyde^6*c*^ with nitrile derivatives. Malononitrile (**3a**) and ethyl cyanoacetate (**3b**) were first examined. We have determined that the highest yields were obtained when the Knoevenagel condensation was carried out in acetonitrile with diethylamine as the base (Table 1). Other organic bases, such as imidazole, triethylamine, morpholine, and piperidine gave low yields or no products. Under the optimized reaction conditions, the 3-cyanoallyl boronates were isolated in excellent yields (91% for **3a** and 93% for **3b**). Benzyl amide group was also tested and the reaction was clean, giving compound **3c** in 84% yield after isolation. Cyanoacetamide was well tolerated (**3d**), showing that the presence of the –NH_2_ group does not negatively affect the reaction outcome. For compounds **3b**, **3c** and **3d**, only one isomer was obtained from the reaction mixture and the geometry of the double bond was determined by NOESY experiments. However, the condensation reaction with cyanoacetic acid (R = COOH) resulted in complex mixture. The resulting allylboronates, bearing two electron-withdrawing groups at the terminus, have not been reported in the literature and would be difficult to access by other methods. With the successful preparation of these 3-cyanoallyl boronates, we pursued their application in the synthesis of polysubstituted borylated thiophenes.

**Table 1:** Synthesis of substituted 3-cyanoallyl boronates

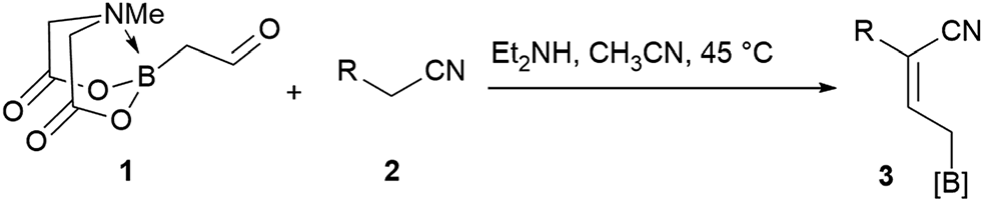
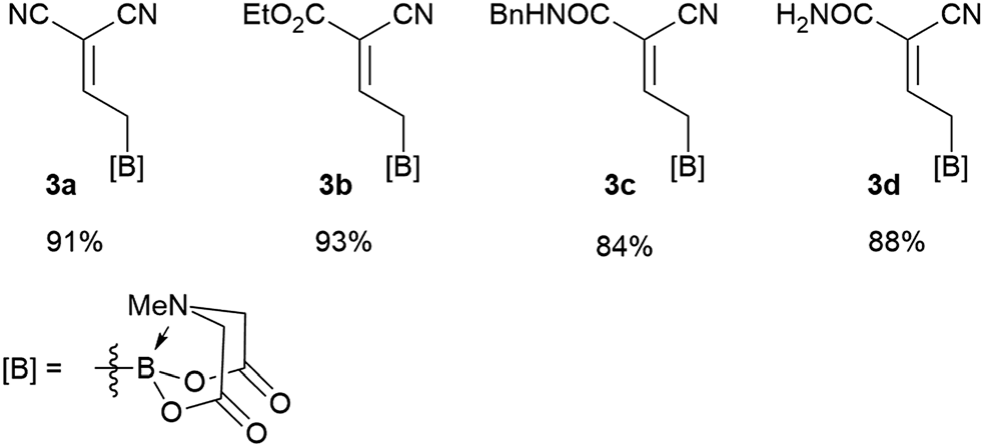

**Fig. 1:**
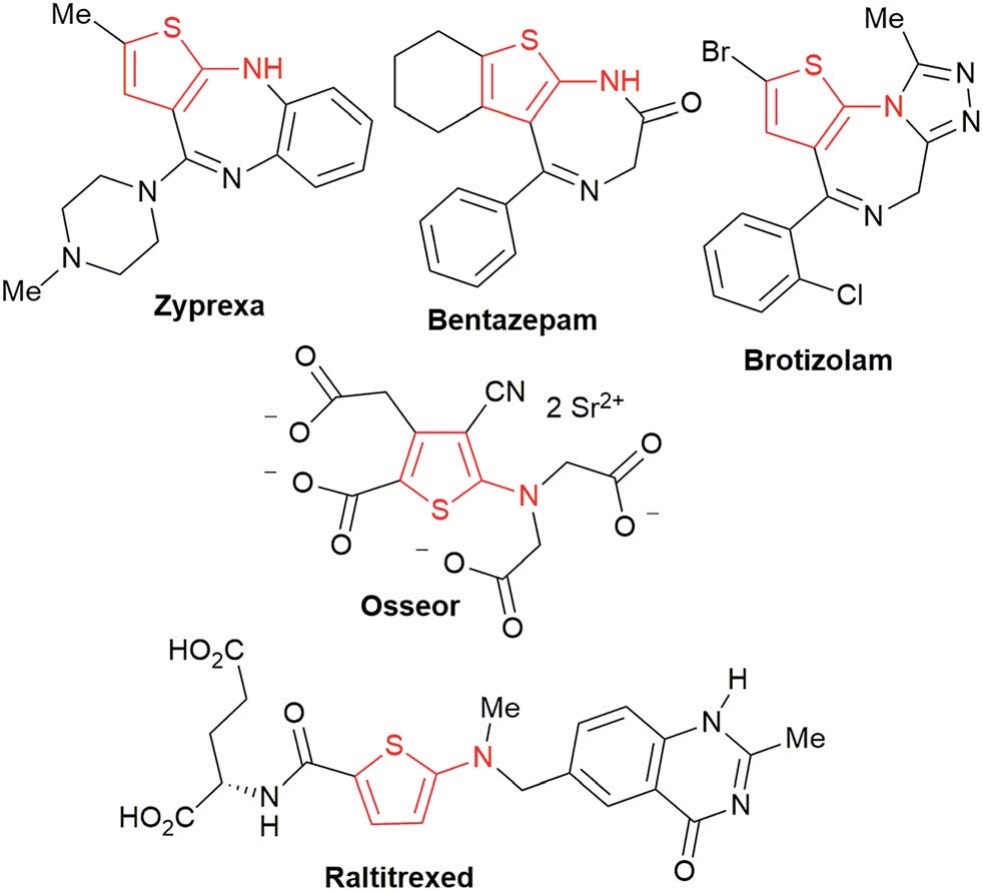
Examples of 2-aminothiophene-containing pharmaceutical drugs.

Polysubstituted thiophenes received attention as valuable building blocks in organic synthesis.^8^ They have been widely used in the pharmaceutical industry,^9^ dye chemistry,^10^ and as functional materials.^11^ In modern drug discovery, polysubstituted thiophenes are important because they constitute a bioisosteric replacement^12^ for the phenyl ring. Thiophene-containing derivatives are often characterized by reduced toxicity and better pharmacokinetic properties.^13^ Substituted 2-aminothiophenes are one of the most important thiophene categories^14^ as they are common structures in the FDA approved drugs (Fig. 1).

Depending on the functional groups tolerance and the stability of the products, borylated thiophenes are commonly synthesized through halogen–metal exchange^15^ on the preformed halothiophenes. Recent C–H activation^16^ with iridium catalysts showed considerable advantages, but is still restricted by the presence of directing groups and functional group tolerance. Borylation of aminothiophenes, on the other hand, is an appealing alternative to these methods that has received limited attention. This can partially be attributed to the protodeborylation reaction of the resulting products^16a,17^ as well as to the detrimental effect of the amino group. To the best of our knowledge, the reported examples only describe tertiary amine or amide-containing thiophenes.^18^ Therefore, a general method for making borylated aminothiophenes is highly desirable.

Our 3-cyanoallyl boron species could be readily converted to borylated thiophenes in the presence of elemental sulfur (the Gewald reaction). When treated with sulfur and Et_2_NH in THF, compounds **3a** and **3b** could be smoothly transformed to borylated thiophenes. We have also developed a more convenient one-pot process (Table 2). The reaction was best done at a relatively low concentration, no more than 0.05 M, to suppress the dimerization of the 3-cyanoallyl boronates intermediates.^19^ A variety of nitrile compounds were tested and the reactions were generally good with isolated yields varying from 33% to 91%. The electron density of the aromatic ring has a small effect on the reaction, as electron-rich phenyl (**4l**) gave lower yield (80%) while electron-poor (**4e**, **4f**) phenyl examples gave better yields (91% and 89%). Heteroaryl nitriles such as thiophene (**4g**), furane (**4i**), pyrrole (**4k**) were also suitable and some susceptible groups were well tolerated (**4d**, **4h**). It is noteworthy that all the products (**4a** to **4l**) are new and many of them cannot be easily made using alternative methods. Larger scale synthesis was also desirable. With that in mind, compound **4c** was obtained in 87% on a 0.5 g scale. The reaction was fast and TLC showed the complete consumption of the α-boryl aldehyde component in 3 hours. However, the sulfonyl example (**4j**) turned out to be more difficult to synthesize, requiring longer reaction time (24 h) and excess amount of (phenylsulfonyl)acetonitrile. The low yield of **4j** was attributed to the dimerization of the allylboronate intermediate. Most of the borylated aminothiophenes are partially water-soluble, thus aqueous work-up should be avoided to achieve highest yields. Additionally, we were pleased that no C–B bond scission was observed under the standard reaction conditions and the products are stable under ambient conditions.

**Table 2:** Synthesis of borylated thiophenes

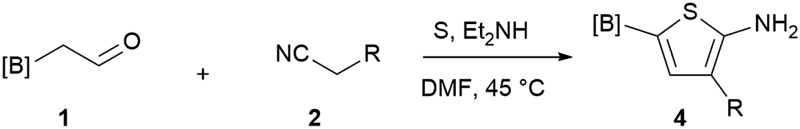			
Entry	Nitrile	Product	Yield
1	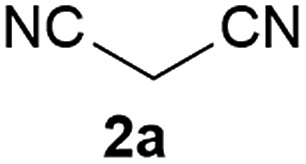	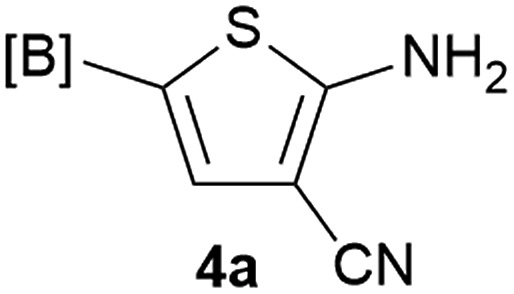	73%
2	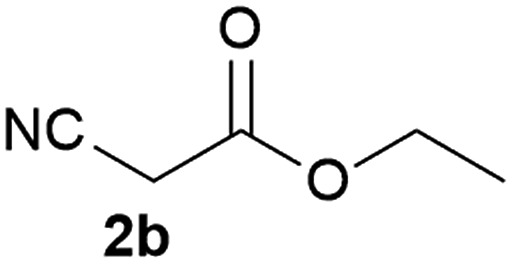	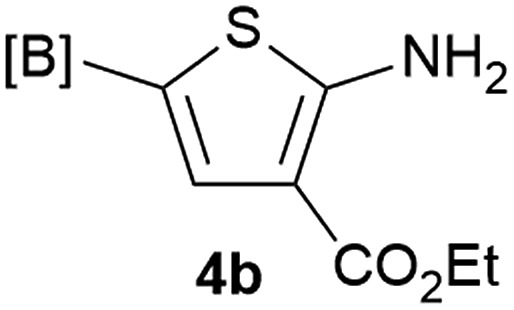	84%
3	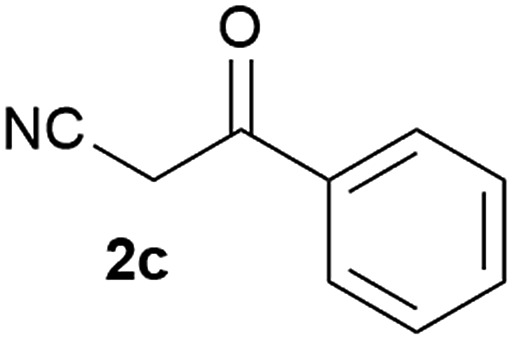	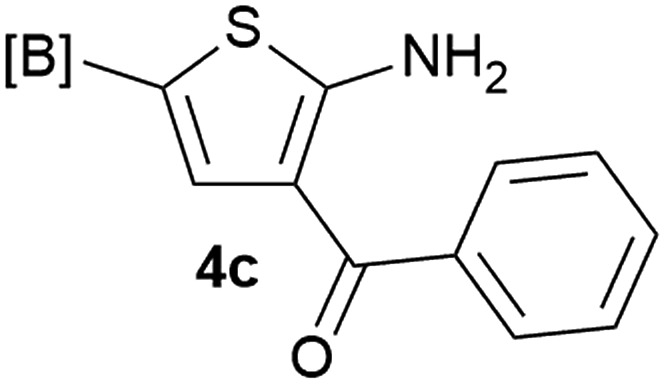	87%
4	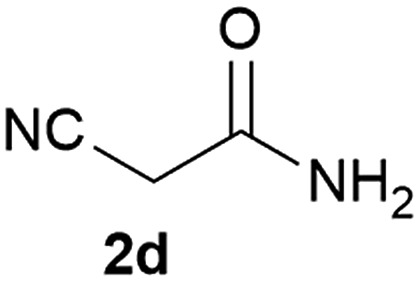	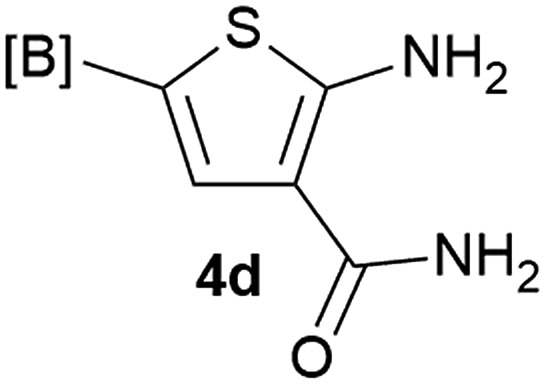	85%
5	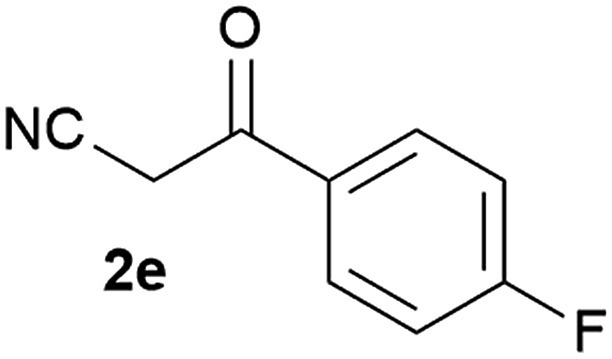	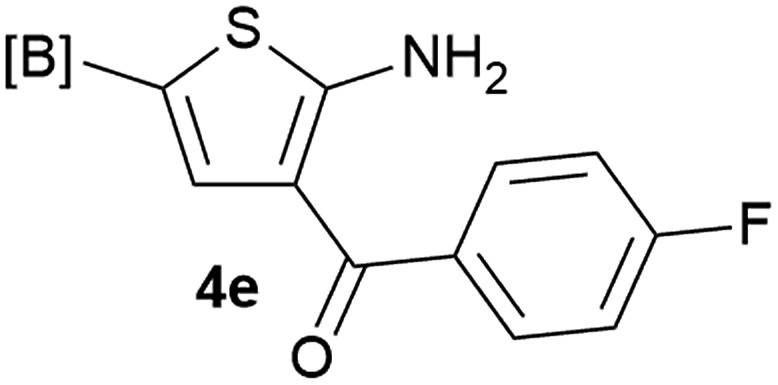	91%
6	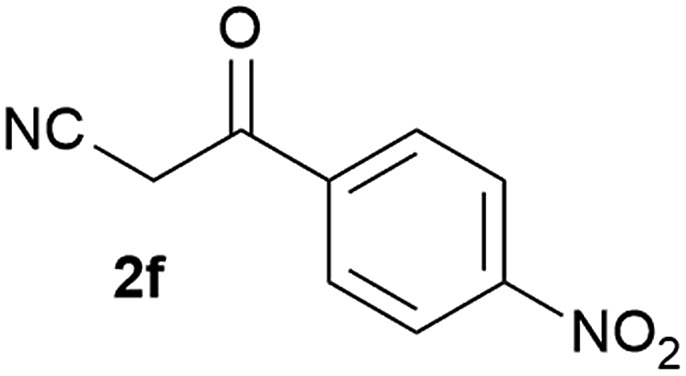	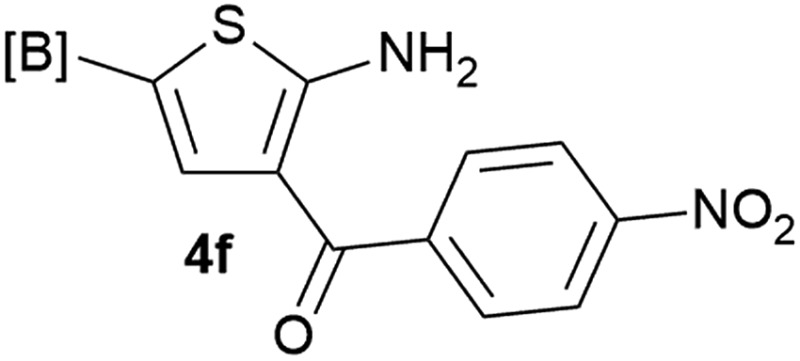	89%
7	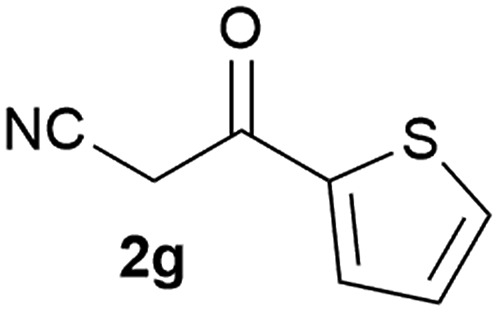	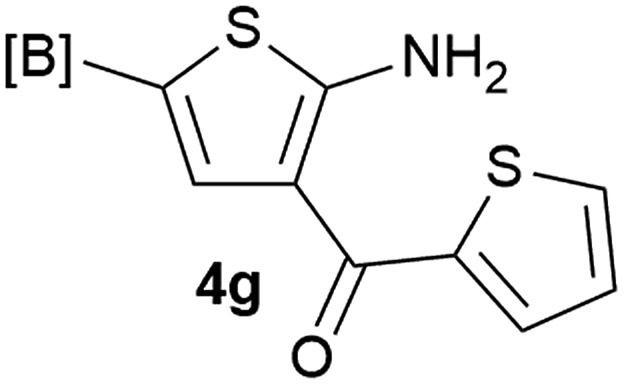	86%
8	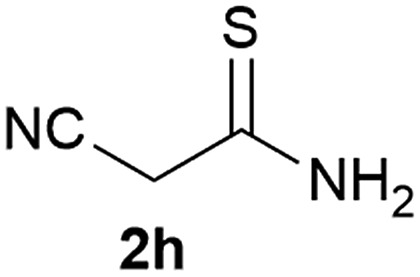	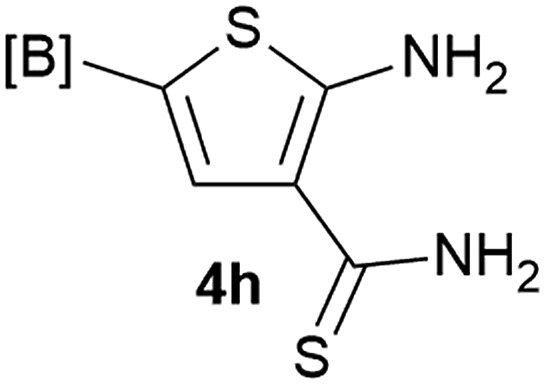	79%
9	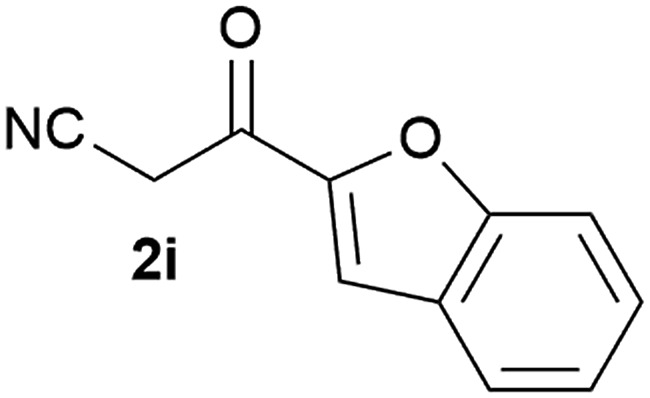	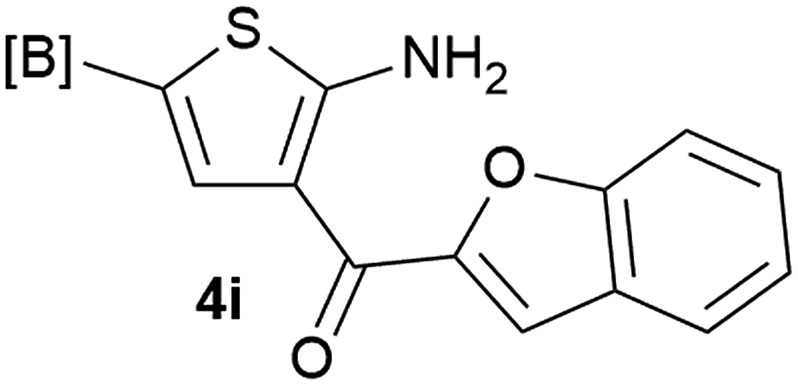	82%
10	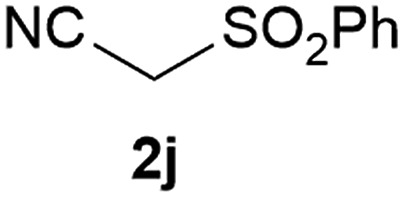	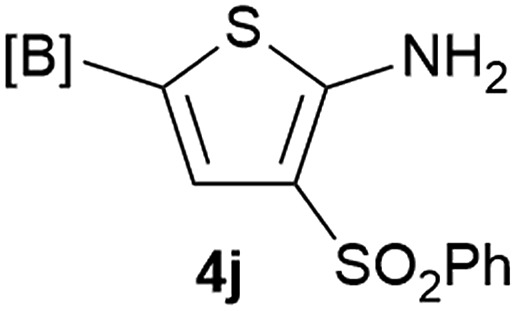	33%
11	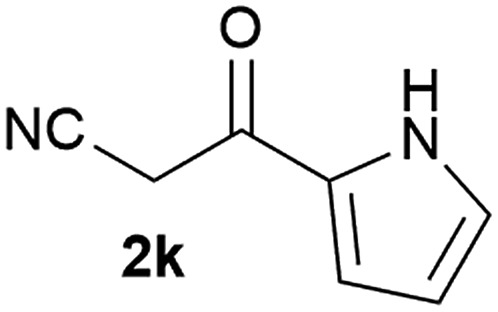	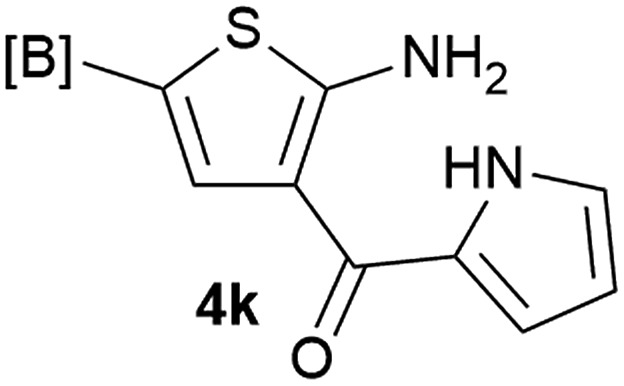	88%
12	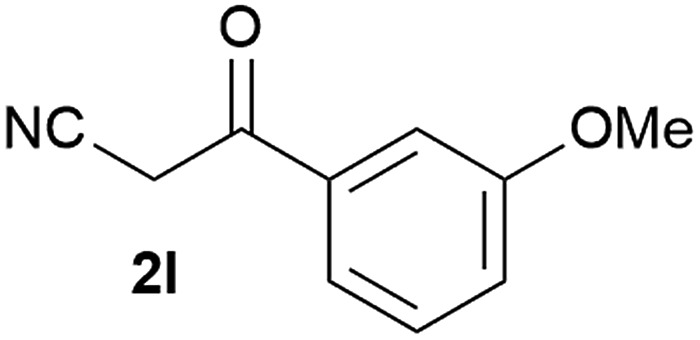	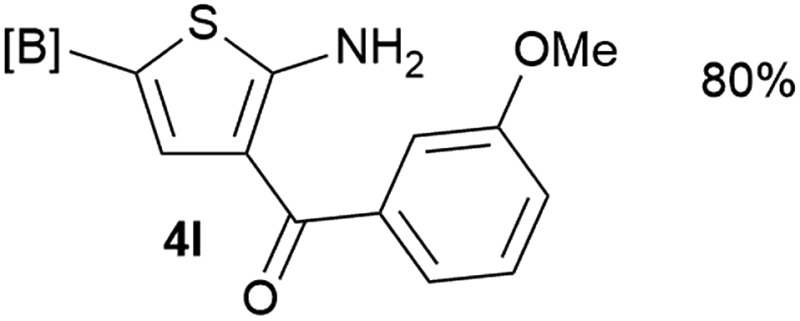	80%

**Scheme 2:**
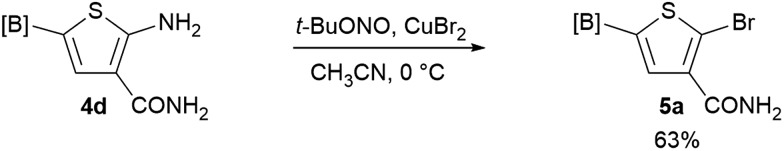
Synthesis of borylated bromothiophenes.

We sought to demonstrate our borylated aminothiophenes as intermediates to access other synthetically challenging borylated thiophene derivatives. A recent study showed that borylated bromothiophenes are promising monomers in material science due to their ability to polymerize under suitable Suzuki–Miyaura conditions.^20^ Our method offers a convenient route to previously inaccessible bromothiophene derivatives. Compound **4d** was reacted with *t*-BuONO and CuBr_2_ in acetonitrile to generate borylated bromothiophenes **5a** (Scheme 2), which was difficult to synthesize by alternative methods,^21^ in 63% yield.

It is known that 2-heteroaryl boronates are unfavorable substrates for Suzuki–Miyaura coupling due to the tendency of protodeborylation,^16*a*^ and the cross-coupling of borylated aminothiophenes could be even more challenging because of the interfering adjacent –NH_2_ group. To achieve reasonable yields of the cross-coupling reaction, an extensive screen of reaction conditions was carried out. For most of the screened conditions, mainly protodeborylation product was observed. Fortunately, it was found that with 0.1 equiv. RuPhos Pd G3 as the catalyst and 3.0 equiv. Na_2_CO_3_ as the base, the desired products **6a** and **6b** were obtained in 78% and 71% yields (Table 3). Though 3,5-disubstituted aminothiophenes can be generally prepared by Gewald reaction from suitable substituted acetaldehydes, our Suzuki approach gave access to 3,5-disubstituted variants, for which the corresponding acetaldehyde derivatives are not readily available (**6c**, **6d** and **6e**).^22^

**Table 3:** Suzuki–Miyaura-cross-coupling of aminothiophenes

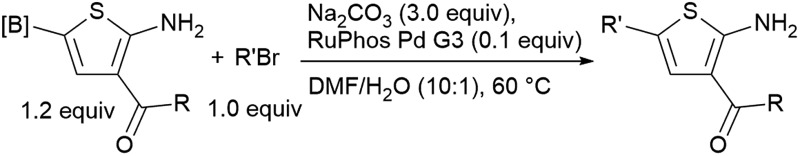
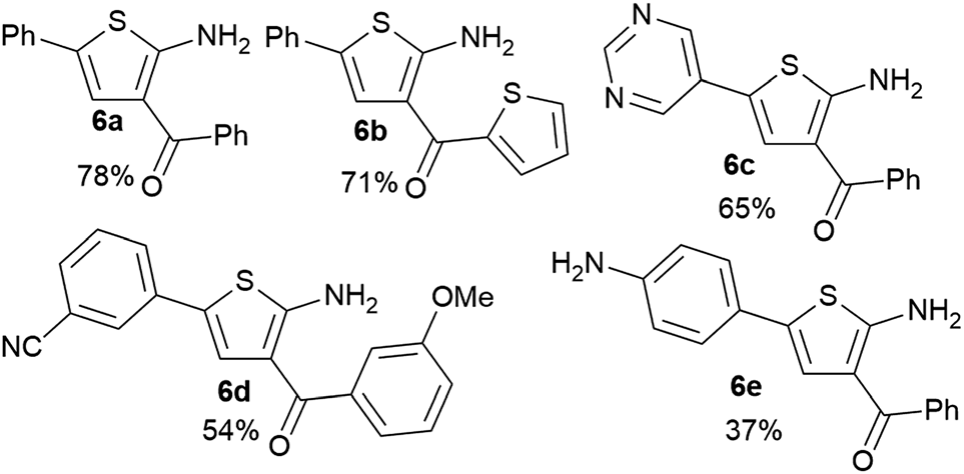

Our borylated thiophenes also proved to be useful building blocks in the synthesis of borylated bisheterocycle. Recent studies^23^ showed that substituted thieno[2,3-*b*]pyridine is an important motif in the medicinal chemistry. Borylation of thieno[2,3-*b*]pyridines has been underexplored and to the best of our knowledge, there are no previous publications covering this topic. We wondered if the borylated thieno[2,3-*b*]pyridines could be made by condensing borylated aminothiophene with ketones. We initiated our study by treating **4c** with cyclohexanone, using trimethylchlorosilane^24^ as the Lewis acid (Table 4). The cyclization reaction was finished in 1 hour and **8a** was isolated in excellent yield (95%). Several ketones were examined and it was found that the reaction was influenced by the steric effect. Hindered ketones (**8c**, **8e**) require extended reaction time (12 hours) and yields are relatively lower while cyclic ketones (**7a**, **7b**) gave excellent yields, regardless of the ring size. No protodeborylation was observed during the reaction progress for all borylated starting material and products, even though high temperature (100 °C) was required for the transformation.

**Table 4:** Synthesis of borylated thieno[2,3-*b*]pyridines

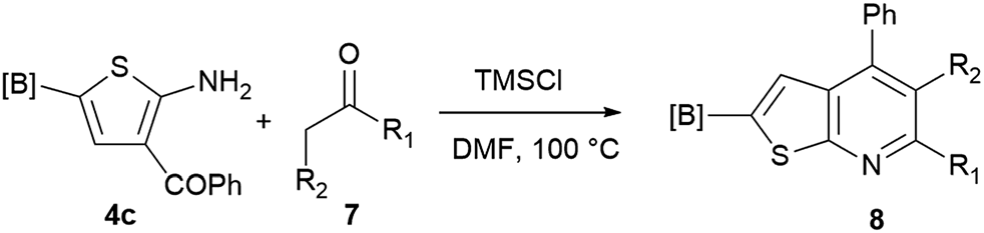			
Entry	Ketone	Product	Yield
1	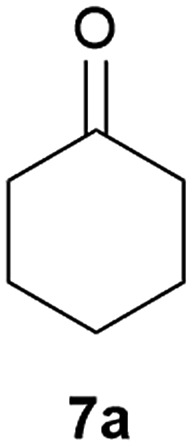	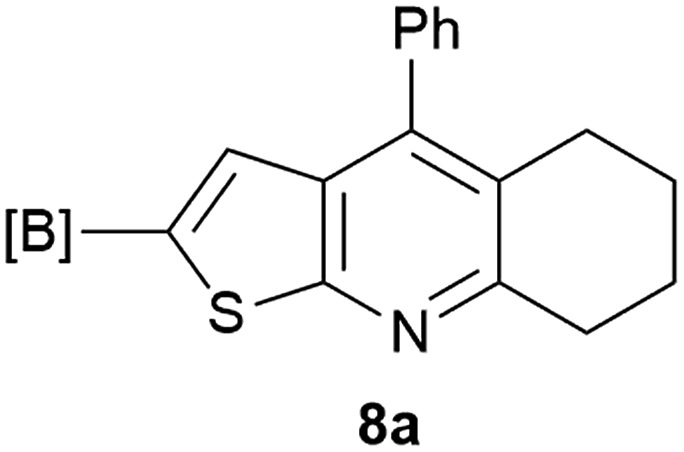	95%
2	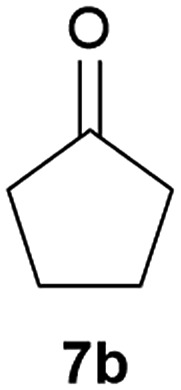	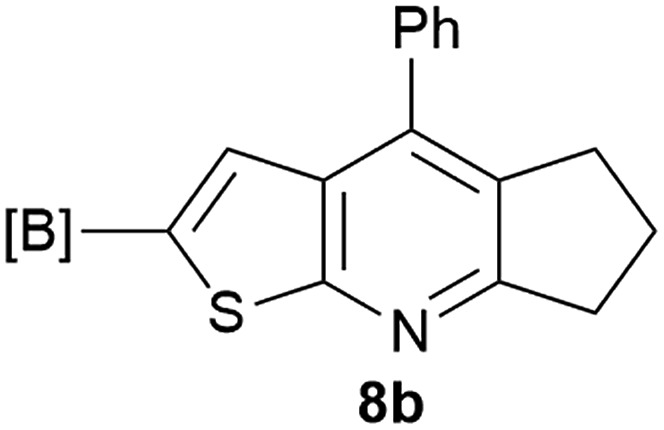	91%
3	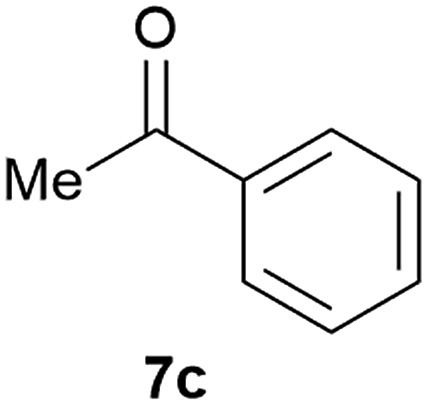	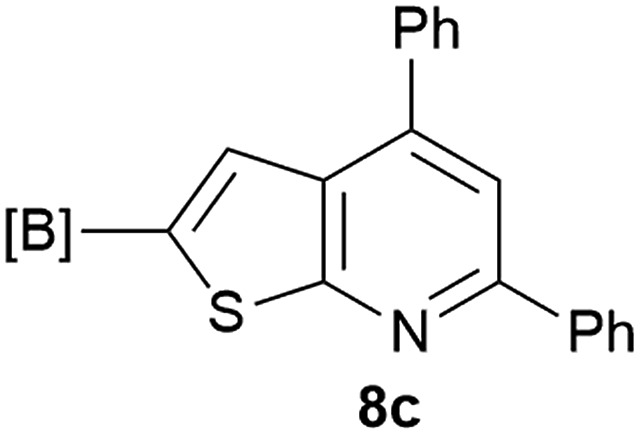	79%
4	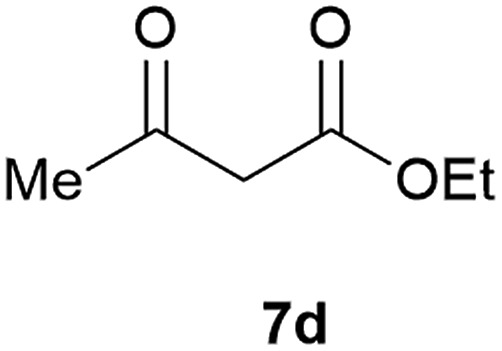	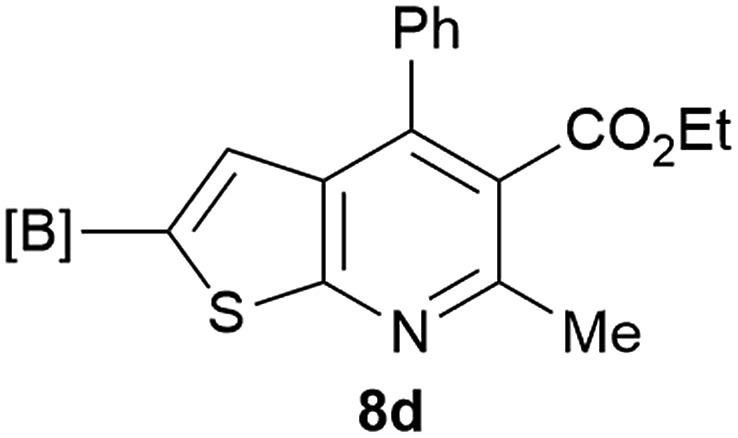	88%
5	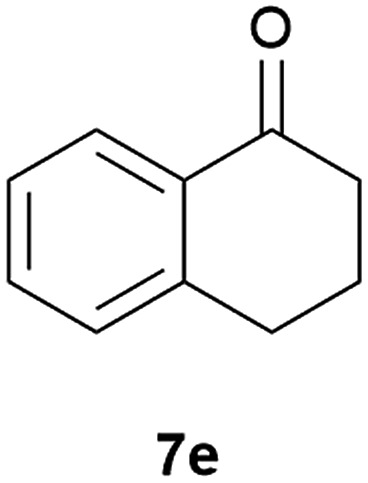	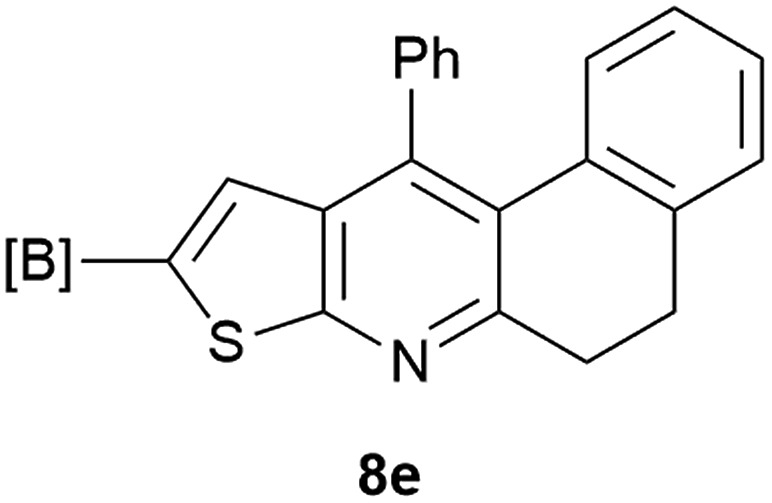	81%

## Conclusions

In summary, we have successfully developed a series of stable, previously inaccessible 3-cyanoallyl boronates. These compounds have allowed us to generate a series of borylated thiophenes in good to excellent yields. The utility of the resulting thiophene products as key intermediates toward synthetically challenging borylated bromothiophene and thieno[2,3-*b*]pyridines has been demonstrated. The successful cross-coupling of borylated aminothiophenes gave access to 3,5-disubstituted aminothiophenes, which are of interest in medicinal chemistry. Further applications of electron-poor allylboronates in synthesis are now enabled and are under intense investigation in our laboratory.
