# Supplementary material for: 3-Cyanoallyl boronates are versatile building blocks in the synthesis of polysubstituted thiophenes
Source: Chem Sci. 2017 Apr 18;8(6):4431–6. doi: 10.1039/c7sc00831g (PMC5590094; doi:10.1039/c7sc00831g)
Supplement: Supplementary file 1 [file SC-008-C7SC00831G-s001.pdf]

Electronic Supplementary Material (ESI) for Chemical Science.
This journal is © The Royal Society of Chemistry 2017

Supporting Information For

3-Cyanoallyl Boronates Are Versatile Building Blocks in the Synthesis of Polysubstituted Thiophenes

Wenjie Shao, Sherif J. Kaldas, and Andrei K. Yudin*

Davenport Research Laboratories, Department of Chemistry, University of Toronto,
80 St. George St., Toronto, ON M5S 3H6, Canada

Table of Contents

General experimental information 3
Synthesis of electron-poor allylboronates 4
Synthesis of 5-(MIDA)boryl-2-aminothiophenes 6
Synthesis of borylated bromothiophene 11
Suzuki-Miyaura cross-coupling of borylated thiophene 12
Synthesis of 2-(MIDA)boryl thieno[2,3-b]pyridines 15
Copies of ^{1}H NMR, ^{13}C NMR and ^{11}B NMR 17
References 55

General experimental information

General: Solvents used in the cross-coupling reaction were degassed with nitrogen gas prior to use. All other solvents were of reagent grade quality, and used as received. Unless otherwise noted, all reagents were purchased from commercial sources and used as received. **Chromatography:** Flash column chromatography was carried out using Silicycle 230-400 mesh silica gel. Thin-layer chromatography (TLC) was performed on Macherey Nagel precoated glassbacked TLC plates (SIL G/UV254, 0.25 mm) and visualized using a UV lamp (254 nm) or $KMnO_4$ stain in case of no UV activity. **Nuclear Magnetic Resonance Spectroscopy:** NMR spectra were recorded at 25°C on Bruker Advance III 400 MHz spectrometer, Varian Mercury 400, Agilent DD2 500, or Agilent DD2 600 instrument. Recorded shifts for protons are reported in parts per million (δ scale) downfield from tetramethylsilane and are referenced to residual protium in the NMR solvents (DMSO-d_6: 2.50 or Acetonitrile-d_3: δ 1.94, centre line). Chemical shifts for carbon resonances are reported in parts per million (δ scale) downfield from tetramethylsilane and are referenced to the carbon resonances of the solvent (DMSO-d_6: 39.52 or Acetonitrile-d_3: δ 1.32, centre line). Carbons exhibiting significant line broadening brought about by boron substituents were not reported (quadrupolar relaxation). ^{11}B NMR was recorded at 25°C on a Bruker Advance III 400 MHz spectrometer or on an Agilent DD2 600 MHz spectrometer with an Agilent OneNMR probe and referenced to an external standard of $BF_3 \cdot Et_2O$. Data are represented as follows: chemical shift δ in ppm, multiplicity (s singlet, d doublet, t triplet, q quartet, m multiplet, br broad), coupling constant *J* in Hz and integration. **Mass Spectrometry:** High resolution mass spectra were obtained on a VG 70-250S (double focusing) mass spectrometer at 70 eV or on an ABI/Sciex Qstar mass spectrometer with ESI source, MS/MS and accurate mass capabilities or on JEOL AccuTOF-DART instrument.

Synthesis of electron-poor allylboronates

2-[2-(6-methyl-4,8-dioxo-1,3,6,2-dioxazaborocan-2-yl)ethylidene]propanedinitrile (3a)

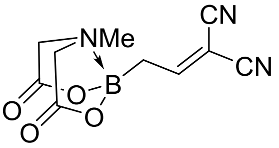

To a stirred solution of α-boryl aldehyde[1] (111 mg, 0.56 mmol) in CH_3CN (10 mL) was added malononitrile (41 mg, 0.62 mmol) and Et_2NH (0.06 mL, 0.58 mmol) successively at room temperature. The reaction mixture was stirred at 45 ℃ for 1 h before it was cooled to room temperature. Solvent was evaporated and the residue was purified by using silica gel flash column chromatography (EtOAc with 0.1% Et_3N) gave **3a** as brown solid (125 mg, 91%). TLC (EtOAc, $R_f = 0.08$); m.p 120-121 °C; ^{1}H NMR (400 MHz, DMSO-d_6) δ 8.03 – 7.90 (t, J = 9.0 Hz, 1H), 4.28 (d, J = 17.1 Hz, 2H), 4.08 (d, J = 17.1 Hz, 2H), 2.95 (s, 3H), 2.22 (d, J = 9.0 Hz, 2H); ^{13}C NMR (101 MHz, DMSO-d_6) δ 173.8, 168.2, 113.2, 111.8, 85.2, 62.0, 46.1; ^{11}B NMR (128 MHz, DMSO-d_6) δ 11.1; HRMS (DART-TOF) calculated for $[C_{10}H_{10}BN_3O_4+NH_4]^+$ 265.1108, found 265.1108.

(2E)-2-cyano-4-(6-methyl-4,8-dioxo-1,3,6,2-dioxazaborocan-2-yl)but-2-enoate (3b)

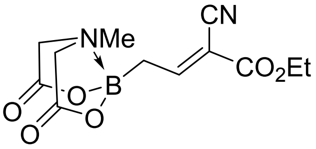

To a stirred solution of α-boryl aldehyde (100 mg, 0.50 mmol) in CH_3CN (20 mL) was added ethyl cyanoacetate (62 mg, 0.55 mmol) and Et_2NH (0.05 mL, 0.50 mmol) successively at room temperature. The reaction mixture was stirred at 45 ℃ for 2 h before it was cooled to room temperature. Solvent was evaporated and the residue was purified by using silica gel flash column chromatography (EtOAc with 0.1% Et_3N) gave **3b** as white solid (137 mg, 93%). TLC (EtOAc, $R_f = 0.14$); m.p 178-180 °C; ^{1}H NMR (400 MHz, DMSO-d_6) δ 7.83 – 7.51 (m, 1H), 4.33 – 4.17 (m, 4H), 4.07 (d, J = 17.1 Hz, 2H), 2.94 (s, 3H), 2.14 (d, J = 9.0 Hz, 2H), 1.25 (t, J = 7.1 Hz, 3H); ^{13}C NMR (101 MHz, DMSO-d_6) δ 168.3, 165.4, 161.1, 114.3, 106.9, 61.9, 61.7, 46.0, 13.9; ^{11}B NMR (128 MHz, DMSO-d_6) δ 10.9; HRMS (DART-TOF) calculated for $[C_{12}H_{15}BN_2O_6+NH_4]^+$ 312.1367, found 312.1375.

(2E)-N-benzyl-2-cyano-4-(6-methyl-4,8-dioxo-1,3,6,2-dioxazaborocan-2-yl)but-2-

enamide (3c)

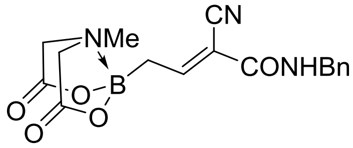

To a stirred solution of α-boryl aldehyde (100 mg, 0.25 mmol) in CH_3CN (10 mL) was added *N*-benzyl-2-cyanoacetamide (181 mg, 1 mmol) and Et_2NH (0.052 mL, 0.50 mmol) successively at room temperature. The reaction mixture was stirred at 45 °C for 2 h before the it was cooled to room temperature. Solvent was evaporated and the residue was purified by silica gel flash column chromatography (35% acetonitrile in toluene with 1% Et_3N) gave **3c** as a beige solid (149 mg, 84%). TLC (3:2 Toluene/Acetonitrile, R_f = 0.3); m.p. = 100-101 °C; ^{1}H NMR (500 MHz, DMSO-d_6) δ 8.81 (t, J = 5.9 Hz, 1H), 7.59 (t, J = 8.7 Hz, 1H), 7.34 – 7.23 (m, 5H), 4.36 (d, J = 5.9 Hz, 2H), 4.28 (d, J = 17.1 Hz, 2H), 4.09 (d, J = 17.0 Hz, 2H), 2.95 (s, 3H), 2.07 (d, J = 8.7 Hz, 2H); ^{13}C NMR (126 MHz, DMSO-d_6) δ 168.5, 160.7, 158.6, 139.1, 128.3, 127.3, 126.9, 115.3, 110.6, 61.89, 46.1, 42.8;^{11}B NMR (DMSO-d_6, 128 MHz) δ11.7; HRMS (DART-TOF) calculated for $[C_{17}H_{18}BN_3O_5+H]^+$ 356.1412, found 356.1417.

(2E)-2-cyano-4-(6-methyl-4,8-dioxo-1,3,6,2-dioxazaborocan-2-yl)but-2-enamide (3d)

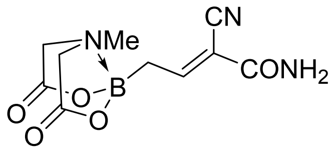

To a stirred solution of α-boryl aldehyde (100 mg, 0.50 mmol) in CH_3CN (10 mL) was added cyanoacetamide (42 mg, 0.50 mmol) and Et_2NH (0.05 mL, 0.50 mmol) successively at room temperature. The reaction mixture was stirred at 45 ℃ for 2 h before it was cooled to room temperature. Solvent was evaporated and the residue was purified by using silica gel flash column chromatography (50% Et_2O/CH_3CN to CH_3CN with 0.1% Et_3N) gave **3c** as white solid (117 mg, 88%). TLC (CH_3CN, R_f = 0.8); m.p 238-240 °C; ^{1}H NMR (400 MHz, Acetonitrile-d_3) δ 7.61 (t, J = 8.8 Hz, 1H), 4.04 (d, J = 17.0 Hz, 2H), 3.90 (d, J = 17.0 Hz, 2H), 2.96 (s, 3H), 2.15 – 2.10 (m, 4H); ^{13}C NMR (101 MHz, DMSO-d_6) δ 168.4, 162.4, 158.2, 115.4, 111.2, 61.8, 45.9; ^{11}B NMR (128 MHz, Acetonitrile-d_3) δ 11.3; HRMS (DART-TOF) calculated for $[C_{10}H_{12}BN_3O_5+H]^+$ 266.0948, found 266.0954.

Synthesis of 5-(MIDA)boryl-2-aminothiophenes

Unless otherwise noted, 5-(MIDA)boryl-2-aminothiophenes were synthesized by the following procedure: To a solution of α-boryl aldehyde (1.0 equiv) in DMF (0.05 M) was added nitrile (1.1 equiv), sulfur (2.0 equiv) and diethylamine (1.0 equiv) successively. The reaction mixture was stirred at 45℃ and monitored by TLC until the complete consumption of α-boryl aldehyde (usually 3 h for all examples except 16 h for compound **4j**). Then the reaction mixture was cooled to room temperature and the solvent was evaporated under reduced pressure. The resulting residue was purified by column chromatography using EtOAc to CH_3CN/EtOAc as eluent.

2-amino-5-(6-methyl-4,8-dioxo-1,3,6,2-dioxazaborocan-2-yl)thiophene-3-carbonitrile (4a)

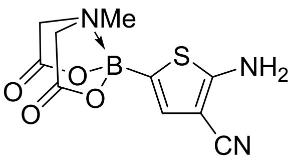

The title compound was obtained as a light brown solid 73% (102 mg) following the standard procedure using α-borylaldehyde (100 mg, 0.50 mmol), malononitrile (37 mg, 0.55 mmol), Et_2NH, (0.052 mL, 0.50 mmol), sulfur (24 mg, 0.075 mmol) and DMF (17 mL). Reaction time: 3 h. TLC (EtOAc, R_f= 0.38); m.p 271-272 ^oC; ^1H NMR (400 MHz, Acetonitrile-d_3) δ 6.88 (s, 1H), 5.68 (s, 2H), 4.02 (d, J = 17.1 Hz, 2H), 3.87 (d, J = 17.1 Hz, 2H), 2.68 (s, 3H); ^{13}C NMR (DMSO-d_6, 126 MHz) δ 168.8, 167.8, 131.8, 116.4, 85.1, 61.3, 47.2; ^{11}B NMR (DMSO-d_6, 128 MHz) δ 10.8; HRMS (ESI+) calculated for $[C_{10}H_{10}BN_3O_4S+H]^+$ 279.0575, found 279.0594.

ethyl 2-amino-5-(6-methyl-4,8-dioxo-1,3,6,2-dioxazaborocan-2-yl)thiophene-3-carboxylate (4b)

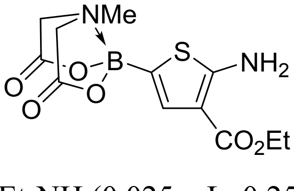

The title compound was obtained as brown solid in 81% (61 mg) following the standard procedure using α-boryl aldehyde (49 mg, 0.25 mmol), ethyl cyanoacetate (31 mg, 0.27 mmol), Et_2NH (0.025 mL, 0.25 mmol), sulfur (16 mg, 0.50 mmol) and DMF (6 mL). Reaction time: 3 h. TLC (EtOAc, R_f = 0.43); m.p 172-173 °C; ^1H NMR (500 MHz, DMSO-d_6) δ 7.30 (s, 2H), 6.92 (s, 1H), 4.26 (d, J = 17.2 Hz, 2H), 4.15 (q, J = 7.1 Hz, 2H), 4.07 (d, J = 17.2 Hz, 2H), 2.65 (s, 3H), 1.24 (t, J = 7.1 Hz, 3H); ^{13}C NMR (126 MHz, DMSO-d_6) δ 169.4, 167.4, 164.8, 132.1, 106.2, 61.7, 59.3, 47.7, 14.9; ^{11}B NMR (128 MHz, DMSO-

d_6) δ 11.1; HRMS (DART-TOF) calculated for $[C_{12}H_{15}BN_2O_6S+H]^+$ 327.0822, found 327.0820.

2-(5-amino-4-benzoylthiophen-2-yl)-6-methyl-1,3,6,2-dioxazaborocane-4,8-dione (4c)

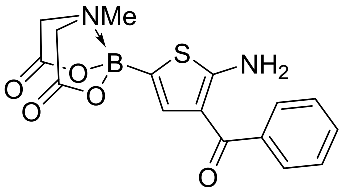

The title compound was obtained as brown solid in 86% (773 mg) following the standard procedure using α-boryl aldehyde (500 mg, 2.51 mmol), nitrile **2c** (400 mg, 2.76 mmol), Et_2NH (0.26 mL, 2.51 mmol), sulfur (161 mg, 5.02 mmol) and DMF (50 mL). Reaction time: 3 h. TLC (EtOAc, R_f = 0.47); m.p 223-225 °C; ^{1}H NMR (400 MHz, DMSO-d_6) δ 8.37 (s, 2H), 7.58 (dd, J = 7.7, 1.9 Hz, 2H), 7.52 – 7.44 (m, 3H), 6.82 (s, 1H), 4.25 (d, J = 17.3 Hz, 2H), 4.04 (d, J = 17.2 Hz, 2H), 2.67 (s, 3H); ^{13}C NMR (126 MHz, DMSO-d_6) δ 189.7, 170.5, 169.4, 141.4, 133.6, 130.9, 128.6, 128.2, 115.3, 61.8, 47.8; ^{11}B NMR (96 MHz, Acetonitrile-d_3) δ 10.4; HRMS (DART-TOF) calculated for $[C_{16}H_{15}BN_2O_5S+H]^+$ 359.0873, found 359.0878.

2-amino-5-(6-methyl-4,8-dioxo-1,3,6,2-dioxazaborocan-2-yl)thiophene-3-carboxamide (4d)

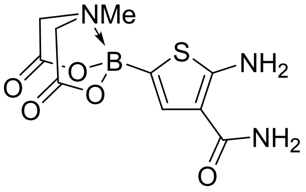

The title compound was obtained as red solid in 85% (127 mg) following the standard procedure using α-boryl aldehyde (100 mg, 0.50 mmol), nitrile **2d** (46 mg, 0.55 mmol), Et_2NH (0.05 mL, 0.50 mmol), sulfur (32 mg, 1.0 mmol) and DMF (10 mL). Reaction time: 3 h. TLC (acetone, R_f = 0.30); m. p. 180-181 °C; ^{1}H NMR (500 MHz, Acetonitrile-d_3) δ 7.10 (s, 1H), 6.66 (s, 2H), 4.04 (d, J = 17.0 Hz, 2H), 3.92 – 3.80 (m, 2H), 2.70 (s, 3H); ^{13}C NMR (126 MHz, Acetonitrile-d_3) δ 168.1, 167.7, 165.4, 131.2, 109.4, 61.1, 47.1; ^{11}B NMR (128 MHz, Acetonitrile-d_3) δ 10.4; HRMS (DART-TOF) calculated for $[C_{10}H_{12}BN_3O_5S+H]^+$ 298.0669, found 298.0672.

2-[5-amino-4-(4-fluorobenzoyl)thiophen-2-yl]-6-methyl-1,3,6,2-dioxazaborocane-4,8-dione (4e)

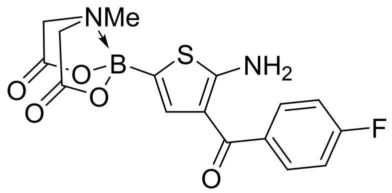

The title compound was obtained as pale yellow solid in 91% (172 mg) following the standard procedure using α-boryl aldehyde (100 mg, 0.50 mmol), nitrile **2e** (90 mg, 0.55 mmol), Et_2NH (0.05 mL, 0.50 mmol), sulfur (32 mg, 1.0 mmol) and DMF (12 mL). Reaction time: 3 h. TLC (CH_3CN/Et_2O = 1:2, R_f = 0.70); m.p 248-249 °C; ^{1}H NMR (400 MHz, DMSO-d_6) δ 8.36 (s, 2H), 7.66 (dd, J = 8.8, 5.6 Hz, 2H), 7.30 (t, J = 8.9 Hz, 2H), 6.82 (s, 1H), 4.26 (d, J = 16.8 Hz, 2H), 4.05 (d, J = 17.2 Hz, 2H), 2.67 (s, 3H); ^{13}C NMR (101 MHz, DMSO-d_6) δ 187.8, 170.1, 168.9, 137.4, 132.9, 130.4, 115.2, 115.0, 114.7, 61.3, 47.3; ^{11}B NMR (192 MHz, CD_3CN) δ 10.3; HRMS (DART-TOF) calculated for $[C_{16}H_{14}BFN_2O_5S+H]^+$ 377.0778, found 327.0770.

2-[5-amino-4-(4-nitrobenzoyl)thiophen-2-yl]-6-methyl-1,3,6,2-dioxazaborocane-4,8-dione (4f)

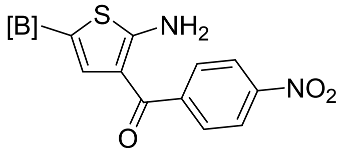

The title compound was obtained as orange solid in 84% (151 mg) following the standard procedure using α-boryl aldehyde (89 mg, 0.45 mmol), nitrile **2f** (94 mg, 0.49 mmol), Et_2NH (0.05 mL, 0.45 mmol), sulfur (29 mg, 0.90 mmol) and DMF (9 mL). Reaction time: 3 h. TLC (EtOAc, R_f = 0.33); m.p 270-271 °C; ^{1}H NMR (400 MHz, DMSO-d_6) δ 8.53 (s, 2H), 8.30 (d, J = 8.7 Hz, 2H), 7.82 (d, J = 8.7 Hz, 2H), 6.74 (s, 1H), 4.26 (d, J = 17.2 Hz, 2H), 4.04 (d, J = 17.2 Hz, 2H), 2.68 (s, 3H); ^{13}C NMR (126 MHz, DMSO-d_6) δ 187.4, 171.2, 169.3, 148.7, 146.9, 132.8, 129.4, 124.0, 115.1, 61.8, 47.8; ^{11}B NMR (128 MHz, DMSO-d_6) δ 11.0; HRMS (DART-TOF) calculated for $[C_{16}H_{14}BN_3O_7S+H]^+$ 404.0723, found 404.0723.

2-[5-amino-4-(thiophene-2-carbonyl)thiophen-2-yl]-6-methyl-1,3,6,2-dioxazaborocane-4,8- dione (4g)

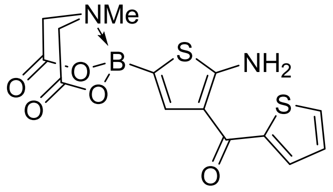

The title compound was obtained in 86% (215 mg) following the standard procedure using α-boryl aldehyde (137 mg, 0.69 mmol), nitrile **2g** (100 mg, 0.69 mmol), Et_2NH (0.07 mL, 0.69 mmol), sulfur (44 mg, 1.38 mmol)

and DMF (14 mL). Reaction time: 3 h. TLC (EtOAc, R_f = 0.29); m. p 250-252 °C; ^{1}H NMR (400 MHz, Acetonitrile-d_3) δ 7.76 (dd, J = 3.7, 1.1 Hz, 1H), 7.71 (dd, J = 5.0, 1.1 Hz, 1H), 7.46 (s, 2H), 7.42 (s, 1H), 7.20 (dd, J = 5.0, 3.8 Hz, 1H), 4.06 (d, J = 17.0 Hz, 2H), 3.92 (d, J = 17.1 Hz, 2H), 2.76 (s, 3H); ^{13}C NMR (101 MHz, DMSO-d_6) δ 179.6, 170.1, 168.9, 145.2, 131.9, 131.7, 130.5, 128.0, 114.2, 61.4, 47.3; ^{11}B NMR (128 MHz, Acetonitrile-d_3) δ 10.1; HRMS (DART-TOF) calculated for $[C_{14}H_{13}BN_2O_6S_2+H]^+$ 365.0437, found 365.0436.

2-amino-5-(6-methyl-4,8-dioxo-1,3,6,2-dioxazaborocan-2-yl)thiophene-3-carbothioamide (4h)

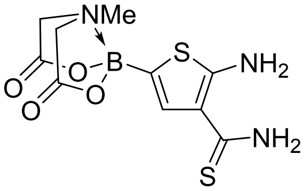

The title compound was obtained as brown thick oil in 79% (124 mg) following the standard procedure using α-boryl aldehyde (100 mg, 0.50 mmol), nitrile **2h** (55 mg, 0.55 mmol), Et_2NH (0.05 mL, 0.50 mmol), sulfur (32 mg, 1.0 mmol) and DMF (10 mL). Reaction time: 5 h. TLC (EtOAc, R_f = 0.05); m.p 166-168 °C; ^{1}H NMR (400 MHz, Acetonitrile-d_3) δ 7.94 (s, 1H), 7.15 (s, 1H), 6.91 (s, 1H), 5.72 (br s, 2H), 4.05 (d, J = 17.2 Hz, 2H), 3.89 (m, 2H), 2.71 (s, 3H); ^{13}C NMR (126 MHz, Acetonitrile-d_3) δ 190.9, 168.07, 168.0, 167.8, 166.9, 132.3, 129.5, 115.4, 113.6, 88.0, 61.9, 61.4, 61.3, 61.2, 47.3, 47.2; ^{11}B NMR (128 MHz, Acetonitrile-d_3) δ 9.8; HRMS (DART-TOF) calculated for $[C_{10}H_{12}BN_3O_4S_2+H]^+$ 313.0471, found 313.0458.

2-[5-amino-4-(1-benzofuran-2-carbonyl)thiophen-2-yl]-6-methyl-1,3,6,2-dioxazaborocane-4,8-dione(4i)

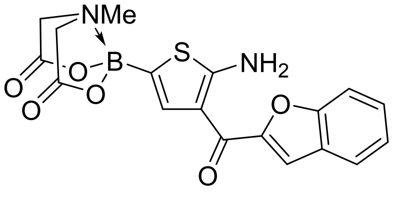

The title compound was obtained as brown solid in 82% (164 mg) following the standard procedure using α-boryl aldehyde (100 mg, 0.50 mmol), nitrile **2i** (184 mg, 1.0 mmol), Et_2NH (0.05 mL, 0.50 mmol), sulfur (32 mg, 1.0 mmol) and DMF (12 mL). Reaction time: 3 h. TLC (EtOAc, R_f = 0.34); m.p 260-261 °C; ^{1}H NMR (400 MHz, Acetonitrile-d_3) δ 7.80 (d, J = 0.6 Hz, 1H), 7.78 (dd, J = 1.3, 0.7 Hz, 1H), 7.73 (br s, 1H), 7.64 (dd, J = 8.4, 0.9 Hz, 1H), 7.59 (d, J = 1.0 Hz, 1H), 7.51 (ddd, J = 8.4, 7.2, 1.3 Hz, 1H), 7.36 (ddd, J = 8.0, 7.2, 1.0 Hz, 1H), 4.09

(d, J = 17.0 Hz, 2H), 3.94 (d, J = 17.1 Hz, 2H), 2.78 (s, 3H); ^{13}C NMR (101 MHz, DMSO-d_6) δ 175.5, 171.3, 168.9, 154.4, 153.6, 131.7, 127.1, 126.9, 123.7, 122.9, 114.4, 111.8, 111.5, 61.3, 47.3; ^{11}B NMR (128 MHz, Acetonitrile-d_3) δ 10.1; HRMS (DART-TOF) calculated for $[C_{18}H_{15}BN_2O_6S+H]^+$ 399.0822, found 399.0821.

2-[5-amino-4-(benzenesulfonyl)thiophen-2-yl]-6-methyl-1,3,6,2-dioxazaborocane-4,8-dione (4j)

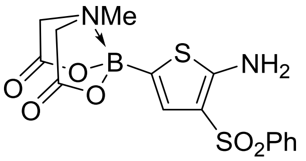

The title compound was obtained as a red powder in 33% (33 mg) following the standard procedure using α-borylaldehyde (50 mg, 0.253 mmol), nitrile **2j** (100 mg, 0.56 mmol), Et_2NH (0.026 mL, 0.25 mmol), sulfur (21 mg, 0.63 mmol) and DMF (7 mL). Reaction time : 16 h. TLC (EtOAc, R_f = 0.4); m.p. = 234-236 °C; ^{1}H NMR (400 MHz, Acetonitrile-d_3) δ 7.92 – 7.89 (m, 2H), 7.65 – 7.61 (m, 1H), 7.59 – 7.54 (m, 2H), 6.96 (s, 1H), 6.15 (br s, 2H), 4.01 (d, J = 17.0 Hz, 2H), 3.87 (d, J = 17.1 Hz, 2H), 2.67 (s, 3H); ^{13}C NMR (DMSO-d_6) δ 168.84, 162.87, 143.25, 132.78, 130.56, 129.33, 125.84, 111.91, 61.51, 47.43; ^{11}B NMR (DMSO-d_6, 128 MHz) 9.97; HRMS (DART-TOF) calculated for $[C_{15}H_{15}BN_2O_6S_2+H]^+$ 395.0537, found 395.0542.

2-[5-amino-4-(1H-pyrrole-2-carbonyl)-1H-pyrrol-2-yl]-6-methyl-1,3,6,2-dioxazaborocane-4,8-dione (4k)

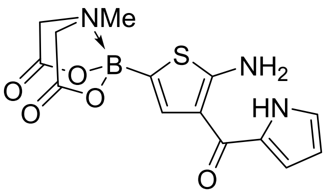

The title compound was obtained as pale brown solid in 88% (153 mg) following the standard procedure using α-boryl aldehyde (100 mg, 0.50 mmol), nitrile **2k** (74 mg, 0.55 mmol), Et_2NH (0.05 mL, 0.50 mmol), sulfur (32 mg, 1.0 mmol) and DMF (12 mL). Reaction time: 3 h. TLC (EtOAc, R_f = 0.23); m.p 258-260 °C; ^{1}H NMR (400 MHz, Acetonitrile-d_3) δ 10.06 (br s, 1H), 7.47 (s, 1H), 7.29 (s, 1H), 7.05 (td, J = 2.7, 1.3 Hz, 1H), 6.93 (ddd, J = 3.8, 2.5, 1.3 Hz, 1H), 6.43 – 6.21 (m, 1H), 4.06 (d, J = 17.1 Hz, 2H), 3.92 (d, J = 17.1 Hz, 2H), 2.76 (s, 3H); ^{13}C NMR (101 MHz, DMSO-d_6) δ 177.9, 168.9, 168.6, 132.2, 131.6, 123.2, 114.5, 114.3, 109.3, 61.4, 47.3; ^{11}B NMR (128 MHz, Acetonitrile-d_3) δ 10.3; HRMS (DART-TOF) calculated for $[C_{14}H_{14}BN_3O_5S+H]^+$ 348.0825, found 348.0825.

2-[5-amino-4-(3-methoxybenzoyl)thiophen-2-yl]-6-methyl-1,3,6,2-dioxazaborocane-4,8-dione (4l)

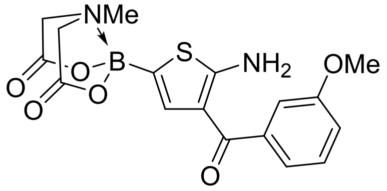

The title compound was obtained as pale brown solid in 90% (194 mg) following the standard procedure using α-boryl aldehyde (111 mg, 0.56 mmol), nitrile **2l** (108 mg, 0.62 mmol), Et_2NH (0.06 mL, 0.62 mmol), sulfur (39 mg, 1.22 mmol) and DMF (20 mL). Reaction time: 3 h. TLC (EtOAc, R_f = 0..31); m.p. 230-232 °C; ^{1}H NMR (500 MHz, Acetonitrile-d_3) δ 7.51 (s, 2H), 7.41 (ddd, J = 8.2, 7.5, 0.4 Hz, 1H), 7.23 (ddd, J = 7.5, 1.5, 1.0 Hz, 1H), 7.19 (dd, J = 2.6, 1.5 Hz, 1H), 7.10 (ddd, J = 8.2, 2.7, 1.0 Hz, 1H), 7.00 (s, 1H), 4.03 (d, J = 17.0 Hz, 2H), 3.87 (d, J = 17.1 Hz, 2H), 3.85 (s, 3H), 2.73 (s, 3H); ^{13}C NMR (101 MHz, DMSO-d_6) δ 188.8, 170.1, 168.9, 158.9, 142.3, 133.2, 129.3, 120.0, 116.3, 114.8, 112.8, 61.3, 55.1, 47.3; ^{11}B NMR (128 MHz, Acetonitrile-d_3) δ 10.1; HRMS (DART-TOF) calculated for $[C_{17}H_{17}BN_2O_6S+H]^+$ 389.0978, found 389.0974.

Synthesis of borylated bromothiophene

2-bromo-5-(6-methyl-4,8-dioxo-1,3,6,2-dioxazaborocan-2-yl)thiophene-3-carboxamide (5a)

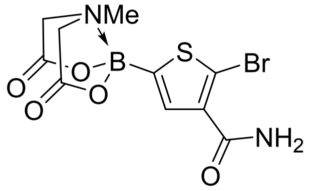

To a stirred solution of aminothiophene (**4d**, 37 mg, 0.124 mmol) in CH_3CN (3 mL) was added $CuBr_2$ (42 mg, 0.187 mmol) and *t*-BuONO (16 mg, 0.155 mmol) successively at 0 °C. The cold bath was left in place but not recharged, and the reaction mixture was stirred overnight. The mixture was filtered through a short pad of celite and the filtrate was concentrated under reduced pressure. The residue was purified by using silica gel flash chromatography (50% acetone/hexanes to 75% acetone/hexanes) to afford the title compound as brown solid (28 mg, 63%). TLC (EtOAc, R_f = 0.43); m.p 173-174 °C. ^{1}H NMR (400 MHz, Acetonitrile-d_3) δ 7.41 (s, 1H), 6.66 (br s, 1H), 6.18 (br s, 1H), 4.12 (d, J = 17.1 Hz, 2H), 3.97 (d, J = 17.2 Hz, 2H), 2.72 (s, 3H); ^{13}C NMR (126 MHz, Acetonitrile-d_3) δ 167.8, 163.8, 136.8, 134.1, 117.5, 61.6, 47.6; ^{11}B NMR (128 MHz, Acetonitrile-d_3) δ 9.7; HRMS (DART-TOF) calculated for $[C_{10}H_{10}BBrN_2O_5S+H]^+$ 359.9698, found 359.9698.

Suzuki-Miyaura cross-coupling of borylated thiophene

3-benzoyl-5-phenylthiophen-2-amine[2] (6a)

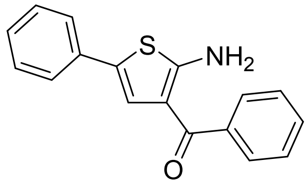

To screw cap vial affixed with a rubber septum was added RuPhos Pd G3 (17 mg, 0.02 mmol), followed by aminothiopene **4c** (85 mg, 0.24 mmol) and sodium carbonate (64 mg, 0.6 mmol). The vial was then evacuated and backfilled with nitrogen three times. A degassed solution of DMF/H_2O (10/1, 2.0 mL) was then added through the septum, followed by degassed bromobenzene (0.021 mL, 0.2 mmol). The reaction mixture was then heated to 60 ^oC in a sand-bath. The reaction was stirred for 24 hours at which time TLC analysis indicated that the starting material had been consumed. The crude reaction mixture was then filtered through a small plug of silica before being concentrated *in vacuo*. The resulting residue was purified by column chromatography using 5-20% EtOAc/Hexanes as the eluent. The desired product was isolated in 78% yield (43.4 mg). TLC (1:4 EtOAc/Hexanes, $R_f = 0.40$); ^1H NMR (400 MHz, Chloroform-*d*) δ 7.71 (d, J = 8.1 Hz, 8H), 7.49 (d, J = 27.7 Hz, 4H), 7.39 (d, J = 9.3 Hz, 7H), 7.31 (d, J = 15.4 Hz, 2H), 7.26 (d, J = 1.5 Hz, 2H), 7.23 – 7.19 (m, 1H), 7.12 (s, 1H); ^{13}C NMR (126 MHz, $CDCl_3$) δ 191.4, 165..9, 140.8, 133.9, 130.9, 128.9, 128.4, 128.2, 126.9, 124.9, 124.1, 122.9, 116.1.

5-phenyl-3-(thiophene-2-carbonyl)thiophen-2-amine (6b)

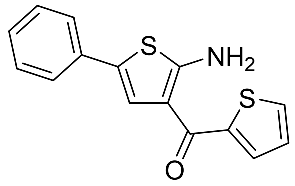

To screw cap vial affixed with a rubber septum was added RuPhos Pd G3 (9 mg, 0.011 mmol), followed by aminothiopene **4g** (43 mg, 0.12 mmol) and sodium carbonate (25 mg, 0.30 mmol). The vial was then evacuated and backfilled with nitrogen three times. A degassed solution of DMF/H_2O (10/1, 1.0 mL) was then added through the septum, followed by degassed bromobenzene (0.01 mL, 0.10 mmol). The reaction mixture was then heated to 60 ^oC in a sand-bath. The reaction was stirred for 24 hours at which time TLC analysis indicated that the starting material had been consumed. The crude reaction mixture was then filtered through a small plug of silica before being

concentrated *in vacuo*. The resulting residue was purified by column chromatography using 5-20% EtOAc/Hexanes as the eluent. The desired product was isolated in 71% yield (20 mg). TLC (1:4 EtOAc/Hexanes, R_f = 0.30); m.p = 160-162 °C; ^{1}H NMR (400 MHz, Chloroform-*d*) δ 7.75 (dd, *J* = 3.7, 1.1 Hz, 1H), 7.61 (dd, *J* = 5.0, 1.1 Hz, 1H), 7.50 (s, 1H), 7.49 – 7.44 (m, 2H), 7.35 (dd, *J* = 8.5, 6.9 Hz, 2H), 7.24 – 7.20 (m, 1H), 7.16 (dd, *J* = 5.0, 3.7 Hz, 1H), 7.00 (br s, 2H); ^{13}C NMR (101 MHz, Chloroform-*d*) δ 181.4, 165.8, 145.0, 133.8, 131.3, 130.9, 128.9, 127.5, 126.9, 124.9, 124.6, 121.6, 115.5; HRMS (DART-TOF) calculated for $[C_{15}H_{11}NOS_2+H]^+$ 286.0360, found 286.0360.

3-benzoyl-5-(pyrimidin-5-yl)thiophen-2-amine (6c)

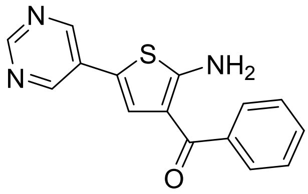

To screw cap vial affixed with a rubber septum was added RuPhos Pd G3 (7 mg, 0.008 mmol), followed by aminothiopene **4c** (35 mg, 0.098 mmol), sodium carbonate (28 mg, 0.26 mmol) and 5-bromopyrimidine (14 mg, 0.088 mmol). The vial was then evacuated and backfilled with nitrogen three times. A degassed solution of DMF/H_2O (10/1, 1.5 mL) was then added through the septum. The reaction mixture was then heated to 60 °C in a sand-bath. The reaction was stirred for 24 hours at which time TLC analysis indicated that the starting material had been consumed. The crude reaction mixture was concentrated *in vacuo*. The resulting residue was purified by column chromatography using 50% EtOAc/Hexanes as the eluent. The desired product was isolated in 65% yield (16 mg). TLC (2:1 EtOAc/Hexanes, R_f = 0.30); m.p = 165-167 °C; ^{1}H NMR (400 MHz, Chloroform-*d*) δ 9.03 (s, 1H), 8.74 (s, 2H), 7.81 – 7.63 (m, 2H), 7.60 – 7.40 (m, 3H), 7.26 (s, 1 H), 7.26 – 7.21 (br s, 2H); ^{13}C NMR (101 MHz, Chloroform-*d*) δ 191.3, 166.5, 156.2, 152.2, 140.3, 131.2, 128.4, 128.4, 128.0, 126.0, 116.2, 115.6; HRMS (DART-TOF) calculated for $[C_{15}H_{11}N_3OS+H]^+$ 282.0701, found 282.0702.

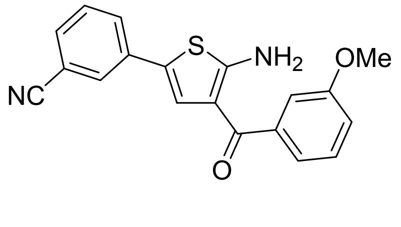

3-[5-amino-4-(3-methoxybenzoyl)thiophen-2-yl]benzonitrile (6d)

To screw cap vial affixed with a rubber septum was added RuPhos Pd G3 (8 mg, 0.009 mmol), followed by aminothiopene **4l** (37 mg, 0.095 mmol), sodium carbonate (30 mg, 0.28 mmol) and 3-bromobenzonitrile (17 mg, 0.093 mmol). The vial was then evacuated and backfilled with nitrogen three times. A degassed solution of DMF/H_2O (10/1, 2.0 mL) was then added through the septum. The reaction mixture was then heated to 60 °C in a sand-bath. The reaction was stirred for 24 hours at which time TLC analysis indicated that the starting material had been consumed. The crude reaction mixture was concentrated *in vacuo* and dry-loaded onto Celite. The resulting residue was purified by reverse-phase flash column chromatography. The desired product was isolated in 54% yield (17 mg) as light yellow solid. TLC (1:4 EtOAc/Hexanes, R_f = 0.08); m.p = 181-185 °C; ^{1}H NMR (400 MHz, Acetonitrile-d_3) δ 7.83 (ddd, J = 2.1, 1.5, 0.6 Hz, 1H), 7.75 – 7.66 (m, 3H), 7.55 (dt, J = 7.7, 1.4 Hz, 1H), 7.52 – 7.40 (m, 2H), 7.33 (s, 1H), 7.28 (ddd, J = 7.5, 1.5, 1.0 Hz, 1H), 7.22 (dd, J = 2.7, 1.5 Hz, 1H), 7.14 (ddd, J = 8.3, 2.7, 1.0 Hz, 1H), 3.87 (s, 3H); ^{13}C NMR (151 MHz, Acetonitrile-d_3) δ 190.3, 167.0, 159.5, 135.1, 129.8, 129.6, 129.4, 128.6, 127.7, 124.7, 120.3, 118.4, 116.7, 115.2, 112.9, 112.7, 55.1; HRMS (DART-TOF) calculated for $[C_{19}H_{14}N_2O_2S+H]^+$ 335.0849, found 335.0841.

5-(4-aminophenyl)-3-benzoylthiophen-2-amine (6e)

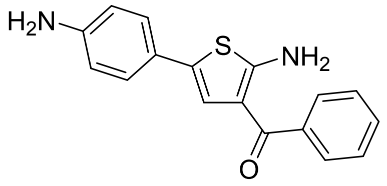

To screw cap vial affixed with a rubber septum was added RuPhos Pd G3 (8 mg, 0.009 mmol), followed by aminothiopene **4c** (40 mg, 0.112 mmol), sodium carbonate (30 mg, 0.28 mmol) and 4-bromoaniline (16 mg, 0.093 mmol). The vial was then evacuated and backfilled with nitrogen three times. A degassed solution of DMF/H_2O (10/1, 1.5 mL) was then added through the septum. The reaction mixture was then heated to 60 °C in a sand-bath. The reaction was stirred for 24 hours at which time TLC analysis indicated that the starting material had been consumed. The crude reaction mixture was concentrated *in vacuo* and dry-loaded onto Celite. The resulting residue was purified by reverse-phase flash column chromatography. The desired product was isolated in 37% yield (10 mg) as

light yellow solid. The product is not stable and color changed from light yellow dark brown. Dark brown precipitate was observed during the measurement of NMR. TLC (1:3 EtOAc/Hexanes, $R_f = 0.2$); ^{1}H NMR (500 MHz, Acetonitrile-d_3) δ 7.75 – 7.64 (m, 3H), 7.59 – 7.44 (m, 6H), 7.26 – 7.12 (m, 2H), 6.92 (s, 1H), 6.69 – 6.61 (m, 2H); ^{13}C NMR (126 MHz, Acetonitrile-d_3) δ 190.1, 165.6, 146.5, 141.1, 130.5, 129.3, 128.2, 127.9, 125.7, 124.2, 123.3, 119.5, 114.9; HRMS (DART-TOF) calculated for $[C_{17}H_{14}N_2OS+H]^+$ 295.0905, found 295.0900.

Synthesis of 2-(MIDA)boryl thieno[2,3-b]pyridines

Unless otherwise noted, 2-(MIDA)boryl thieno[2,3-b]pyridines were synthesized by the following procedure: To a solution of 5-(MIDA)boryl-2-aminothiophene (30 mg, 0.084 mmol) in DMF (3 mL) was added ketone (0.09 mmol, 1.1 equiv) and trimethylchlorosilane (0.42 mmol, 5 equiv) successively. The reaction mixture was stirred at 100 °C and monitored by LCMS until the complete consumption of the aminothiophene. Then the reaction mixture was cooled to room temperature and the solvent was evaporated under reduced pressure. The resulting residue was purified by column chromatography using EtOAc to CH_3CN/Et_2O as eluent.

6-methyl-2-{4-phenyl-5H,6H,7H,8H-thieno[2,3-b]quinolin-2-yl}-1,3,6,2-dioxazaborocane-4,8-dione (8a)

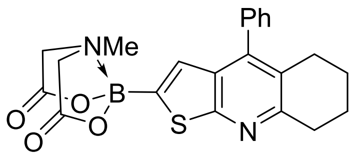

The title compound was obtained as pale yellow solid in 95% (33 mg) following the standard procedure. Reaction time: 1 h. TLC (EtOAc, $R_f = 0.41$); m.p 322-325 °C; ^{1}H NMR (500 MHz, Acetonitrile-d_3) δ 7.65 – 7.46 (m, 3H), 7.44 – 7.33 (m, 2H), 6.96 (s, 1H), 4.09 (d, J = 17.1 Hz, 2H), 3.93 (d, J = 17.0 Hz, 2H), 3.08 (t, J = 6.6 Hz, 2H), 2.69 (s, 3H), 2.63 (t, J = 6.4 Hz, 2H), 1.94 – 1.90 (m, 2H), 1.80 – 1.75 (m, 2H); ^{13}C NMR (126 MHz, Acetonitrile-d_3) δ 167.9, 161.2, 156.2, 144.3, 137.2, 131.6, 128.8, 128.5, 128.0, 127.3, 125.9, 61.7, 47.6, 33.2, 27.0, 22.7, 22.5; ^{11}B NMR (128 MHz, Acetonitrile-d_3) δ 10.9; HRMS (DART-TOF) calculated for $[C_{22}H_{21}BN_2O_4S+H]^+$ 421.1393, found 421.1398.

6-methyl-2-{8-phenyl-4-thia-2-azatricyclo[7.3.0.0³,⁷]dodeca-1(9),2,5,7-tetraen-5-yl}-1,3,6,2-dioxazaborocane-4,8-dione (8b)

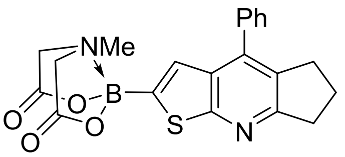

The title compound was obtained as pale yellow solid in 91% (31 mg) following the standard procedure. Reaction time: 1 h. TLC (EtOAc, R_f = 0.38); m.p. 305-306 °C; ^{1}H NMR (400 MHz, Acetonitrile-d_3) δ 7.66 – 7.43 (m, 5H), 7.29 (s, 1H), 4.11 (d, J = 17.1 Hz, 2H), 3.95 (d, J = 17.1 Hz, 2H), 3.12 (t, J = 7.6 Hz, 2H), 2.96 (t, J = 7.3 Hz, 2H), 2.70 (s, 3H), 2.21-2.13 (m, 2 H); ^{13}C NMR (126 MHz, Acetonitrile-d_3) δ 167.9, 165.0, 163.3, 140.5, 137.0, 131.5, 130.7, 128.9, 128.6, 128.3, 127.0, 61.7, 47.6, 34.1, 29.5, 23.6; ^{11}B NMR (128 MHz, Acetonitrile-d_3) δ 10.5; HRMS (DART-TOF) calculated for $[C_{21}H_{19}BN_2O_4S+H]^+$ 407.1237, found 407.1240.

2-{4,6-diphenylthieno[2,3-b]pyridin-2-yl}-6-methyl-1,3,6,2-dioxazaborocane-4,8-dione (8c)

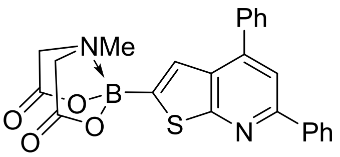

The title compound was obtained as pale yellow solid in 79% (29 mg) following the standard procedure. Reaction time: 12 h. TLC (EtOAc, Rf = 0.49); m.p > 350 °C; ^{1}H NMR (400 MHz, Acetonitrile-d_3) δ 8.23 – 8.04 (m, 2H), 7.80 (s, 1H), 7.74 – 7.62 (m, 2H), 7.55 – 7.30 (m, 6H), 4.01 (d, J = 17.1 Hz, 2H), 3.86 (d, J = 17.2 Hz, 2H), 2.62 (s, 3H); ^{13}C NMR (126 MHz, Acetonitrile-d_3) δ 167.9, 165.3, 154.4, 145.7, 138.8, 138.4, 130.7, 129.2, 128.9, 128.9, 128.9, 128.8, 116.4, 61.8, 47.7; ^{11}B NMR (128 MHz, Acetonitrile-d_3) δ 11.2; HRMS (DART-TOF) calculated for $[C_{24}H_{19}BN_2O_4S+H]^+$ 443.1237, found 443.1250.

6-methyl-2-(6-methyl-4,8-dioxo-1,3,6,2-dioxazaborocan-2-yl)-4-phenylthieno[2,3-b]pyridine-5-carboxylate (8d)

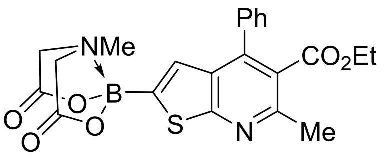

The title compound was obtained as pale yellow solid in 88% (33 mg) following the standard procedure. Reaction time: 12 h. TLC (EtOAc, R_f = 0.33); m.p > 350 °C; ^{1}H NMR (400 MHz, Acetonitrile-d_3) δ 7.58 – 7.50 (m, 3H), 7.49 – 7.44 (m, 2H), 7.24 (s, 1H), 4.26 – 4.07 (m, 4H), 3.95 (d, J = 17.1 Hz, 2H), 2.71 (s, 3H), 2.68 (s,

3H), 1.01 (t, J = 7.1 Hz, 3H); ^{13}C NMR (101 MHz, Acetonitrile-d_3) δ 168.2, 167.5, 163.9, 152.2, 142.7, 136.1, 130.2, 128.4, 128.2, 127.0, 124.9, 61.4, 61.0, 47.3, 22.0, 12.6; ^{11}B NMR (128 MHz, Acetonitrile-d_3) δ 10.1; HRMS (DART-TOF) calculated for $[C_{22}H_{21}BN_2O_6S+H]^+$ 453.1292, found 453.1295.

6-methyl-2-{17-phenyl-13-thia-11-azatetracyclo[8.7.0.0^{2,7}.0^{12,16}]heptadeca-1(10),2,4,6,11,14,16-heptaen-14-yl}-1,3,6,2-dioxazaborocane-4,8-dione (8e)

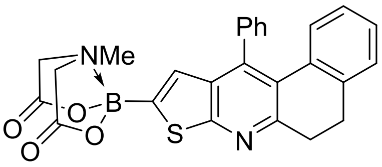

The title compound was obtained as pale yellow solid in 81% (32 mg) following the standard procedure. Reaction time: 16 h. TLC (EtOAc, R_f = 0.44); m.p > 350 °C; ^{1}H NMR (400 MHz, Acetonitrile-d_3) δ 8.45 – 8.37 (m, 1H), 7.65 – 7.50 (m, 3H), 7.48 – 7.25 (m, 5H), 7.10 (s, 1H), 4.12 (d, J = 17.2 Hz, 2H), 3.96 (d, J = 17.2 Hz, 2H), 2.88 (s, 4H), 2.73 (s, 3H); ^{13}C NMR (126 MHz, Acetonitrile-d_3) δ 167.9, 162.2, 150.6, 143.5, 139.0, 136.8, 134.8, 132.4, 129.3, 129.2, 128.5, 128.2, 127.7, 127.6, 127.0, 125.9, 125.3, 61.7, 47.6, 27.7, 25.3; ^{11}B NMR (128 MHz, Acetonitrile-d_3) δ 10.1; HRMS (DART-TOF) calculated for $[C_{26}H_{21}BN_2O_4S+H]^+$ 469.1393, found 469.1394.

Copies of ^{1}H NMR, ^{13}C NMR and ^{11}B NMR

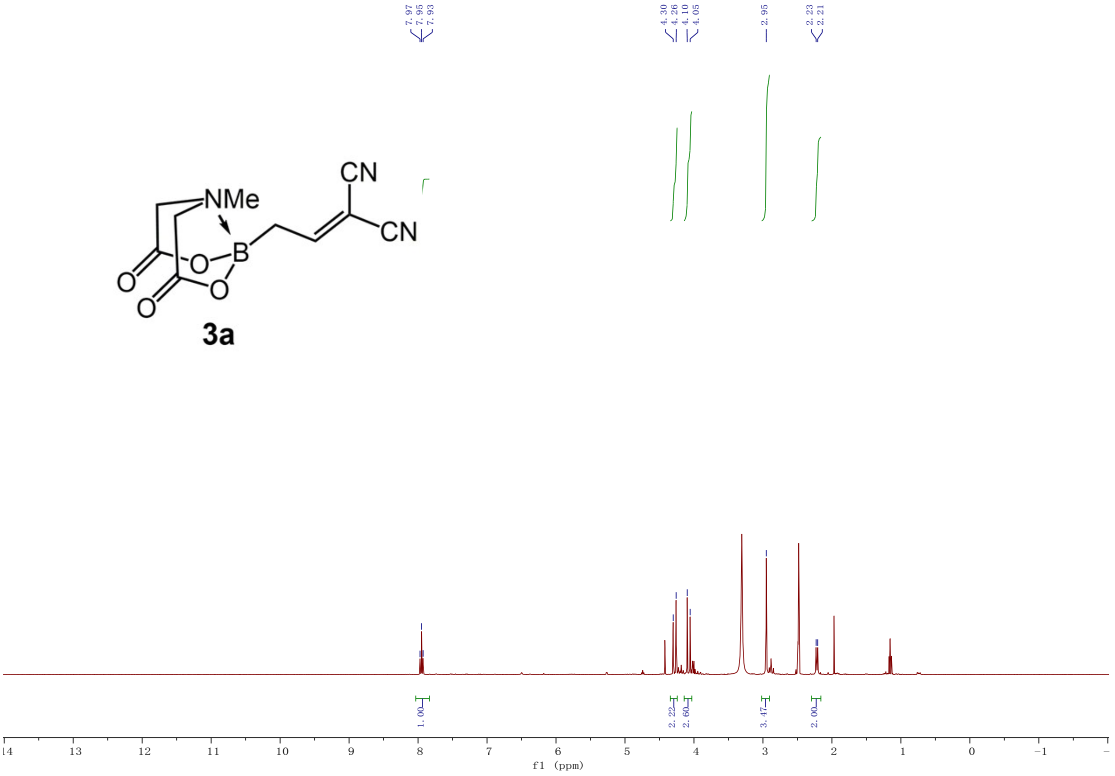

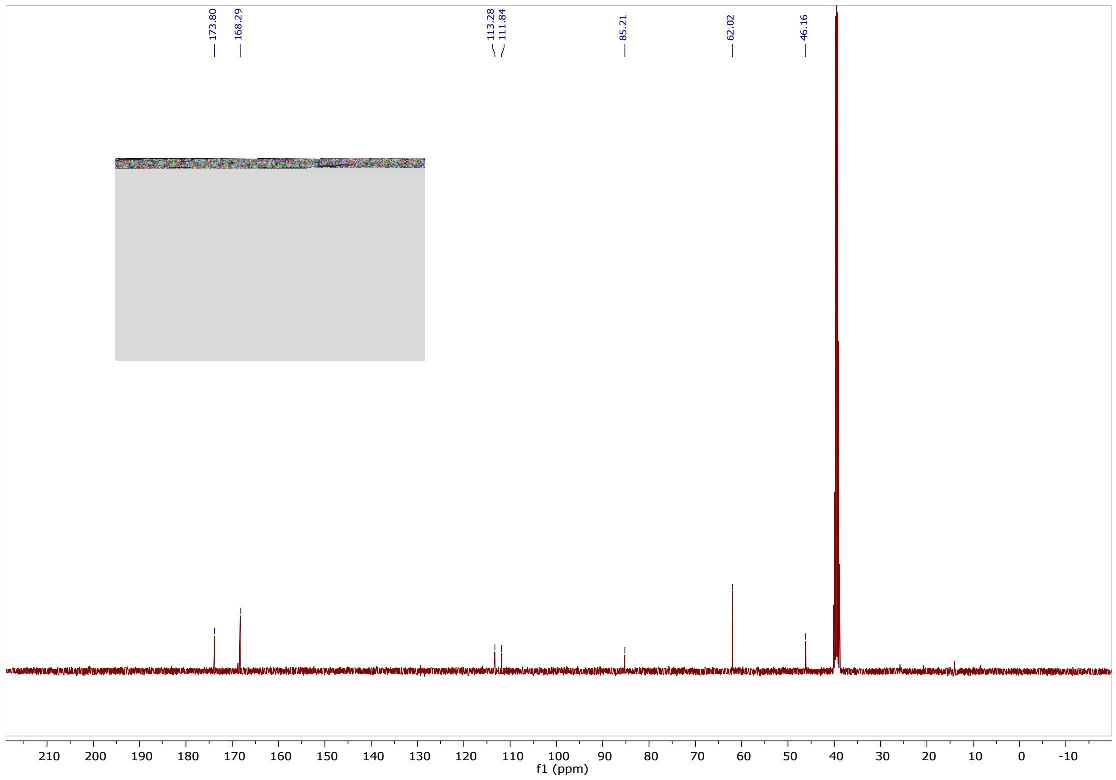
173.80
168.29
113.28
111.84
85.21
62.02
46.16
210 200 190 180 170 160 150 140 130 120 110 100 90 80 70 60 50 40 30 20 10 0 -10
f1 (ppm)

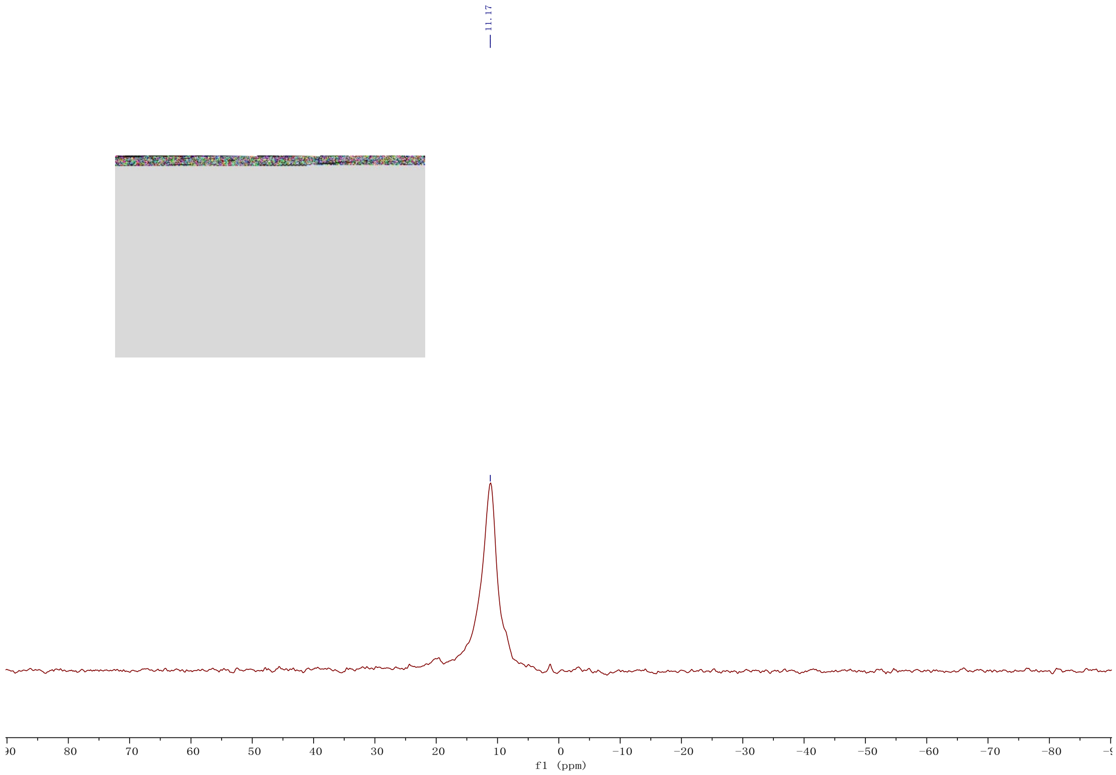
11.17
80 70 60 50 40 30 20 10 0 −10 −20 −30 −40 −50 −60 −70 −80
f1 (ppm)

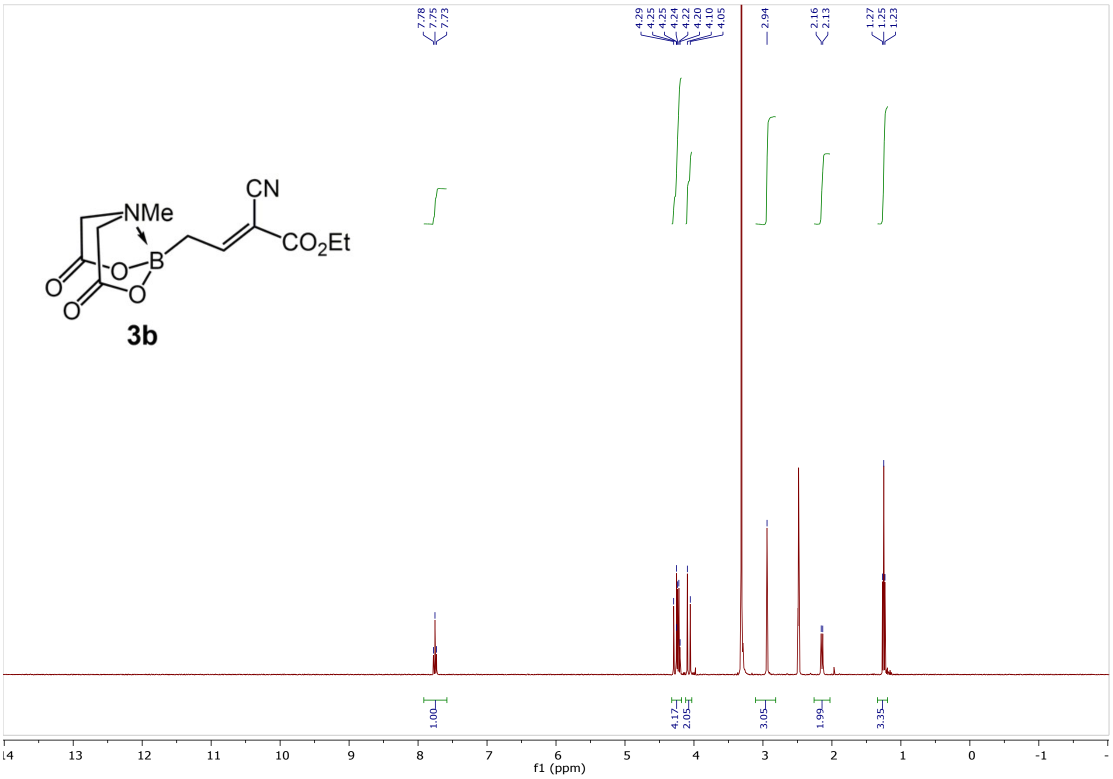

CN
NMe
CO2Et
3b
f1 (ppm)

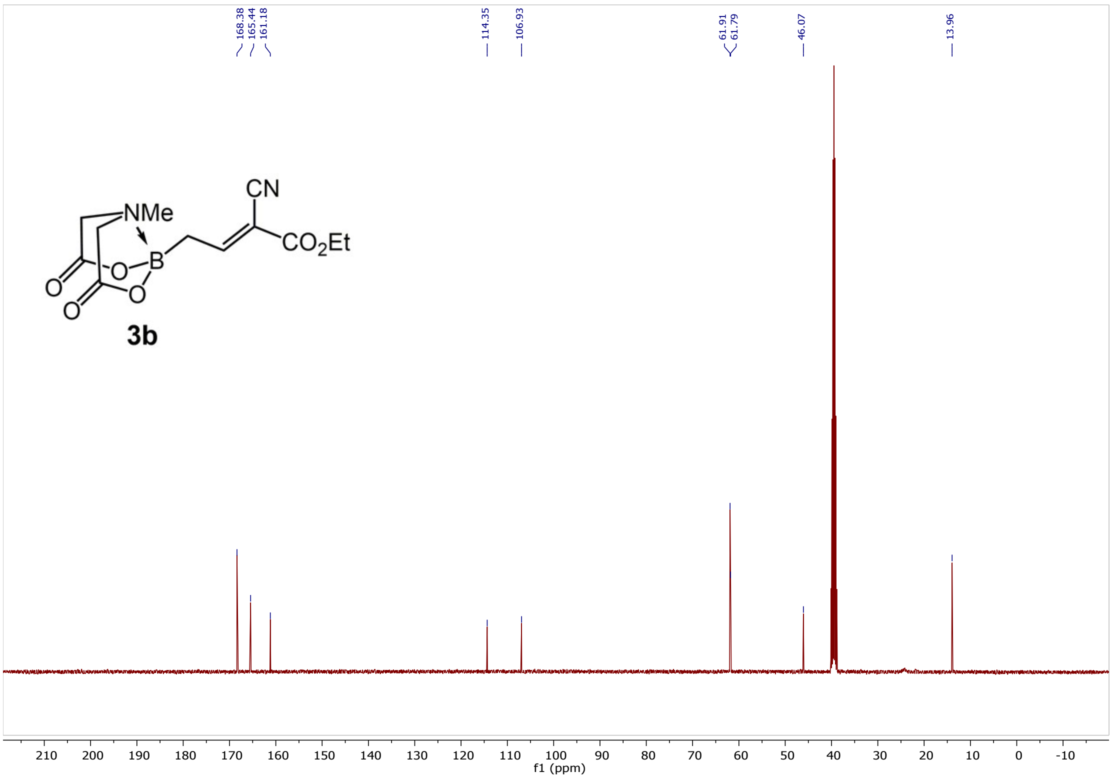

CN
NMe
CO2Et
3b
f1 (ppm)

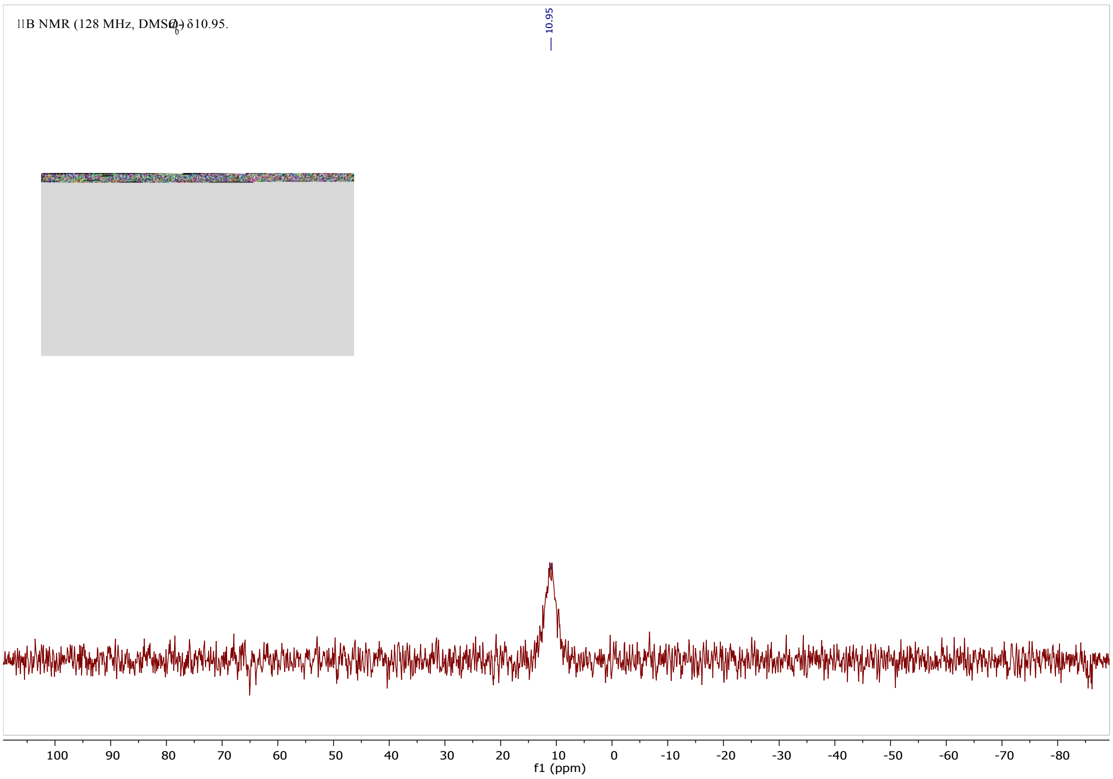
11B NMR (128 MHz, DMSO-d_6) δ 10.95.
10.95
f1 (ppm)

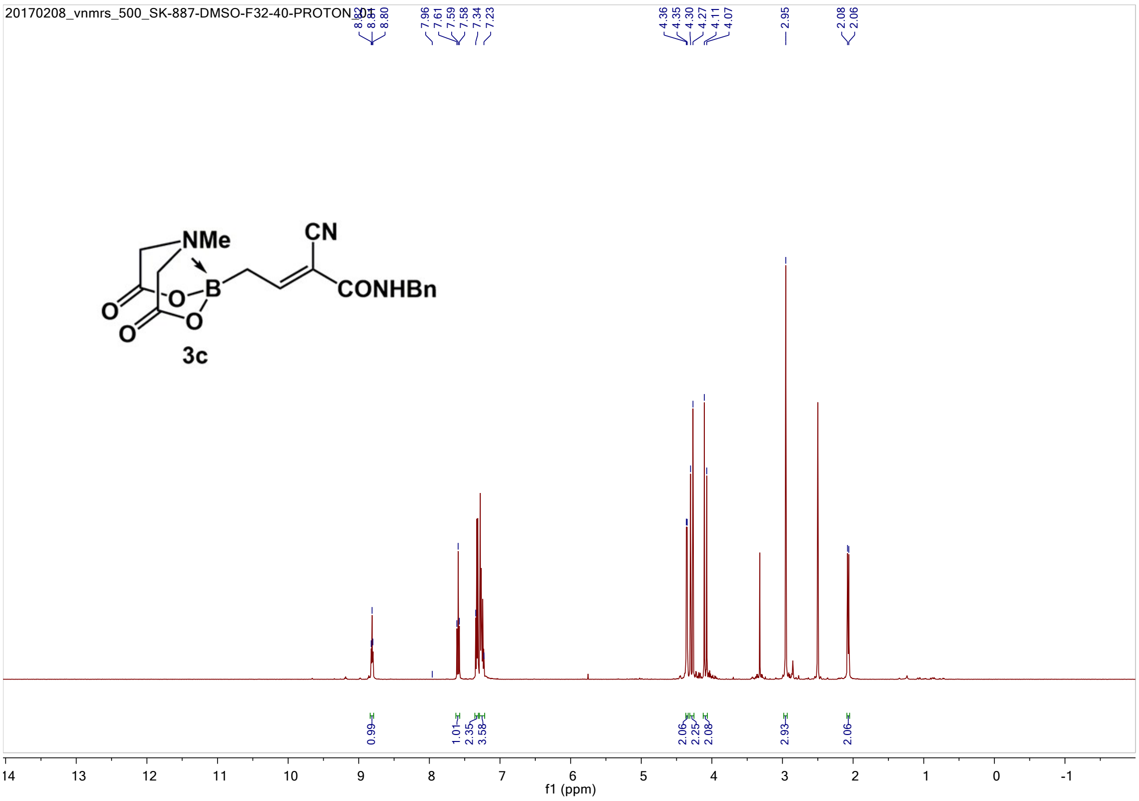
20170208_vnmrs_500_SK-887-DMSO-F32-40-PROTON_01
NMe
B
O
CN
CONHBn
3c
f1 (ppm)

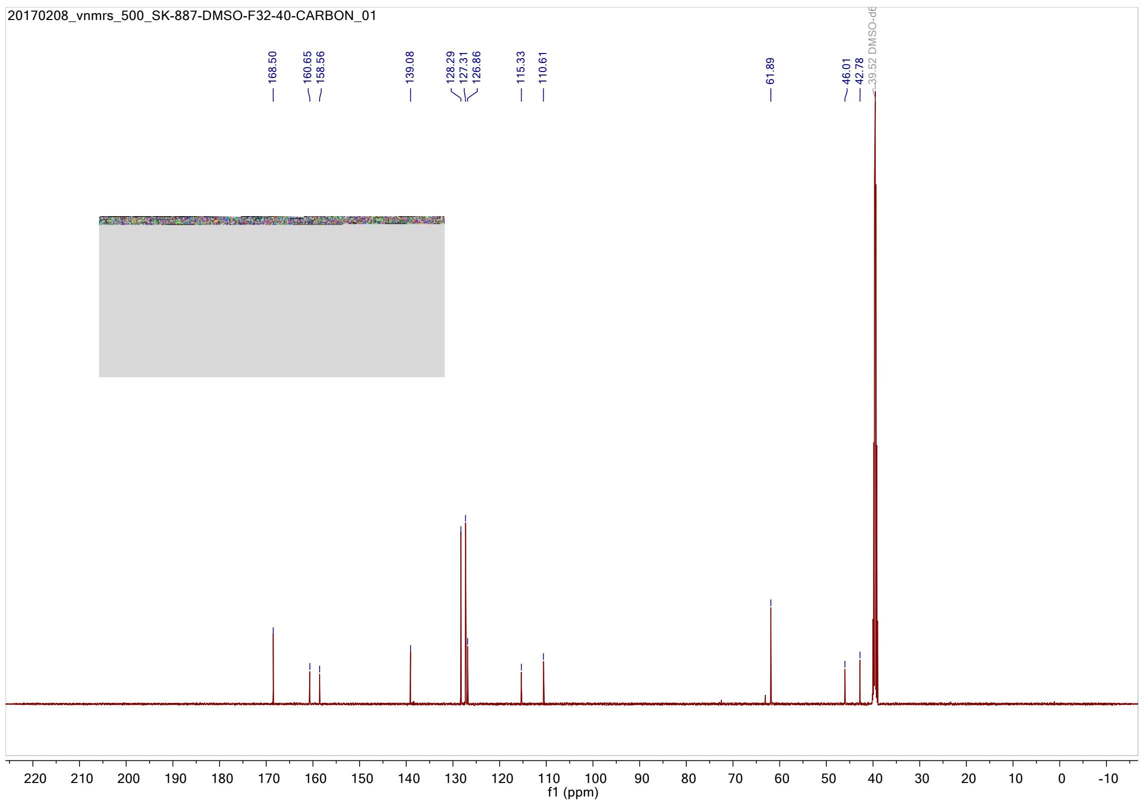
20170208_vnmrs_500_SK-887-DMSO-F32-40-CARBON_01
168.50
160.65
158.56
139.08
128.29
127.31
126.86
115.33
110.61
61.89
46.01
42.78
39.52 DMSO-d6
220 210 200 190 180 170 160 150 140 130 120 110 100 90 80 70 60 50 40 30 20 10 0 -10
f1 (ppm)

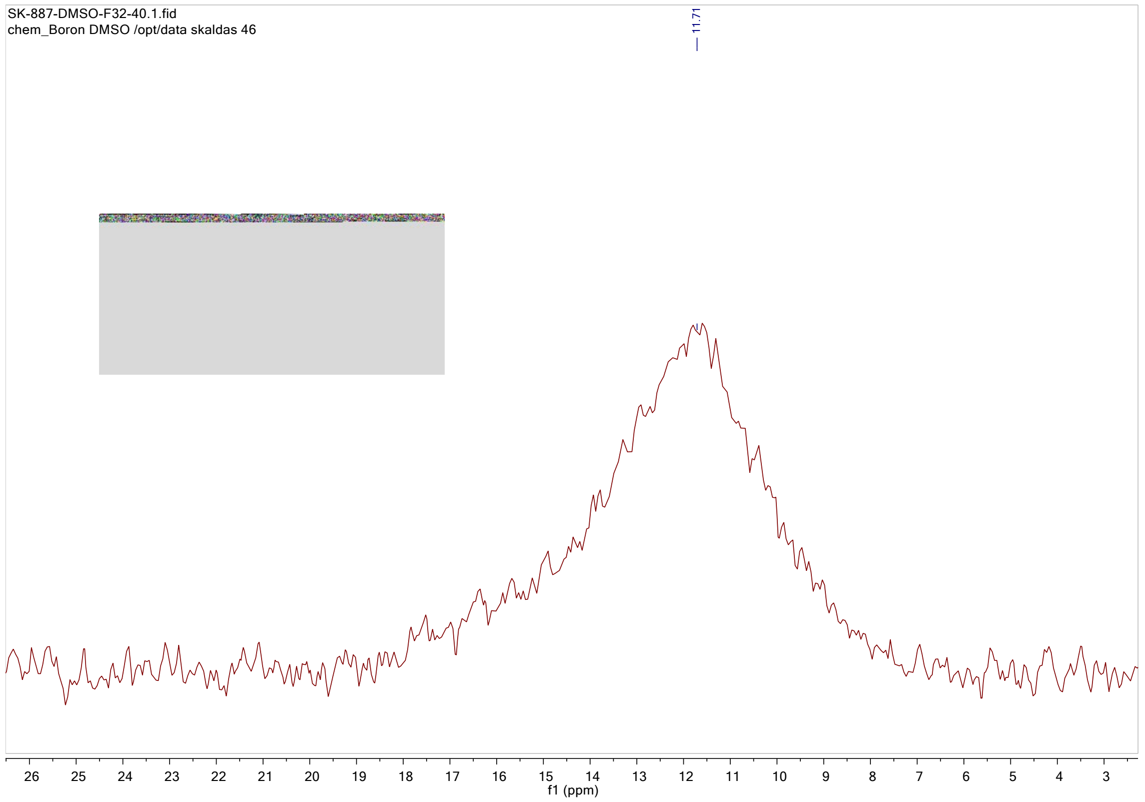
SK-887-DMSO-F32-40.1.fid
chem_Boron DMSO /opt/data skaldas 46
11.71
26 25 24 23 22 21 20 19 18 17 16 15 14 13 12 11 10 9 8 7 6 5 4 3
f1 (ppm)

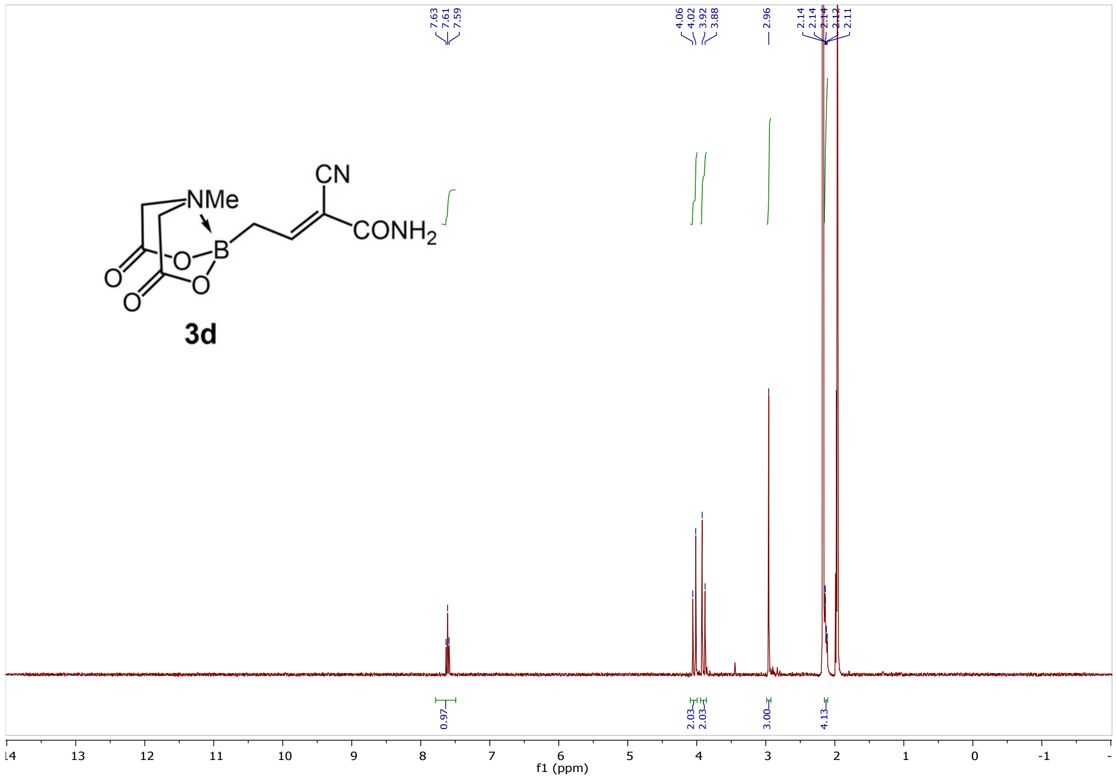
7.63
7.61
7.59
4.06
4.02
3.92
3.88
2.96
2.14
2.14
2.14
2.12
2.11
NMe
CN
CONH2
B
O
O
O
O
3d
0.97
2.03
2.03
3.00
4.13
f1 (ppm)

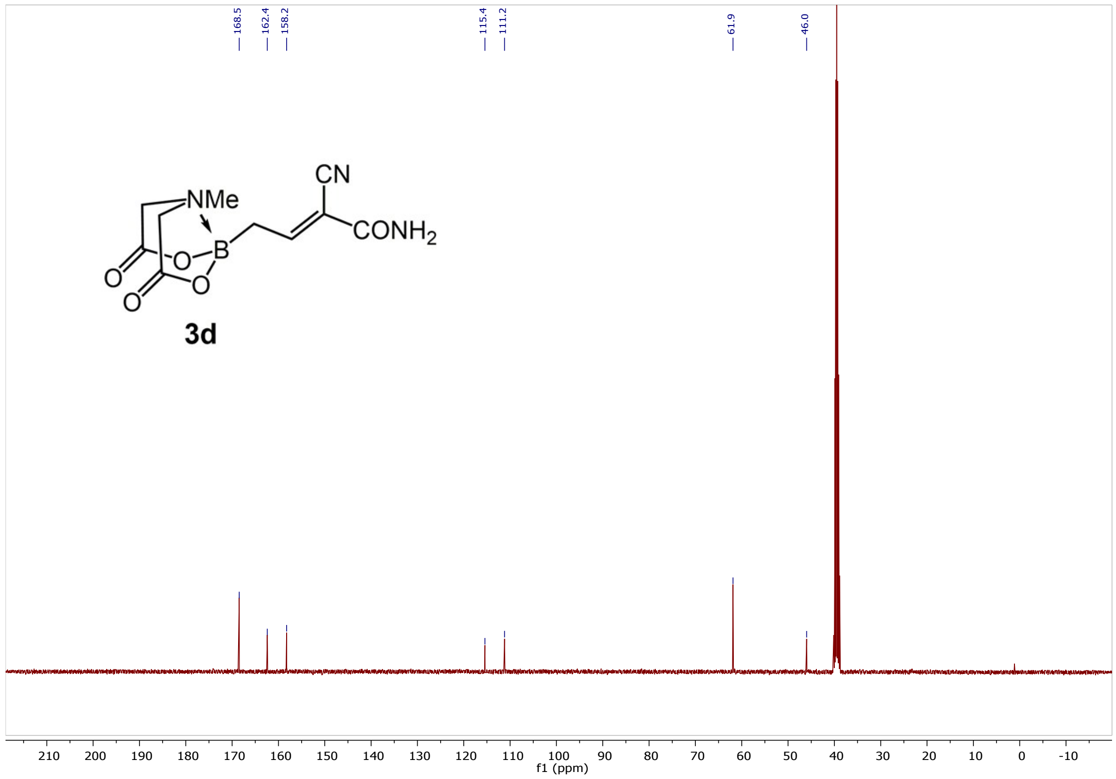
168.5
162.4
158.2
115.4
111.2
61.9
46.0
NMe
CN
CONH2
B
O
O
O
O
3d
f1 (ppm)

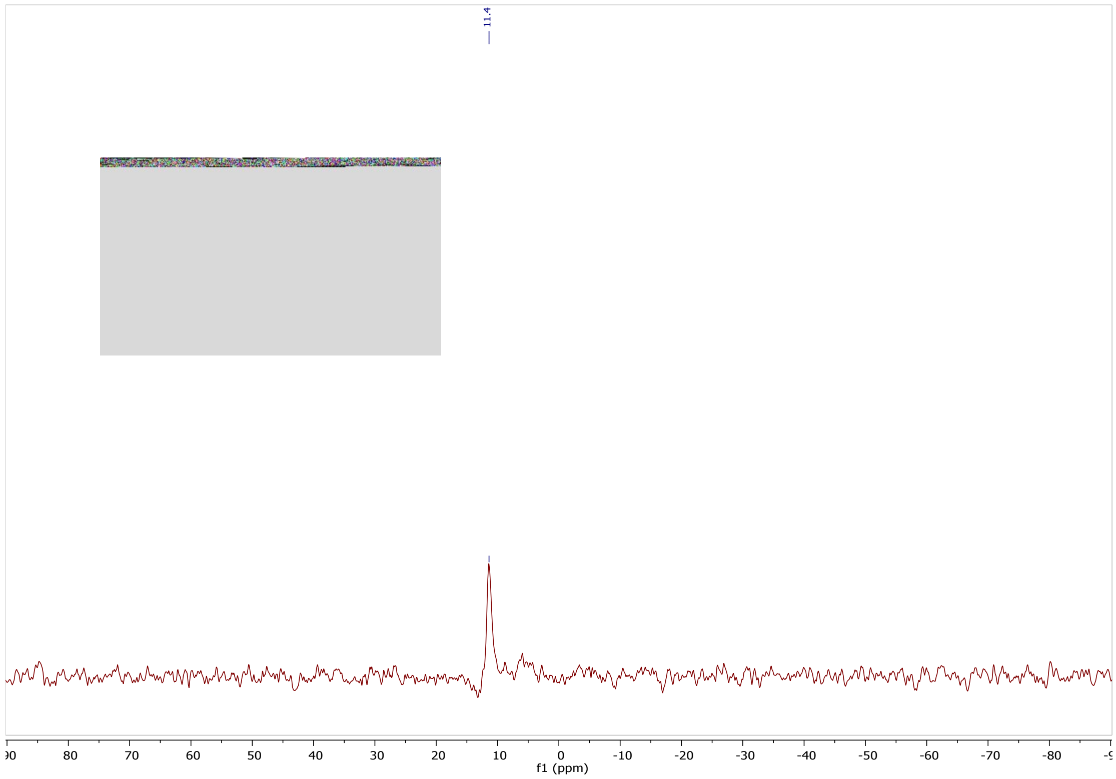
11.4
f1 (ppm)

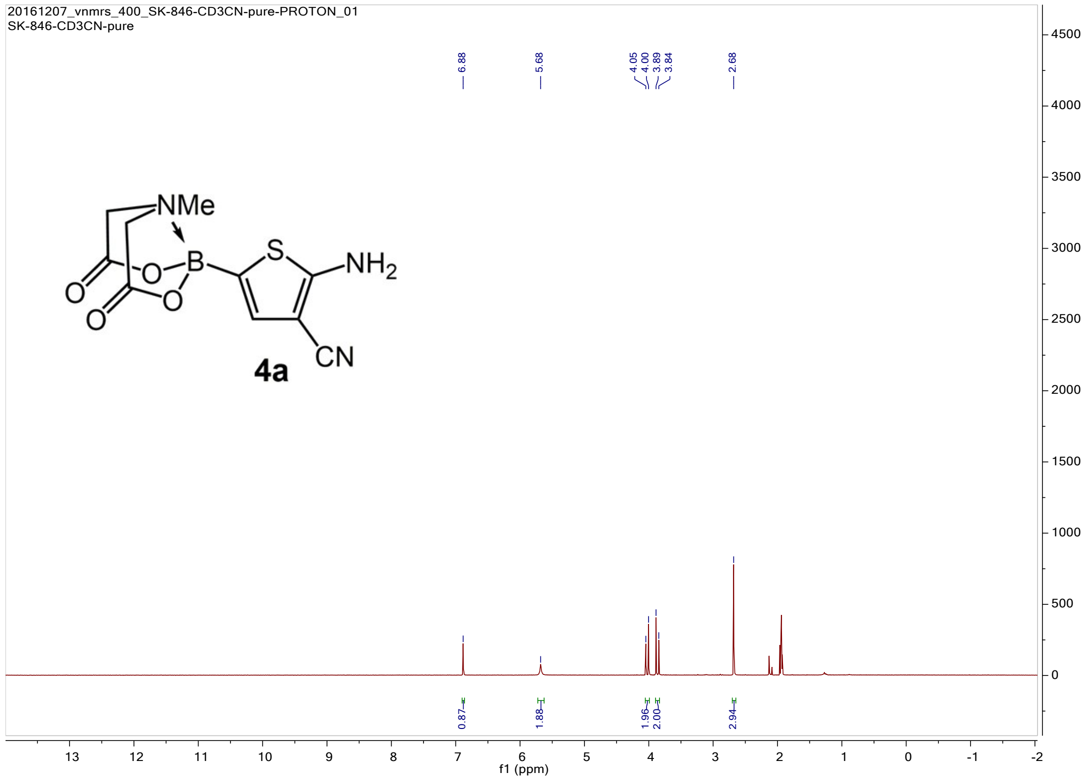
20161207_vnmrs_400_SK-846-CD3CN-pure-PROTON_01
SK-846-CD3CN-pure
6.88
5.68
4.05
4.00
3.89
3.84
2.68
NMe
B
O
O
O
O
S
NH2
CN
4a
0.87
1.88
1.96
2.00
2.94
f1 (ppm)

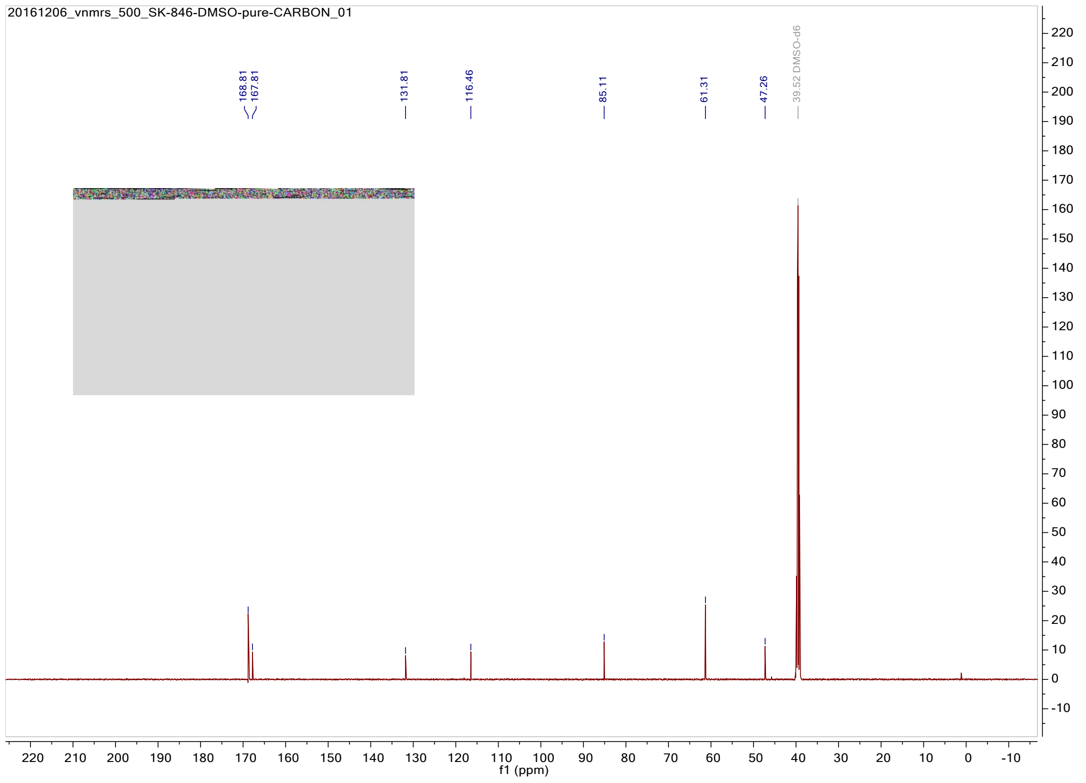
20161206_vnmrs_500_SK-846-DMSO-pure-CARBON_01
168.81
167.81
131.81
116.46
85.11
61.31
47.26
39.52 DMSO-d6
f1 (ppm)

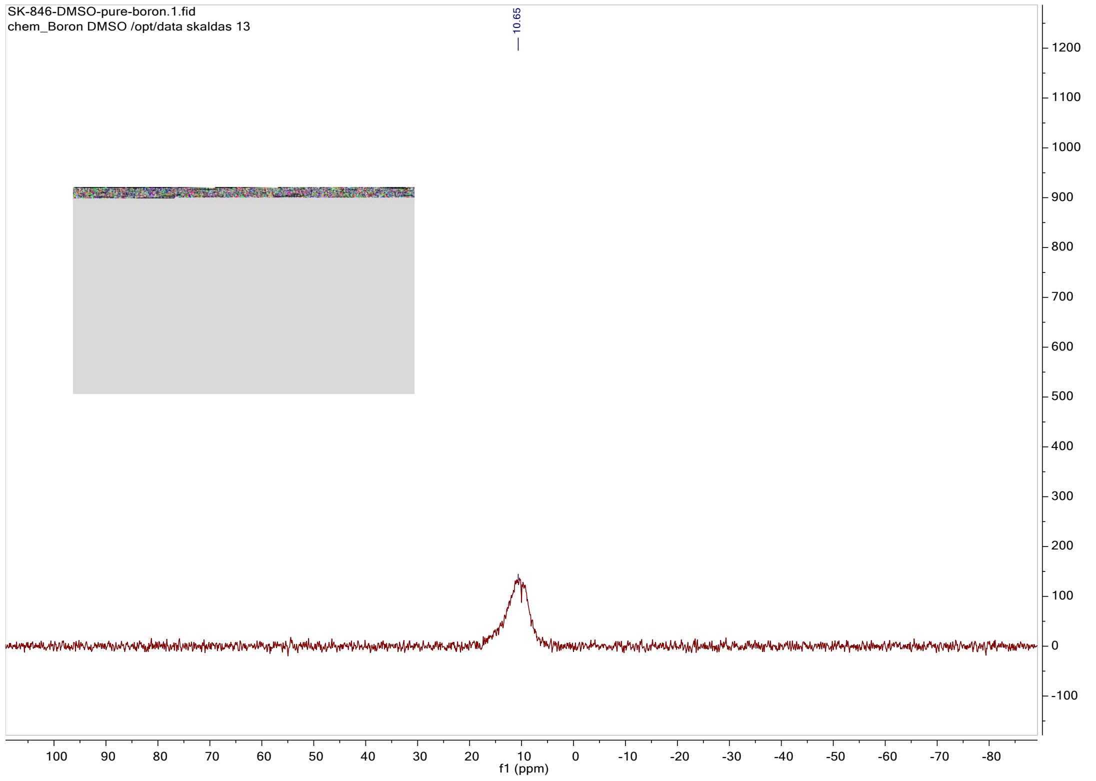
SK-846-DMSO-pure-boron.1.fid
chem_Boron DMSO /opt/data skaldas 13
10.65
f1 (ppm)

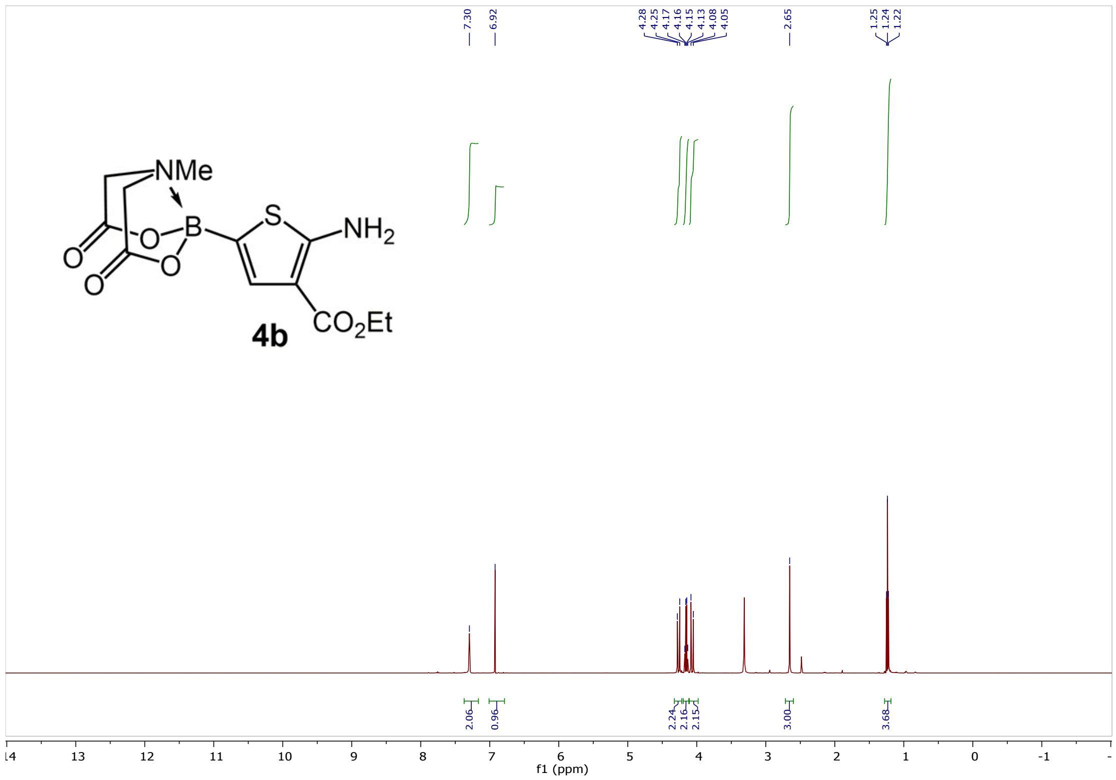
7.30
6.92
4.28
4.25
4.17
4.16
4.15
4.13
4.08
4.05
2.65
1.25
1.24
1.22
NMe
B
O
O
S
NH2
CO2Et
4b
2.06
0.96
2.24
2.16
2.15
3.00
3.68
f1 (ppm)

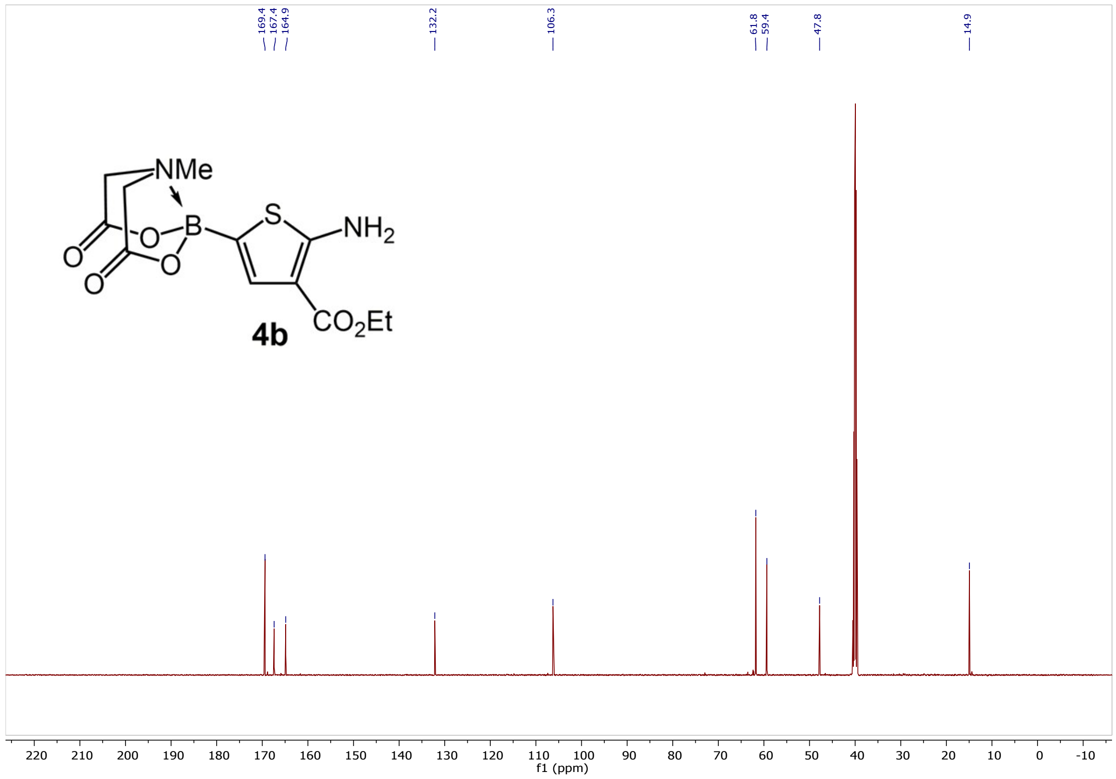
169.4
167.4
164.9
132.2
106.3
61.8
59.4
47.8
14.9
NMe
B
O
O
S
NH2
CO2Et
4b
f1 (ppm)

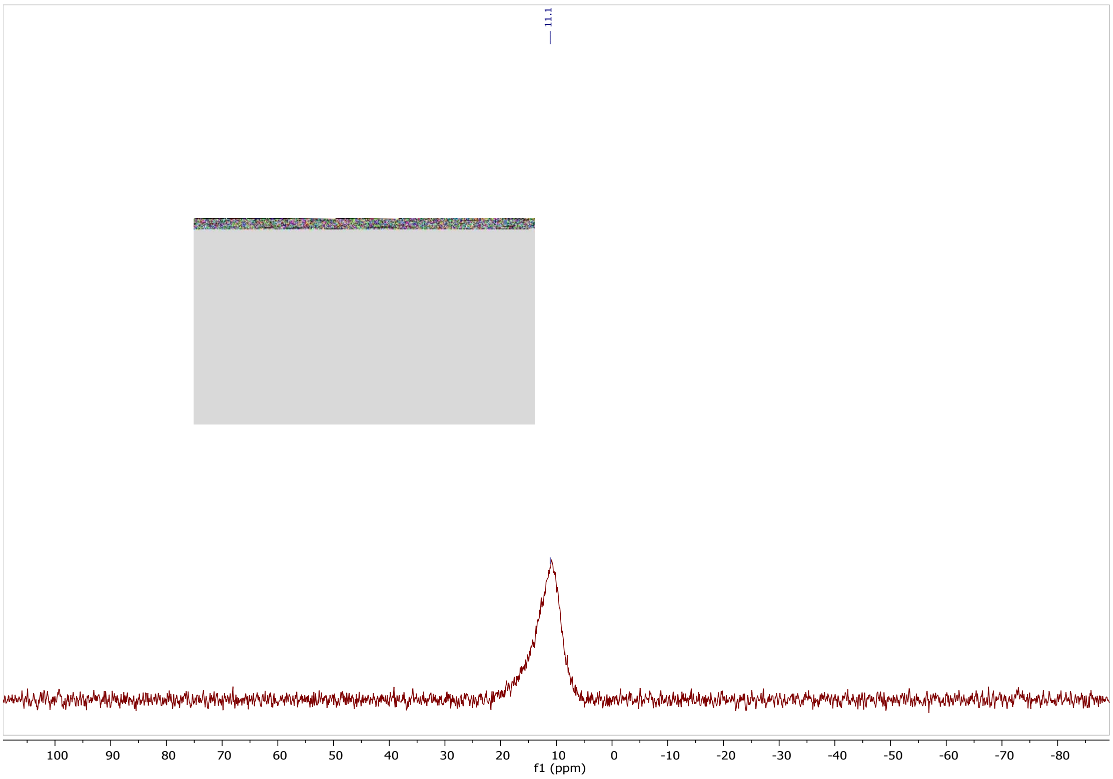
11.1
f1 (ppm)

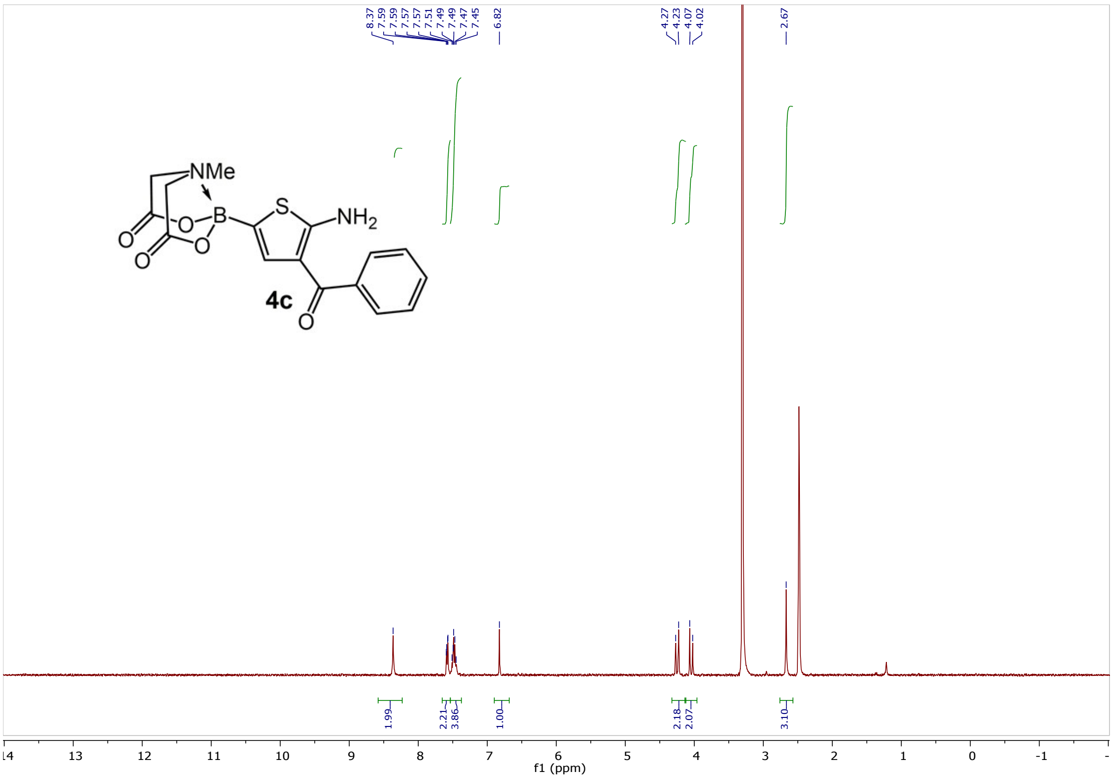
8.37 7.59 7.59 7.57 7.57 7.51 7.49 7.49 7.47 7.45
6.82
4.27 4.23 4.07 4.02
2.67
NMe
S
NH2
O
B
4c
1.99 2.21 3.86 1.00 2.18 2.07 3.10
f1 (ppm)

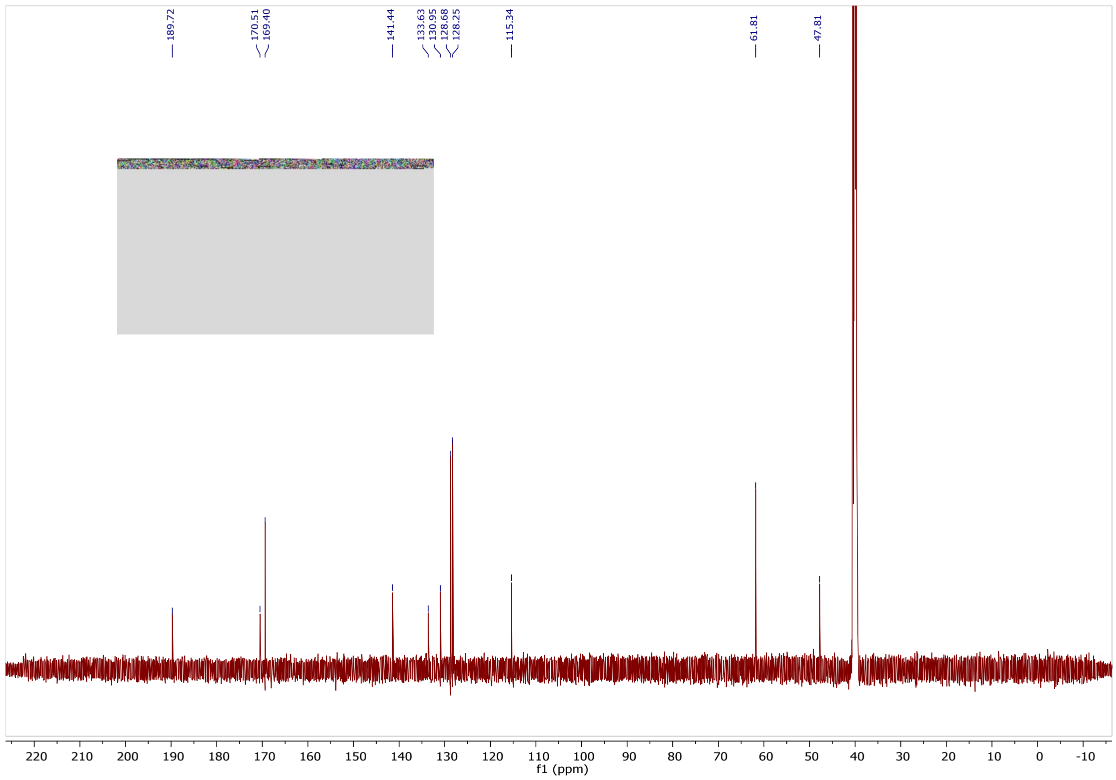
189.72
170.51
169.40
141.44
133.63
130.95
128.68
128.25
115.34
61.81
47.81
f1 (ppm)

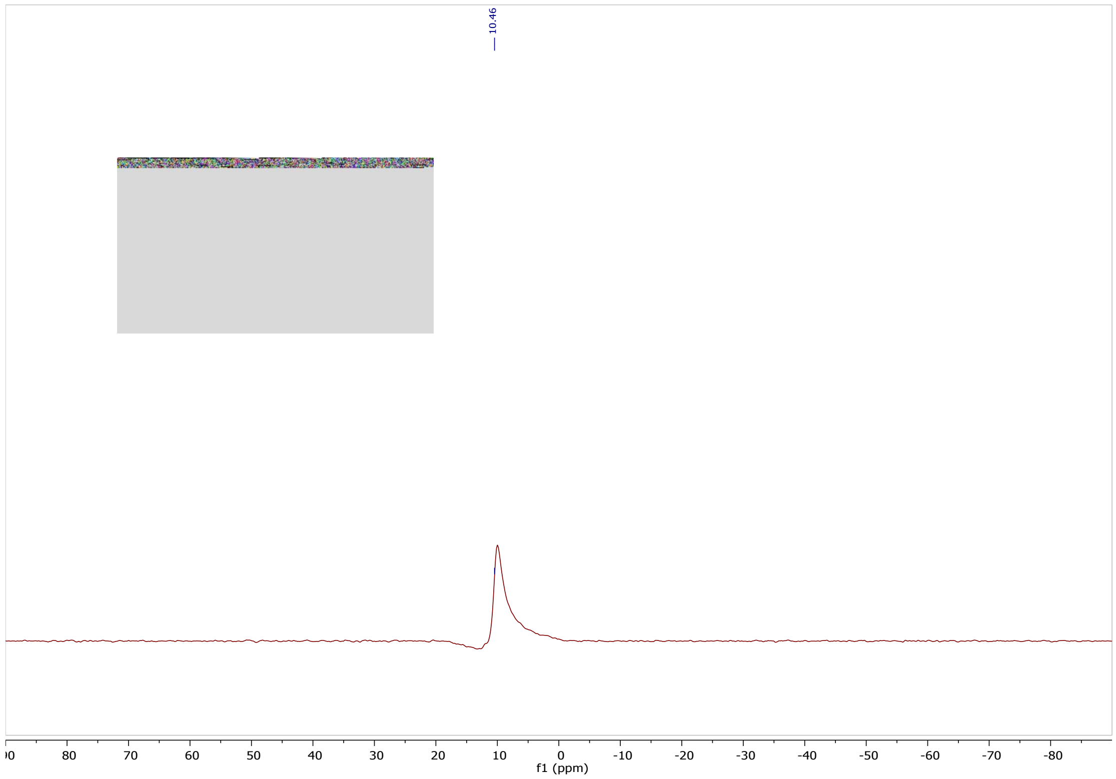
10.46
f1 (ppm)

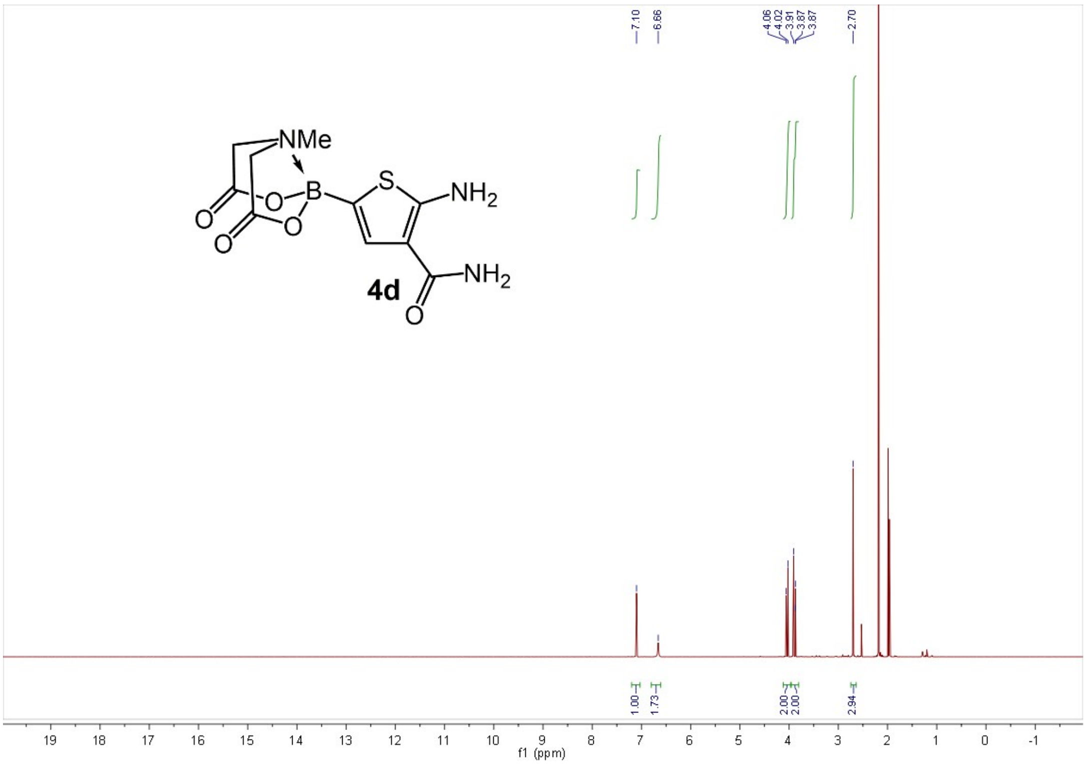
7.10
6.66
4.06
4.02
3.91
3.87
3.87
2.70
NMe
S
NH2
O
O
B
O
O
4d
NH2
O
1.00
1.73
2.00
2.00
2.94
19 18 17 16 15 14 13 12 11 10 9 8 7 6 5 4 3 2 1 0 -1
f1 (ppm)

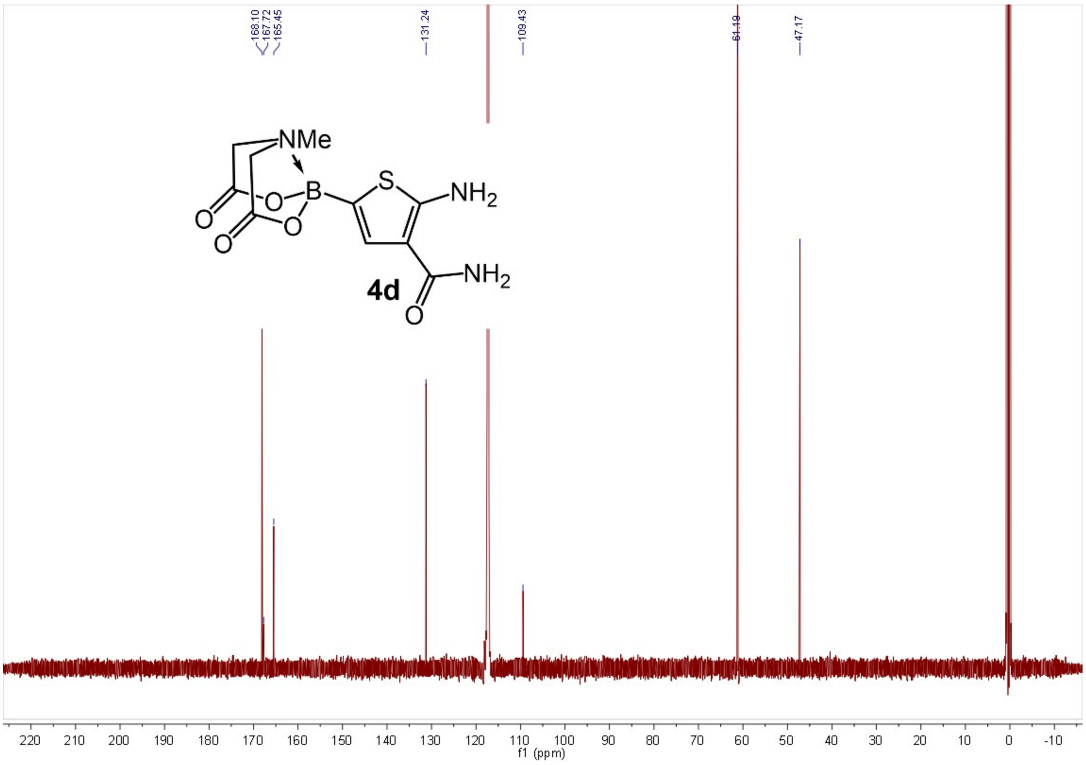
168.10
167.72
165.45
131.24
109.43
61.19
47.17
NMe
S
NH2
O
O
B
O
O
4d
NH2
O
220 210 200 190 180 170 160 150 140 130 120 110 100 90 80 70 60 50 40 30 20 10 0 -10
f1 (ppm)

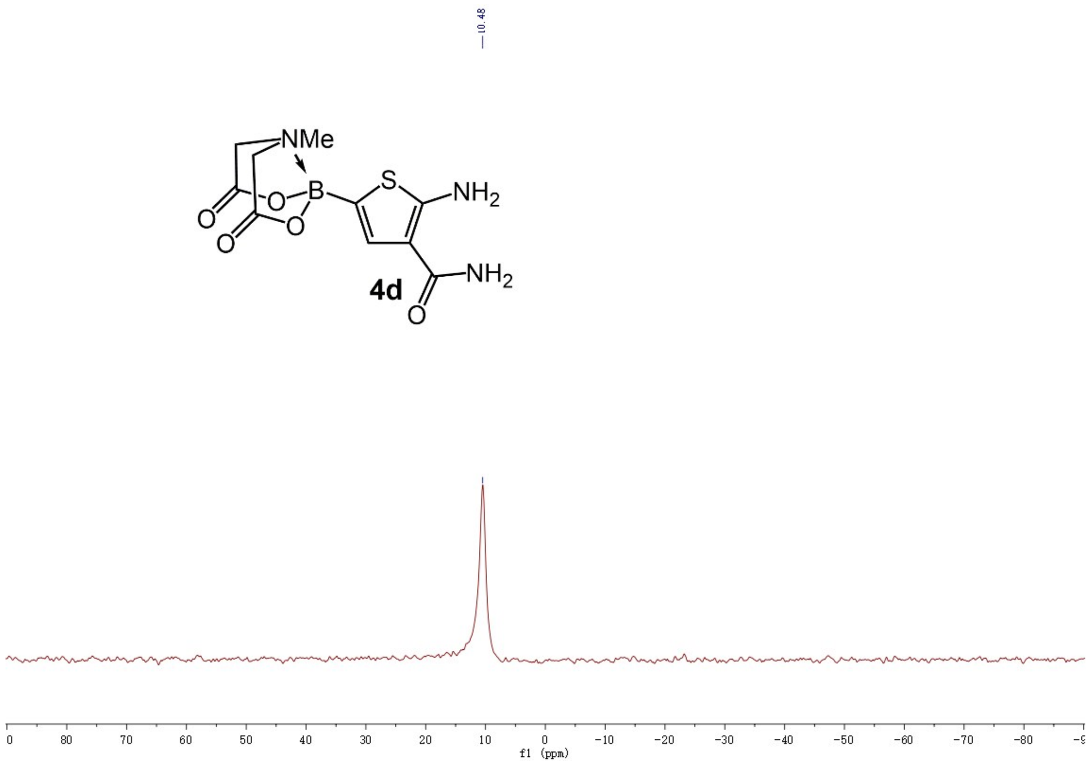
10.48
NMe
B
O
O
O
O
S
NH2
NH2
O
4d
f1 (ppm)

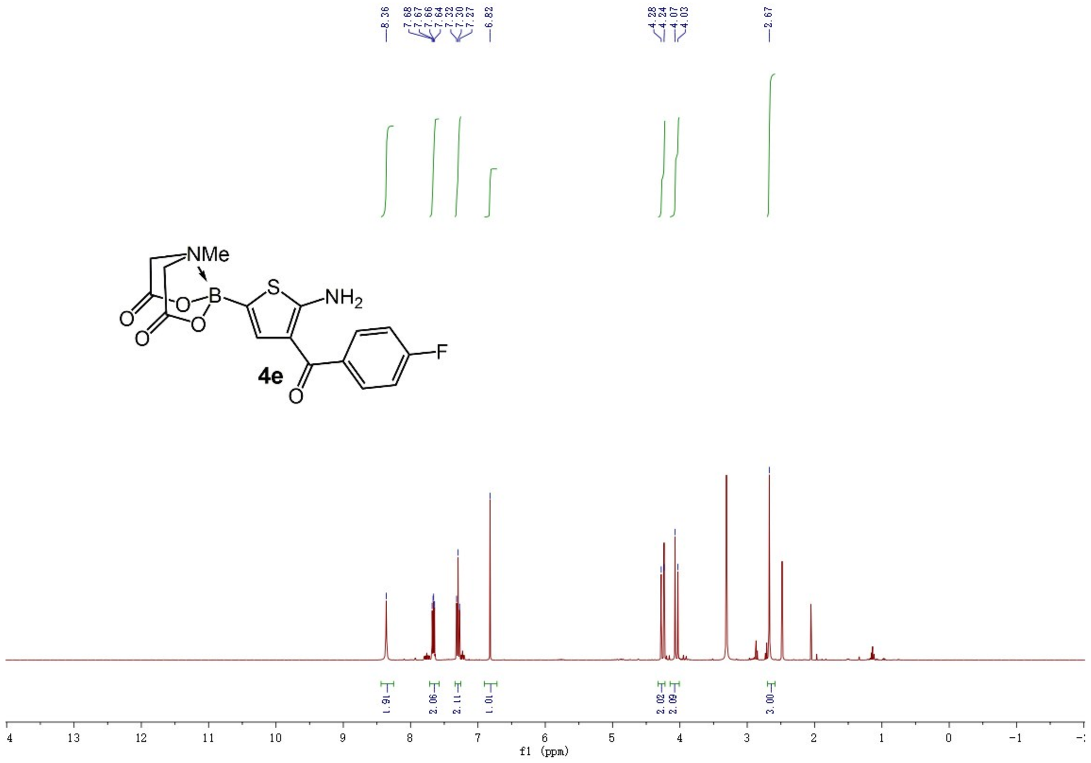
8.36
7.68
7.67
7.66
7.64
7.32
7.30
7.27
6.82
4.28
4.24
4.07
4.03
2.67
NMe
B
S
NH2
F
4e
1.91
2.06
2.11
1.01
2.02
2.09
3.00
f1 (ppm)

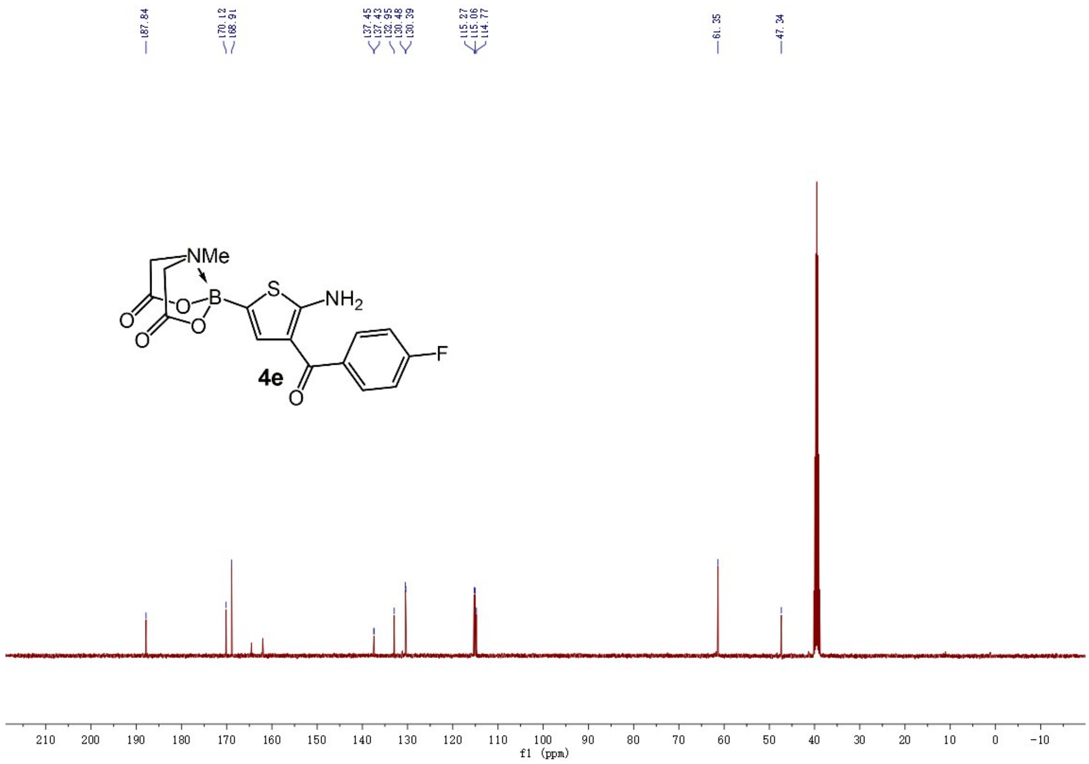

187.84
170.12
168.91
137.45
137.43
132.95
130.48
130.39
115.27
115.06
114.77
61.35
47.34
NMe
B
O
S
NH2
F
4e
f1 (ppm)

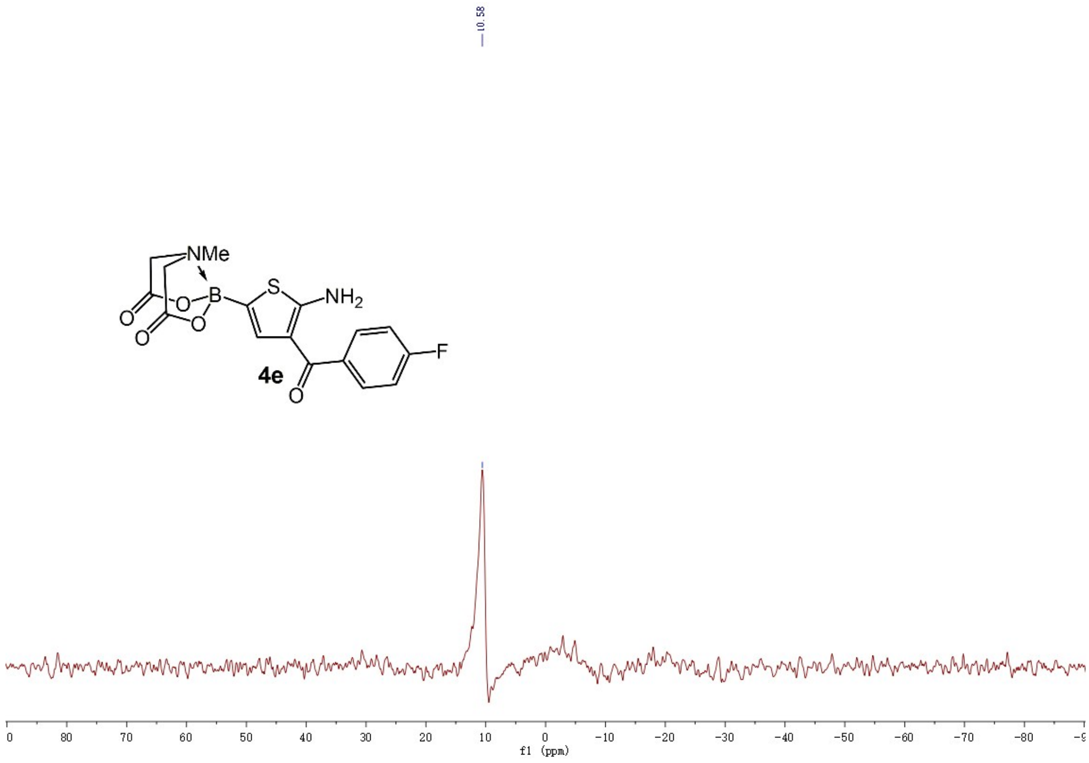

10.58
NMe
B
O
S
NH2
F
4e
f1 (ppm)

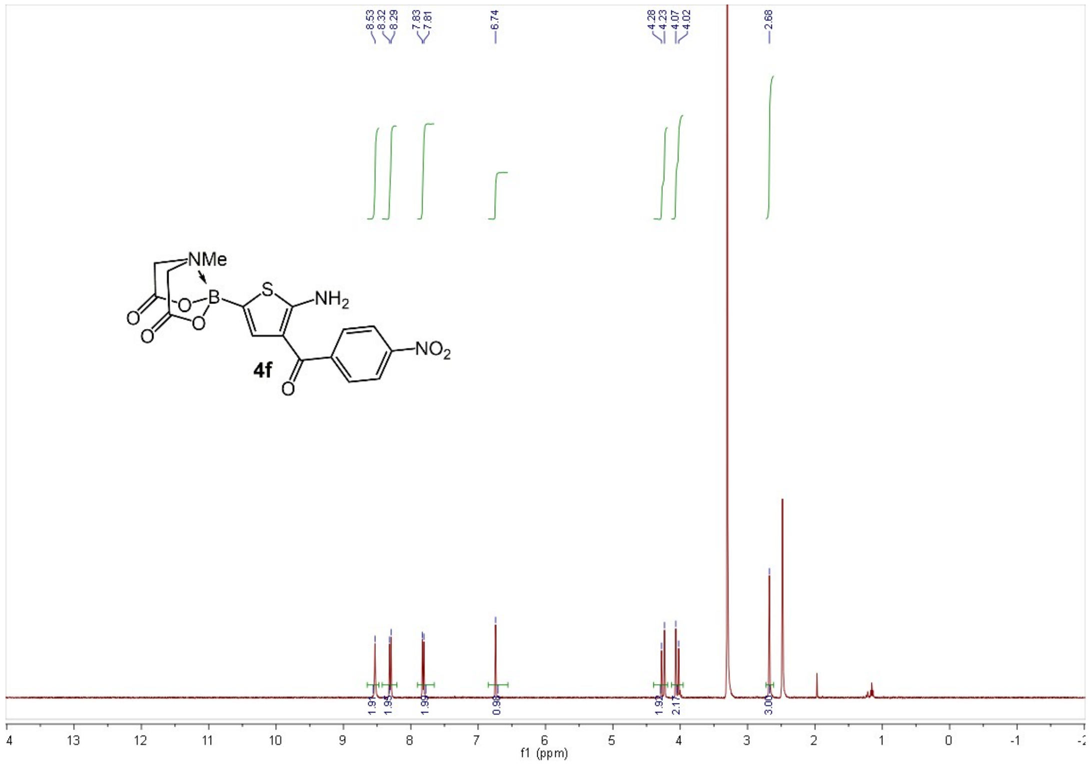
8.53
8.32
8.29
7.83
7.81
6.74
4.28
4.23
4.07
4.02
2.68
NMe
B
O
S
NH2
NO2
4f
f1 (ppm)

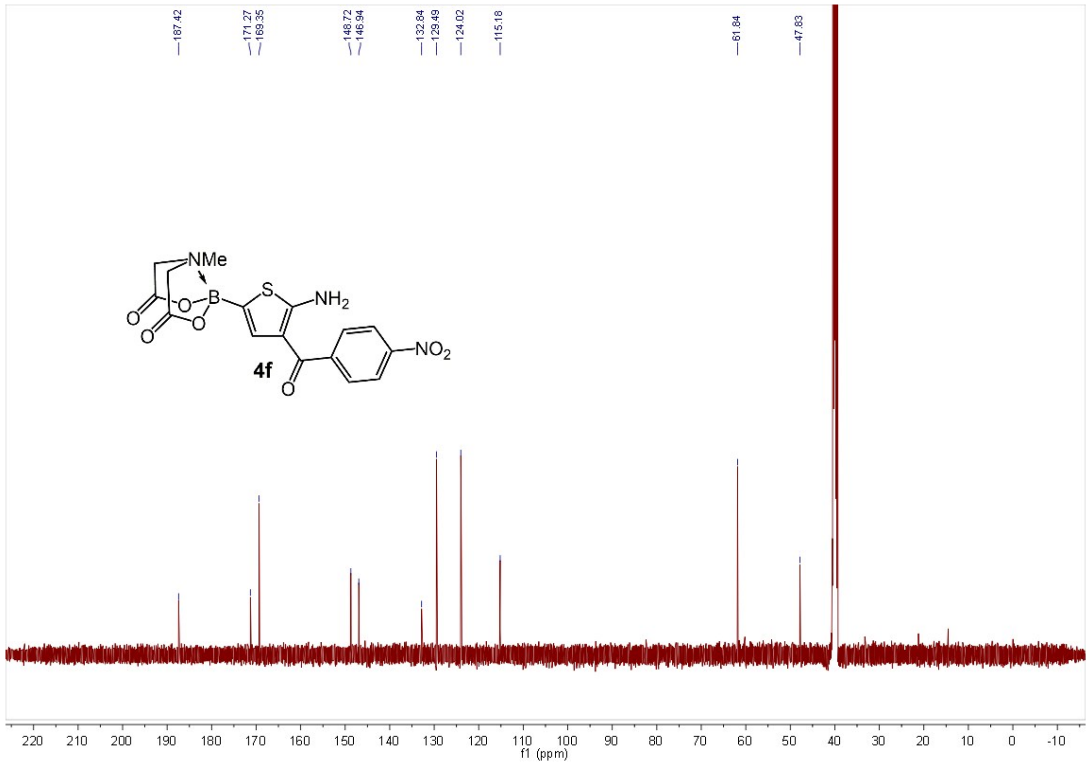
187.42
171.27
169.35
148.72
146.94
132.84
129.49
124.02
115.18
61.84
47.83
NMe
B
O
S
NH2
NO2
4f
f1 (ppm)

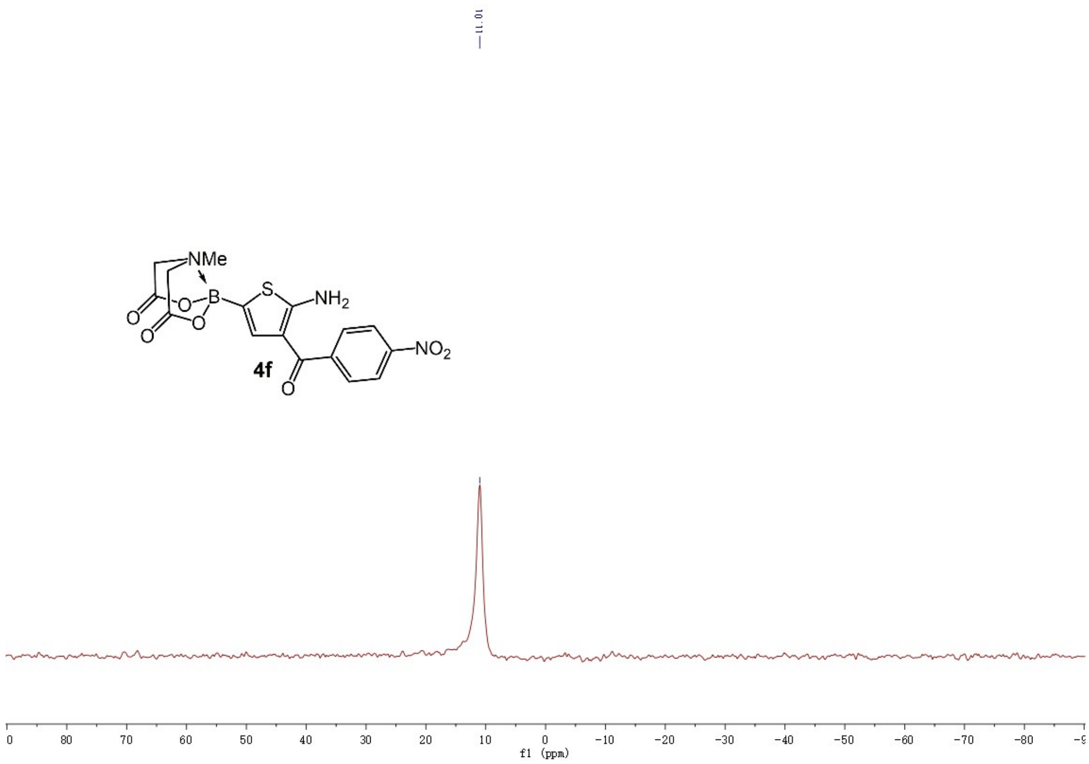

11.01
NMe
B
O
S
NH2
NO2
4f
f1 (ppm)

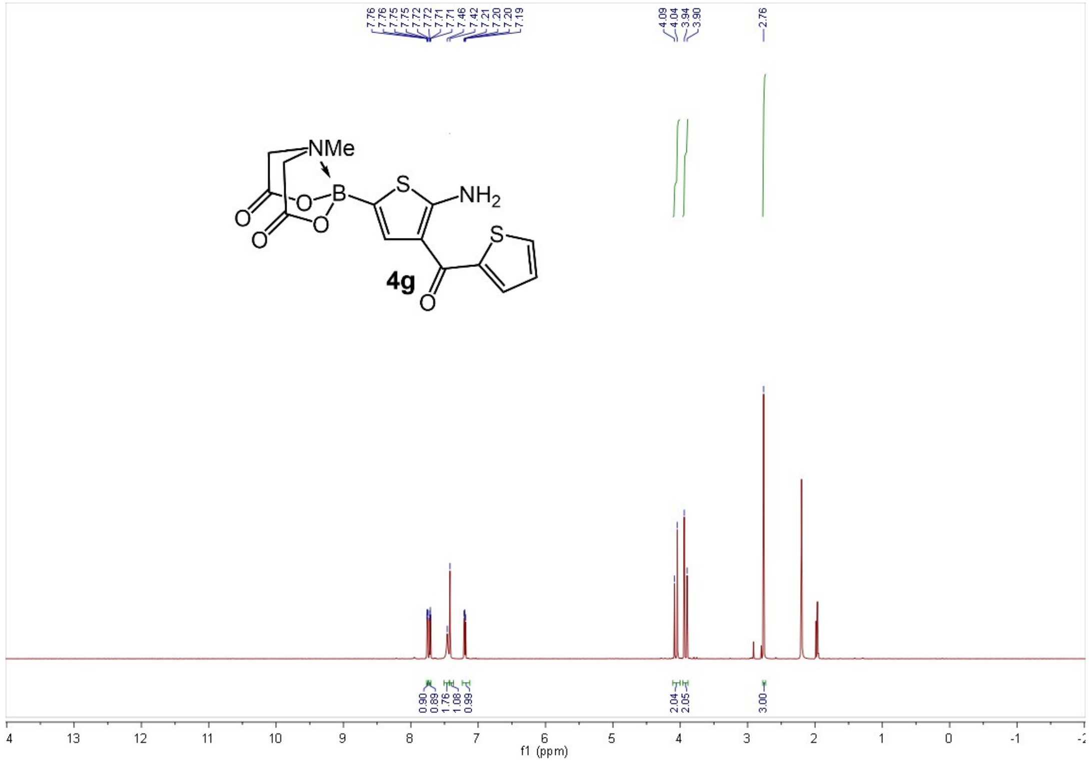

7.76 7.76 7.75 7.75 7.72 7.72 7.71 7.71 7.46 7.42 7.21 7.20 7.20 7.19
4.09 4.04 3.94 3.90
2.76
NMe
B
O
S
NH2
4g
0.90 0.89 1.76 1.08 0.99
2.04 2.05
3.00
f1 (ppm)

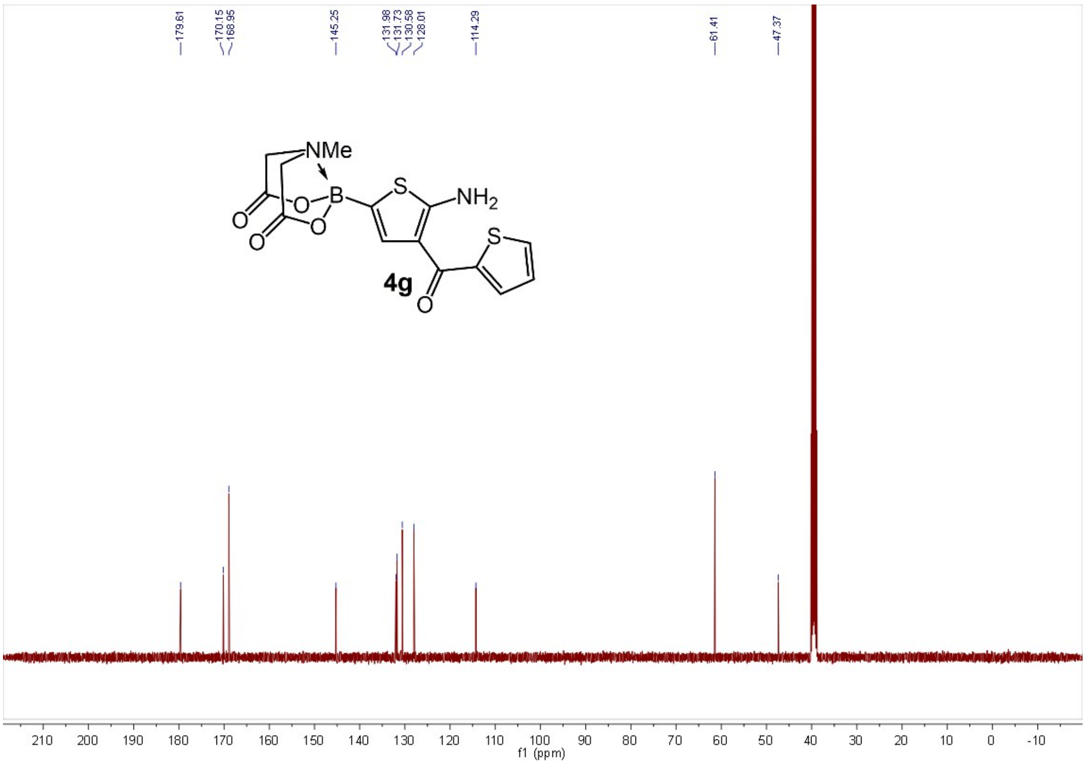
179.61
170.15
168.95
145.25
131.98
131.73
130.58
128.01
114.29
61.41
47.37
NMe
B
O
S
NH_2
4g
210 200 190 180 170 160 150 140 130 120 110 100 90 80 70 60 50 40 30 20 10 0 -10
f1 (ppm)

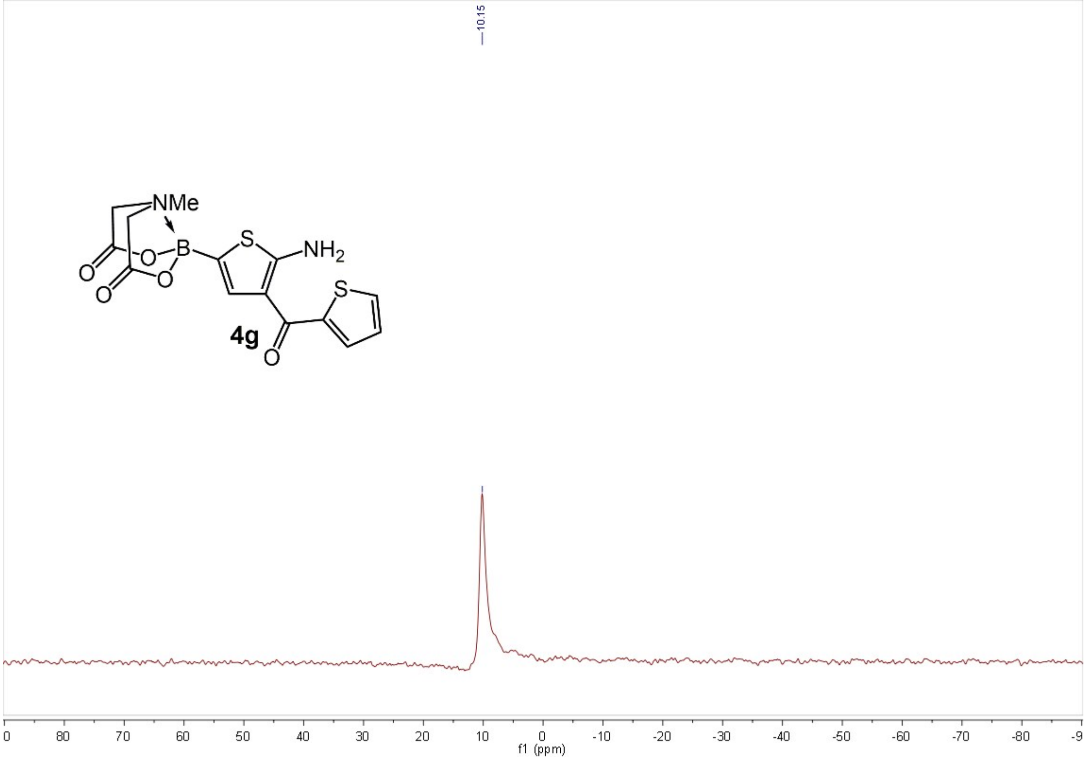
10.15
NMe
B
O
S
NH_2
4g
0 80 70 60 50 40 30 20 10 0 -10 -20 -30 -40 -50 -60 -70 -80 -9
f1 (ppm)

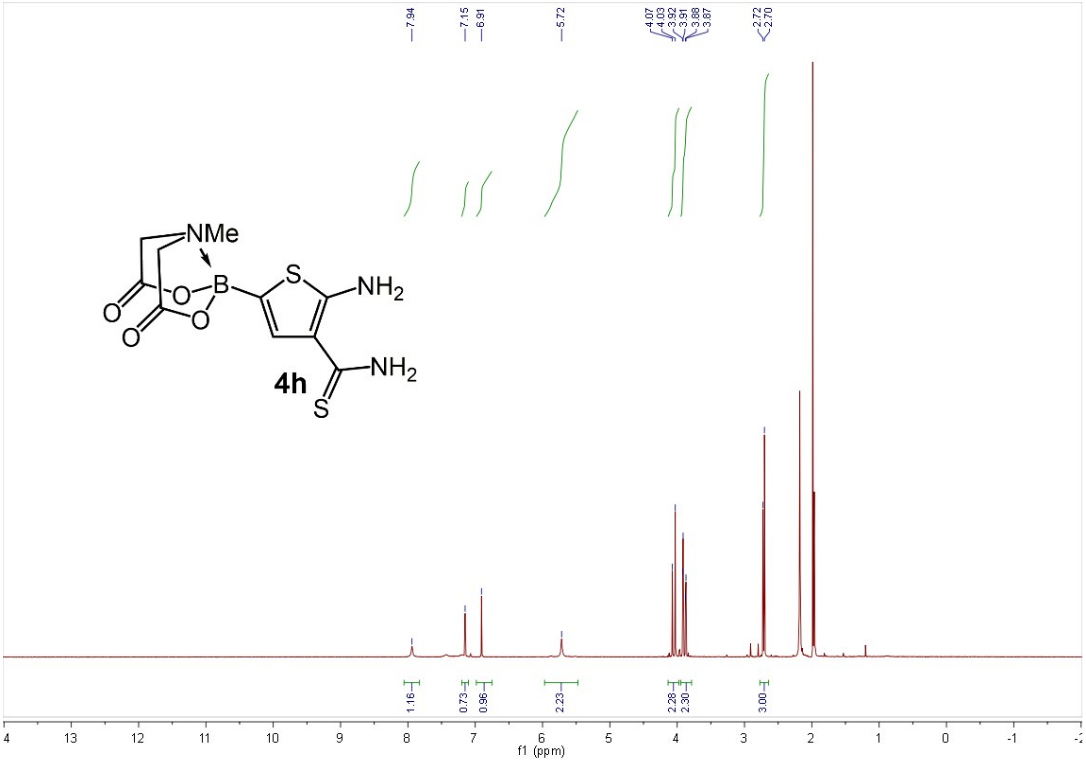
7.94
7.15
6.91
5.72
4.07
4.03
3.92
3.91
3.88
3.87
2.72
2.70
NMe
B
O
S
NH2
4h
S
NH2
1.16
0.73
0.96
2.23
2.28
2.30
3.00
f1 (ppm)

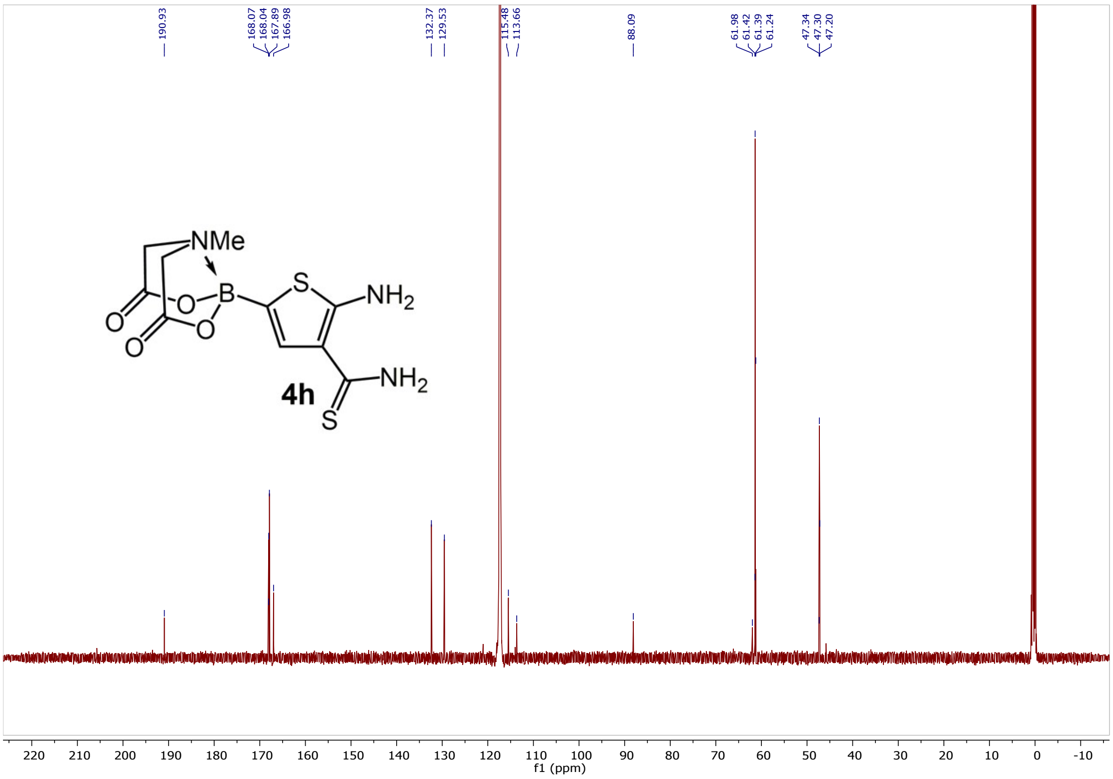
190.93
168.07
168.04
167.89
166.98
132.37
129.53
115.48
113.66
88.09
61.98
61.42
61.39
61.24
47.34
47.30
47.20
NMe
B
O
S
NH2
4h
S
NH2
f1 (ppm)

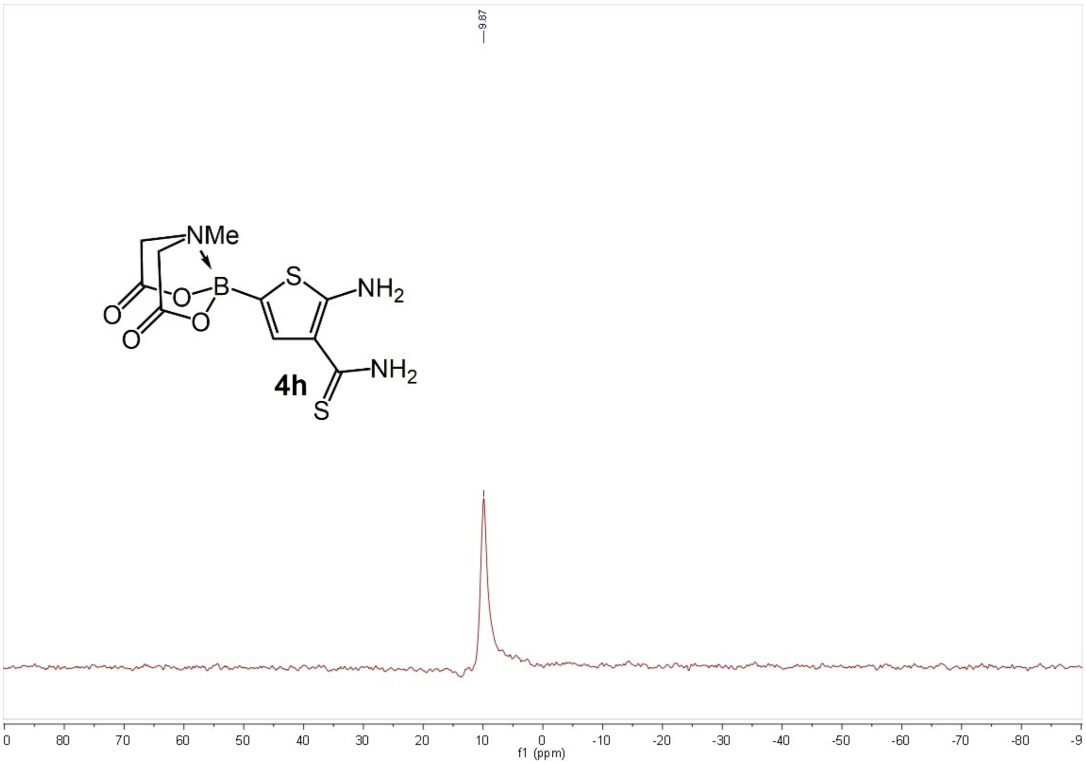

9.87
NMe
B
O
S
NH2
NH2
4h
f1 (ppm)

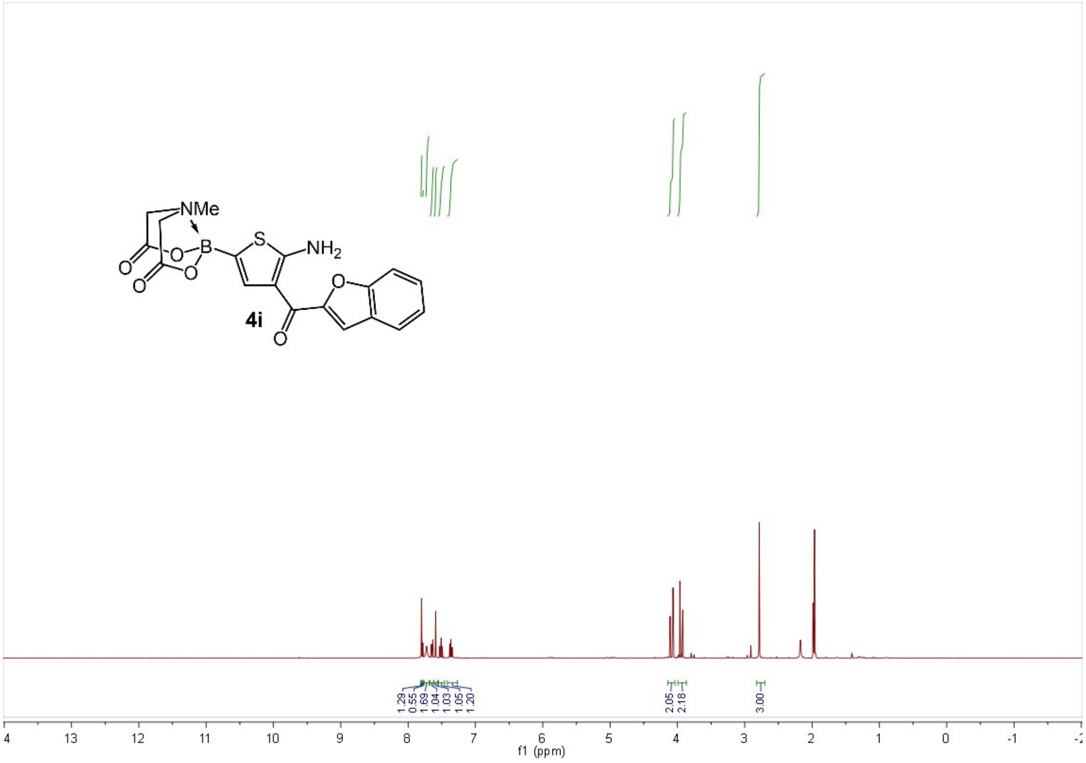

NMe
B
O
S
NH2
4i
1.29
0.55
1.69
1.04
1.03
1.05
1.20
2.05
2.18
3.00
f1 (ppm)

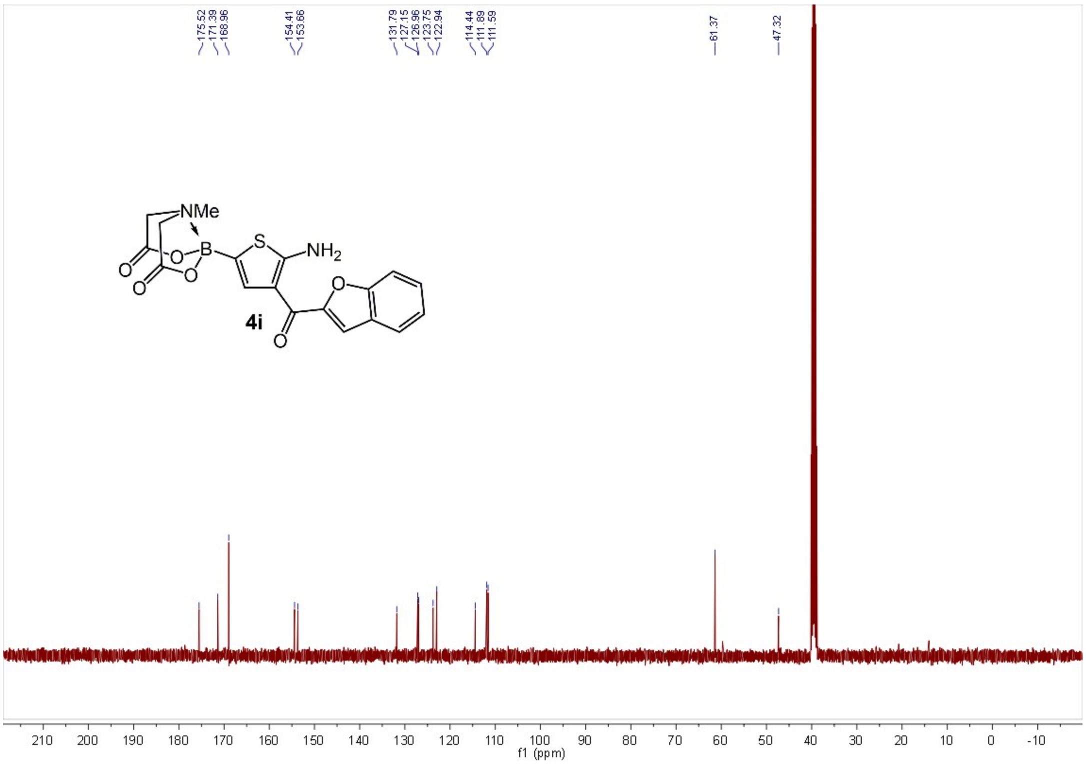
175.52
171.39
168.96
154.41
153.66
131.79
127.15
126.96
123.75
122.94
114.44
111.89
111.59
61.37
47.32
NMe
NH2
4i
210 200 190 180 170 160 150 140 130 120 110 100 90 80 70 60 50 40 30 20 10 0 -10
f1 (ppm)

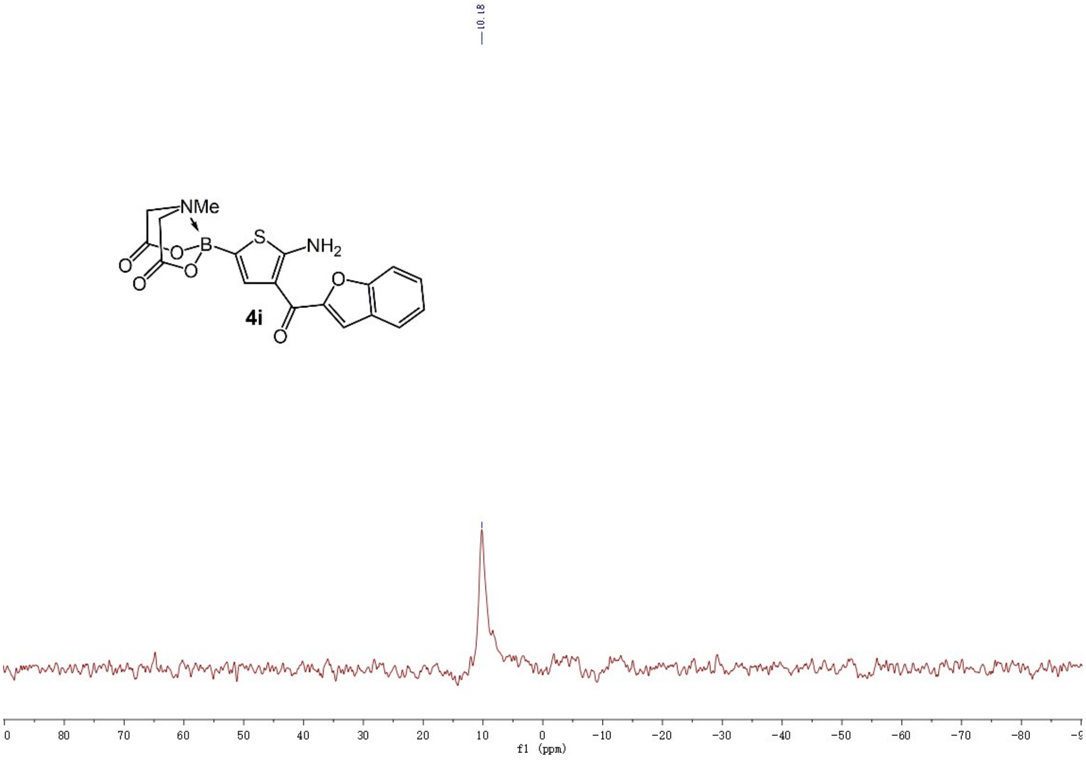
10.18
NMe
NH2
4i
f1 (ppm)

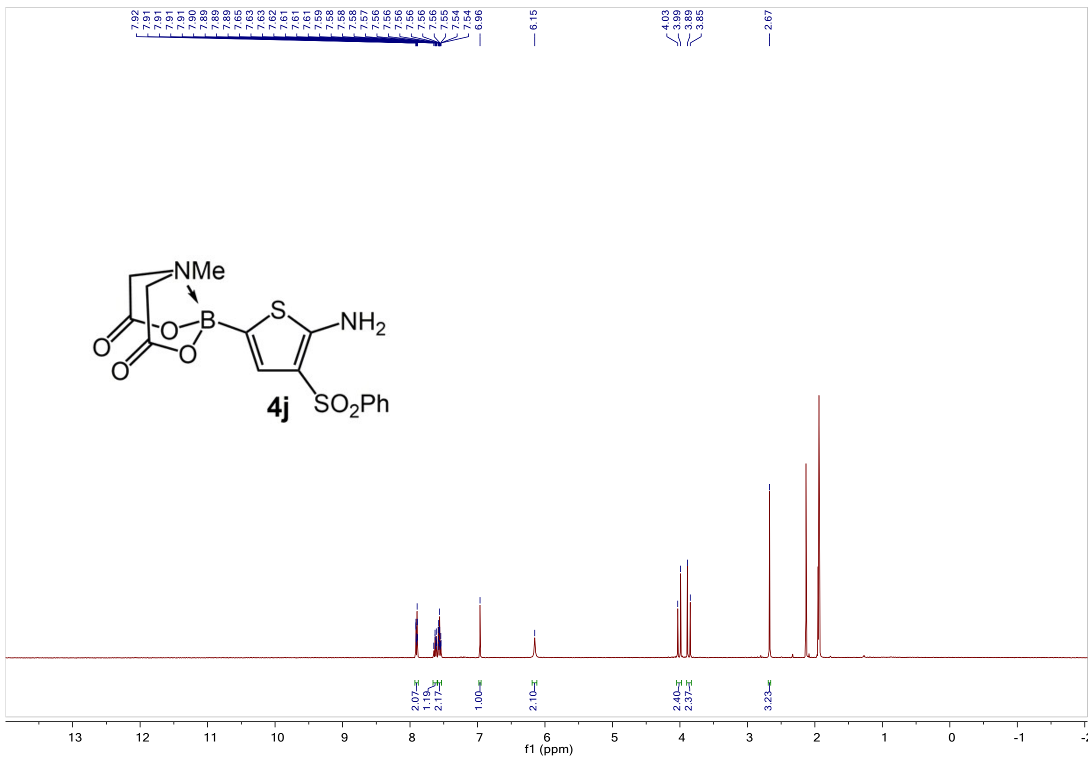
7.92 7.91 7.91 7.91 7.90 7.89 7.89 7.89 7.65 7.63 7.63 7.62 7.61 7.61 7.61 7.61 7.59 7.58 7.58 7.58 7.57 7.56 7.56 7.56 7.56 7.56 7.55 7.54 7.54 6.96
6.15
4.03 3.99 3.89 3.85
2.67
NMe
B
O
O
S
NH_2
SO_2Ph
4j
2.07 1.19 2.17 1.00 2.10 2.40 2.37 3.23
f1 (ppm)

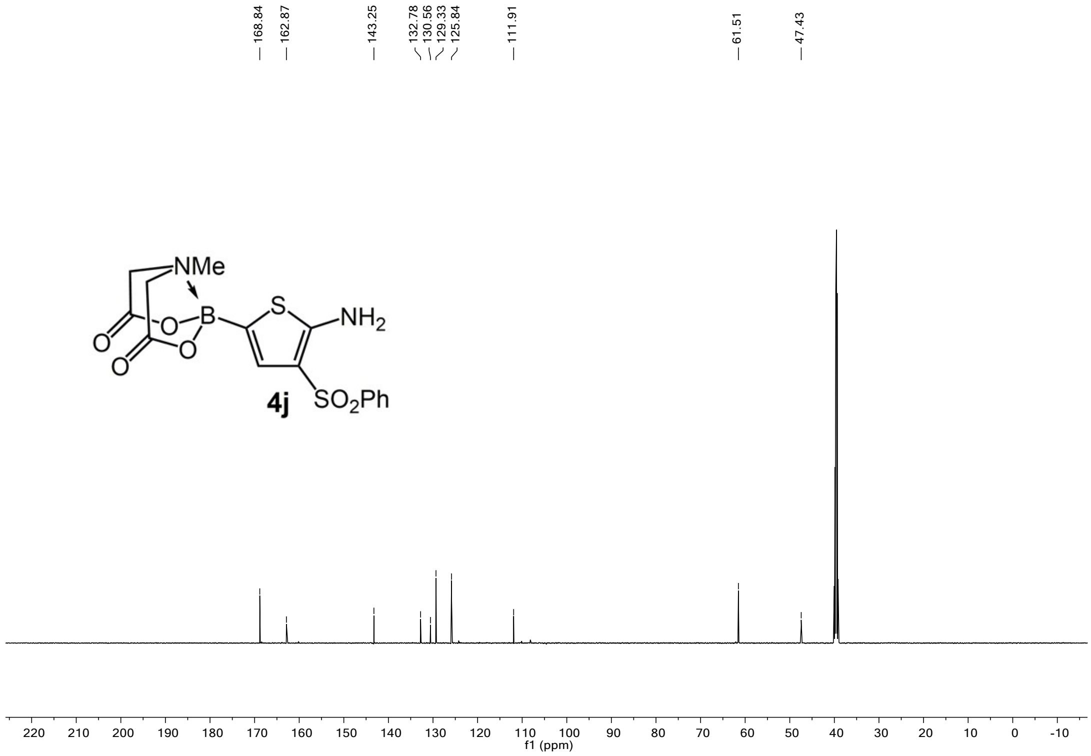
168.84
162.87
143.25
132.78 130.56 129.33 125.84
111.91
61.51
47.43
NMe
B
O
O
S
NH_2
SO_2Ph
4j
f1 (ppm)

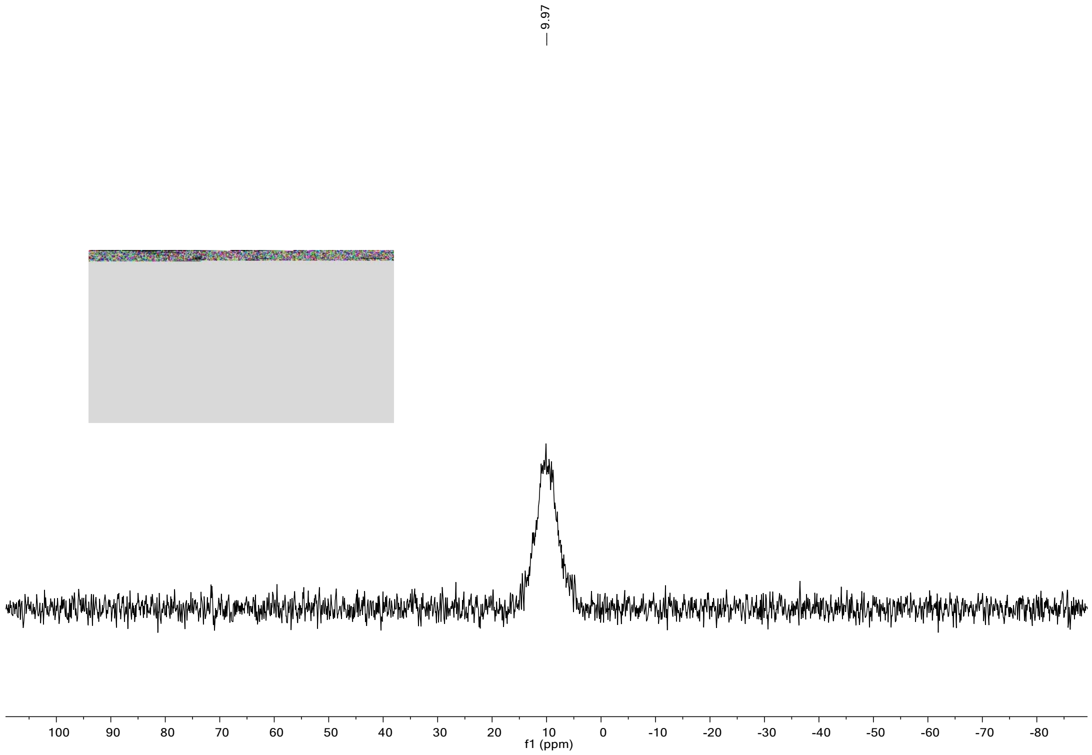
9.97
f1 (ppm)

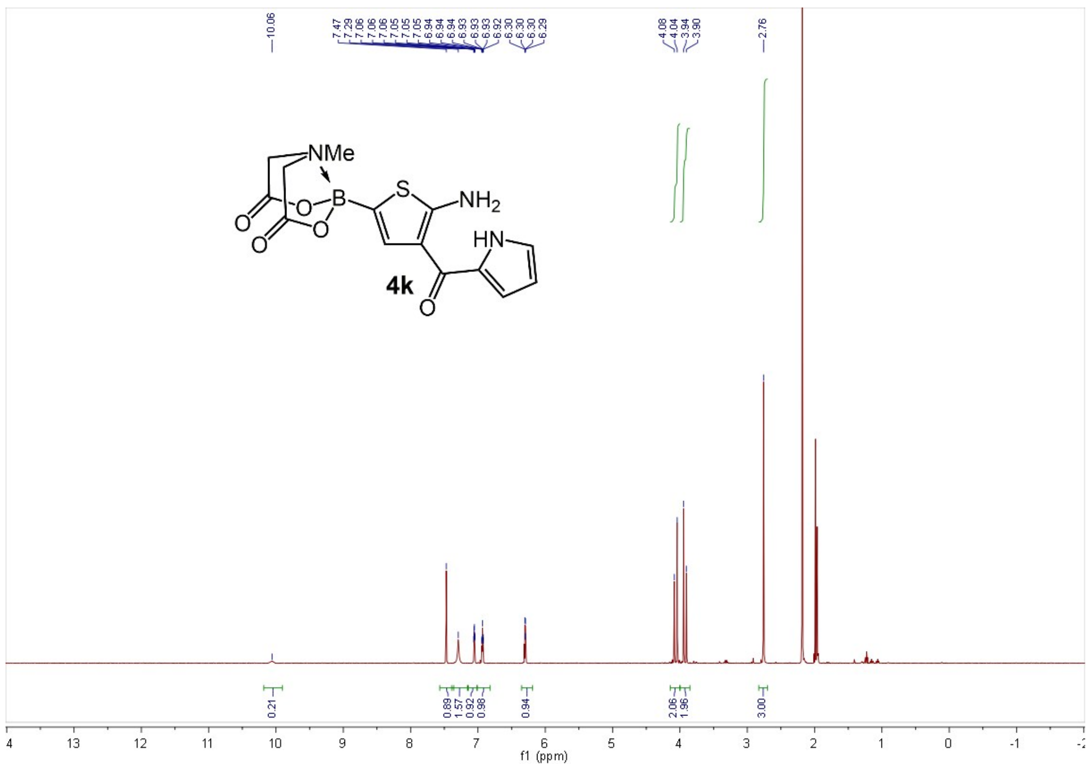
10.06
7.47 7.29 7.06 7.06 7.06 7.05 7.05 7.05 6.94 6.94 6.94 6.93 6.93 6.92 6.30 6.30 6.30 6.29
4.08 4.04 3.94 3.90
2.76
NMe
B
O
S
NH2
HN
4k
0.21
0.89 1.57 0.92 0.98
0.94
2.06 1.96
3.00
f1 (ppm)

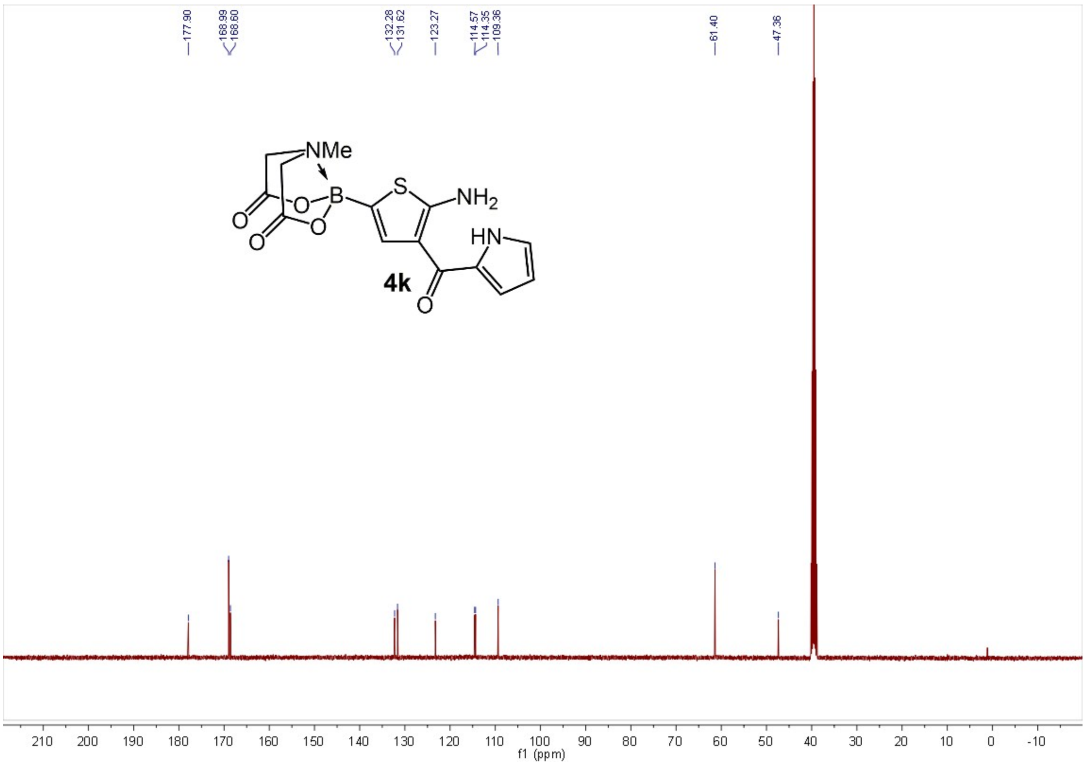
177.90
168.99
168.60
132.28
131.62
123.27
114.57
114.35
109.36
61.40
47.36
NMe
B
O
S
NH2
HN
4k
f1 (ppm)

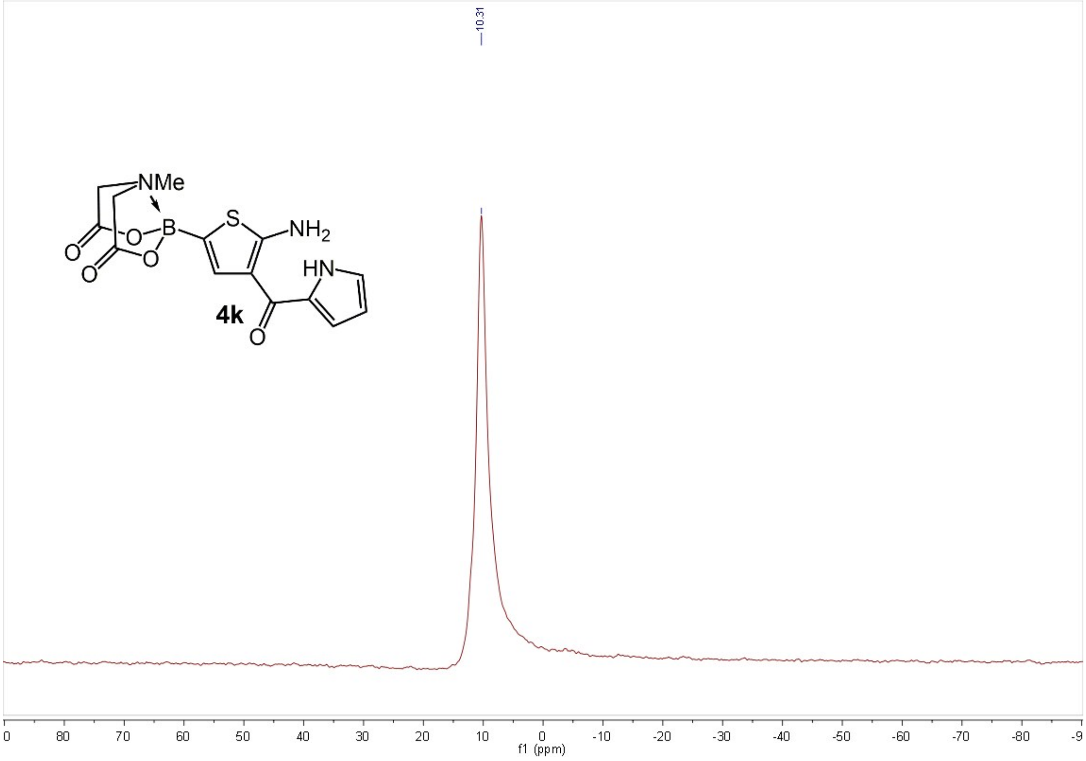
10.31
NMe
B
O
S
NH2
HN
4k
f1 (ppm)

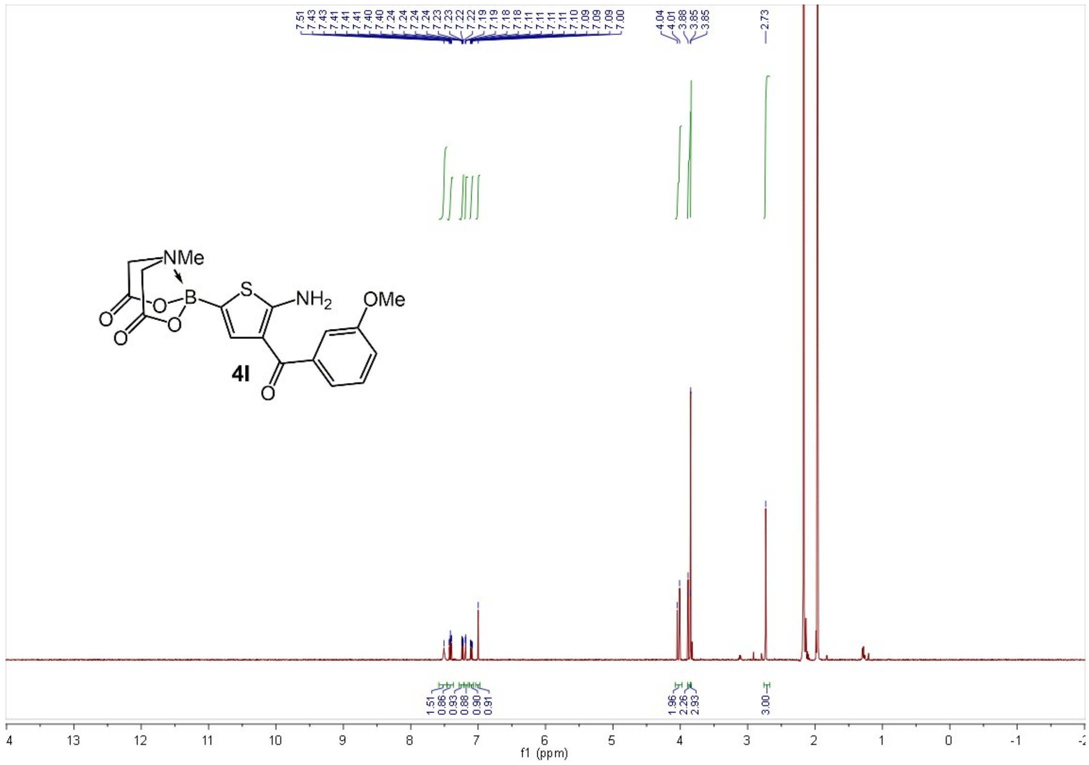

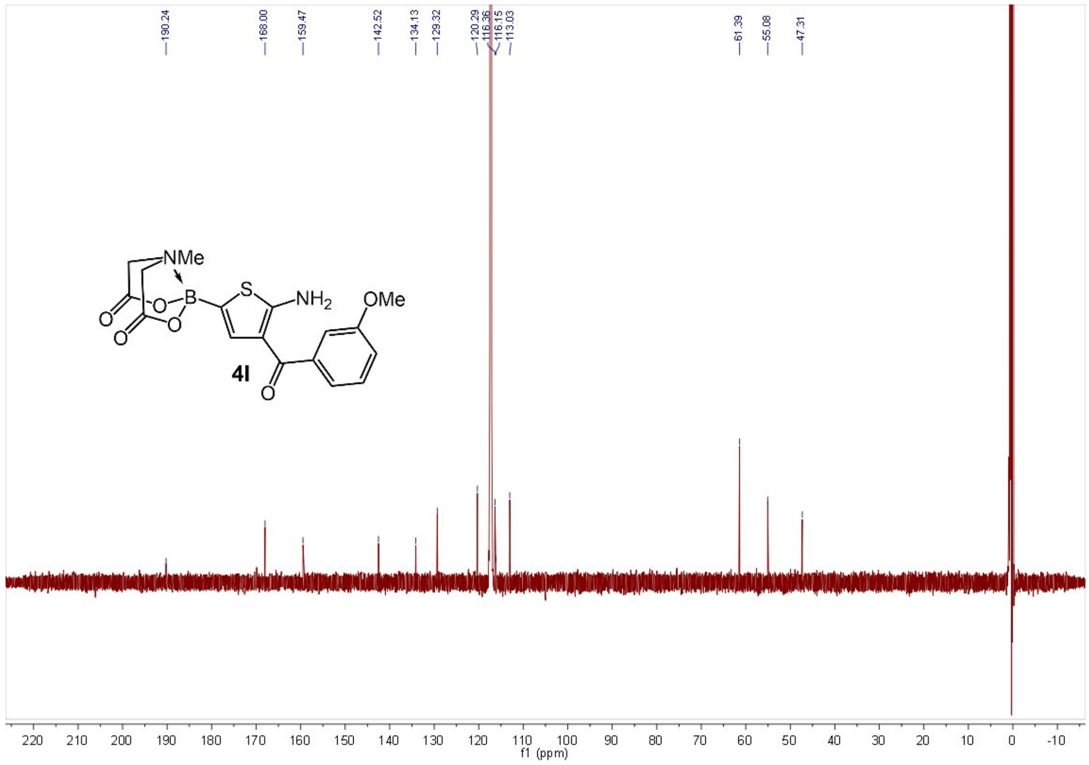

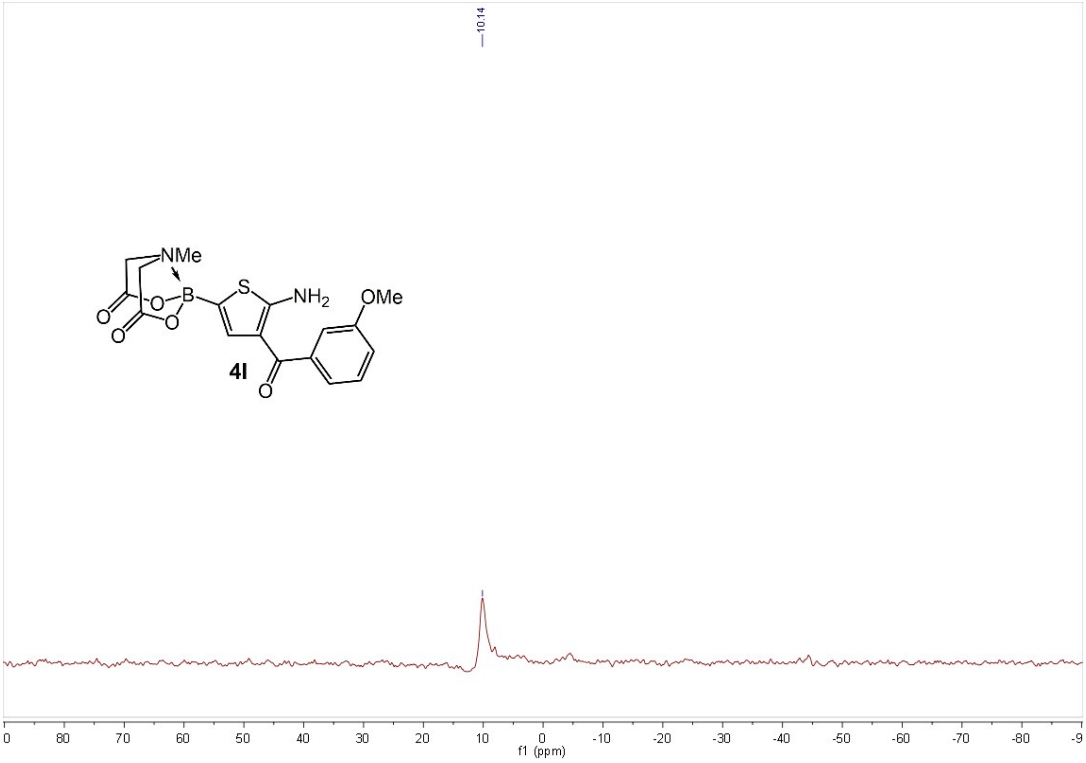

4l

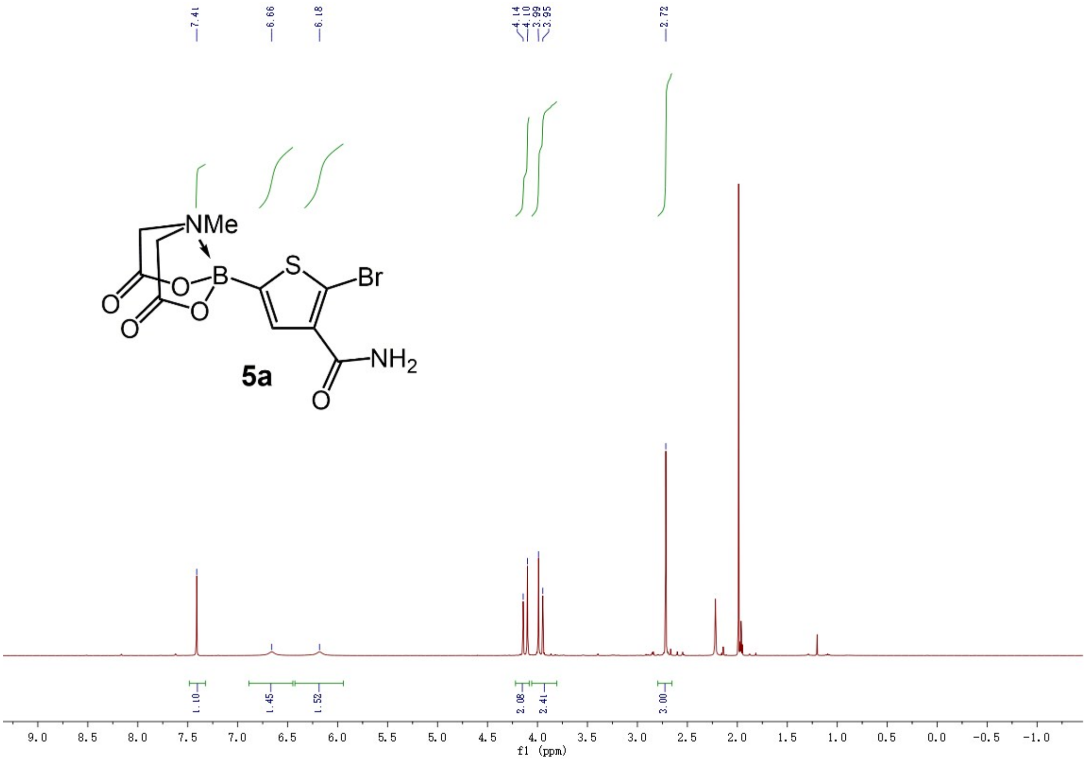

5a

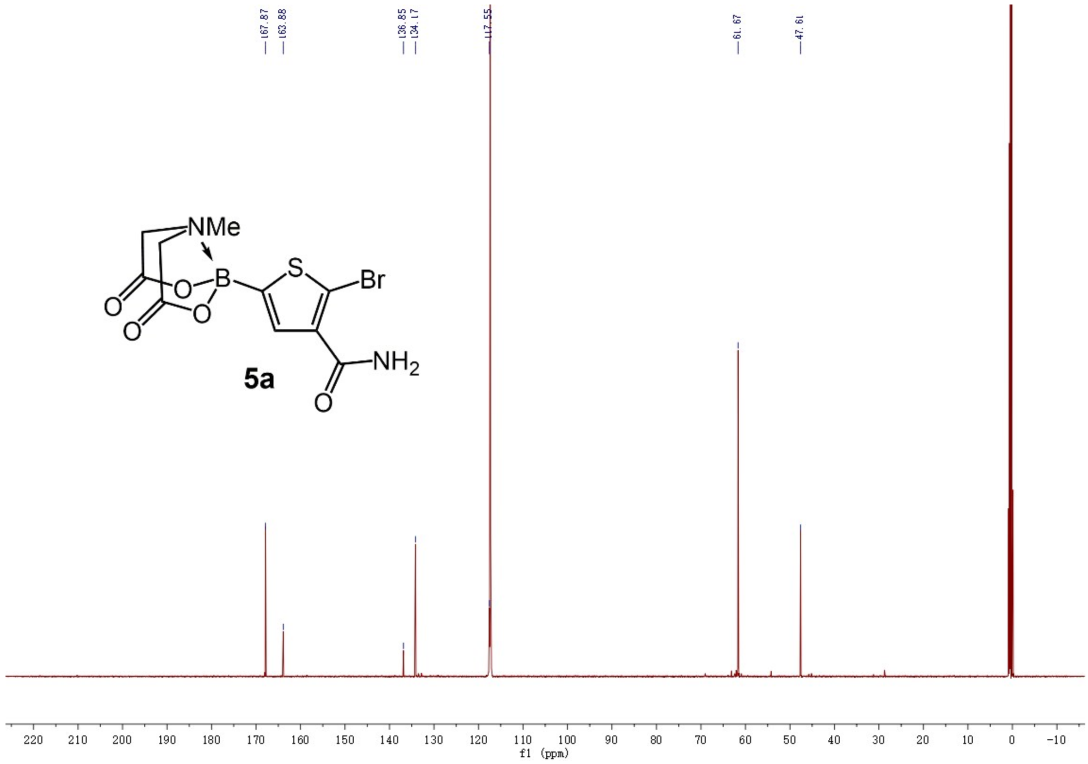
167.87
163.88
136.85
134.17
117.55
61.67
47.61
NMe
B
O
O
O
O
S
Br
NH2
O
5a
220 210 200 190 180 170 160 150 140 130 120 110 100 90 80 70 60 50 40 30 20 10 0 -10
f1 (ppm)

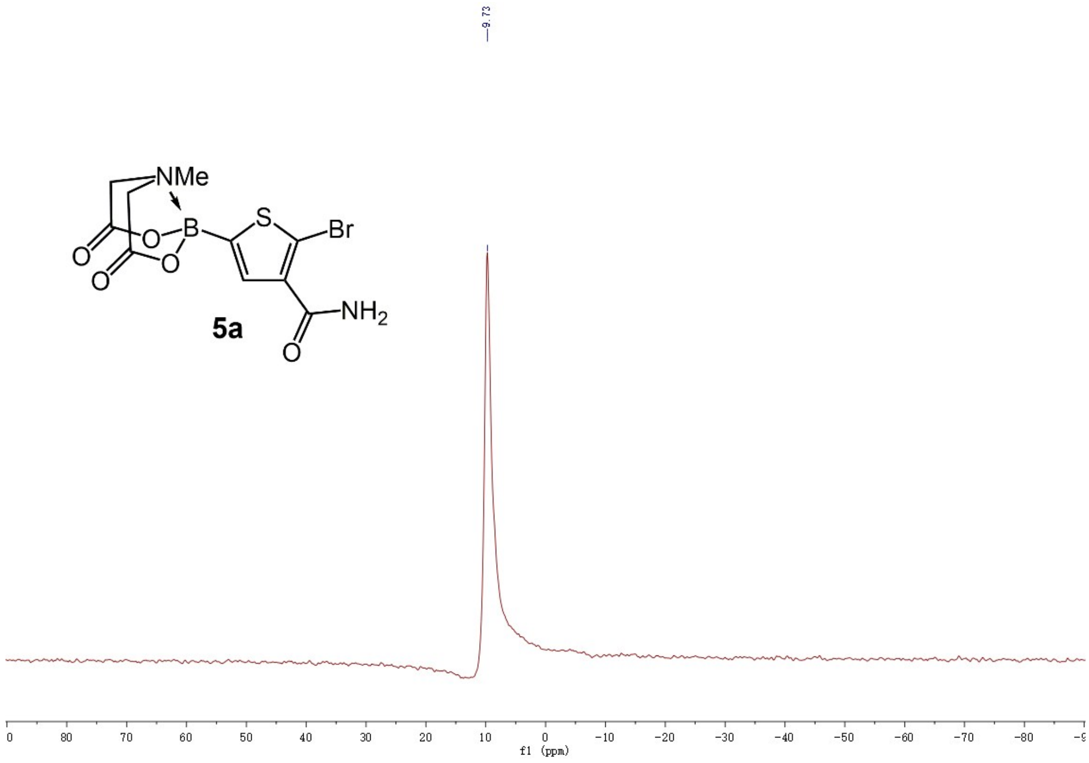
9.73
NMe
B
O
O
O
O
S
Br
NH2
O
5a
80 70 60 50 40 30 20 10 0 -10 -20 -30 -40 -50 -60 -70 -80
f1 (ppm)

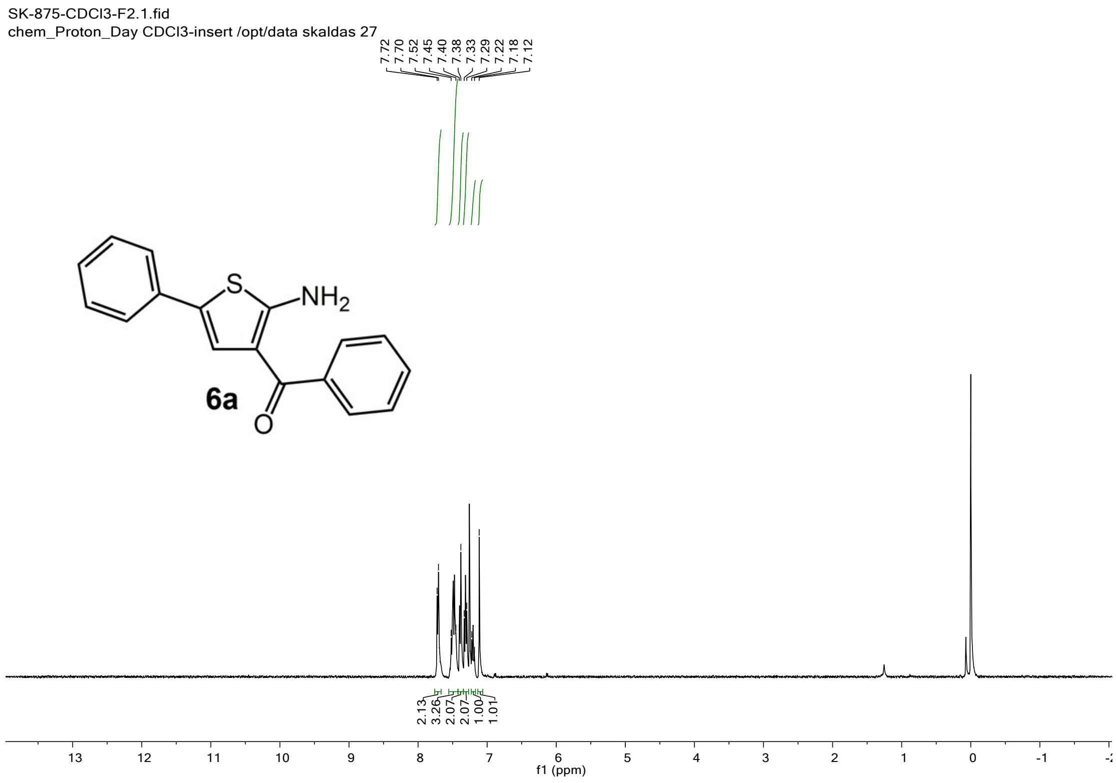
SK-875-CDCl3-F2.1.fid
chem_Proton_Day CDCl3-insert /opt/data skaldas 27
7.72 7.70 7.52 7.45 7.40 7.38 7.33 7.29 7.22 7.18 7.12
S
NH2
O
6a
2.13 3.26 2.07 2.07 1.00 1.01
13 12 11 10 9 8 7 6 5 4 3 2 1 0 -1
f1 (ppm)

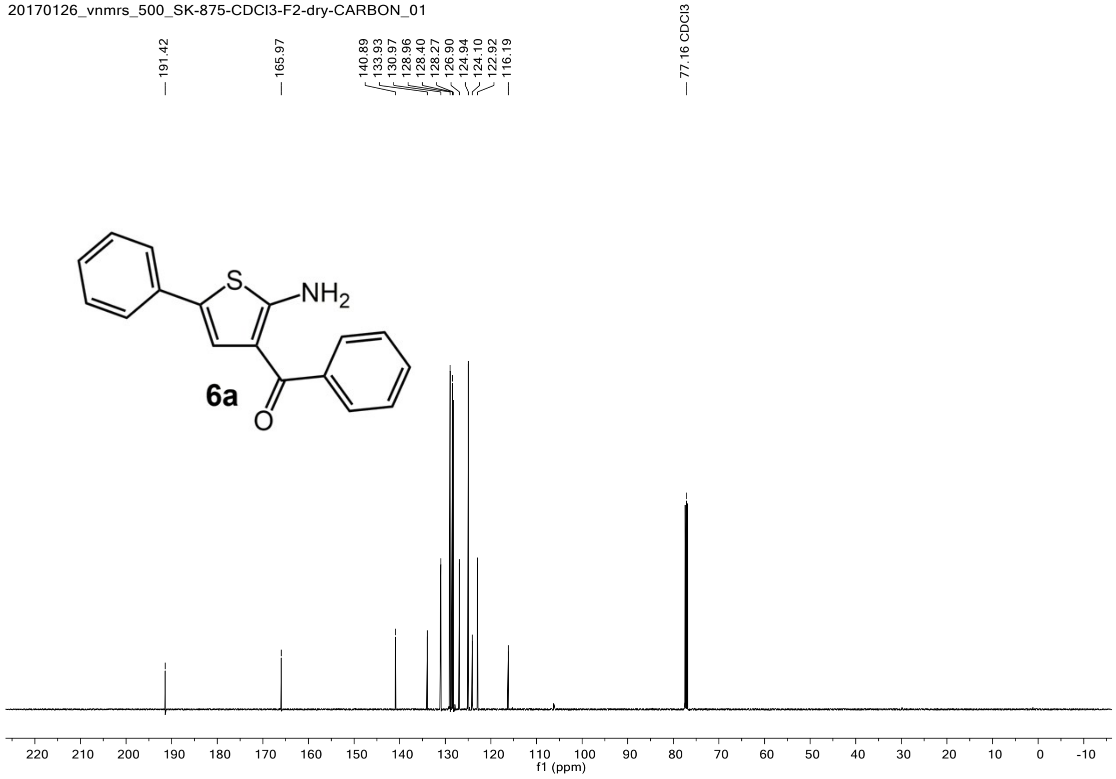
20170126_vnmrs_500_SK-875-CDCl3-F2-dry-CARBON_01
191.42
165.97
140.89 133.93 130.97 128.96 128.40 128.27 126.90 124.94 124.10 122.92 116.19
77.16 CDCl3
S
NH2
O
6a
220 210 200 190 180 170 160 150 140 130 120 110 100 90 80 70 60 50 40 30 20 10 0 -10
f1 (ppm)

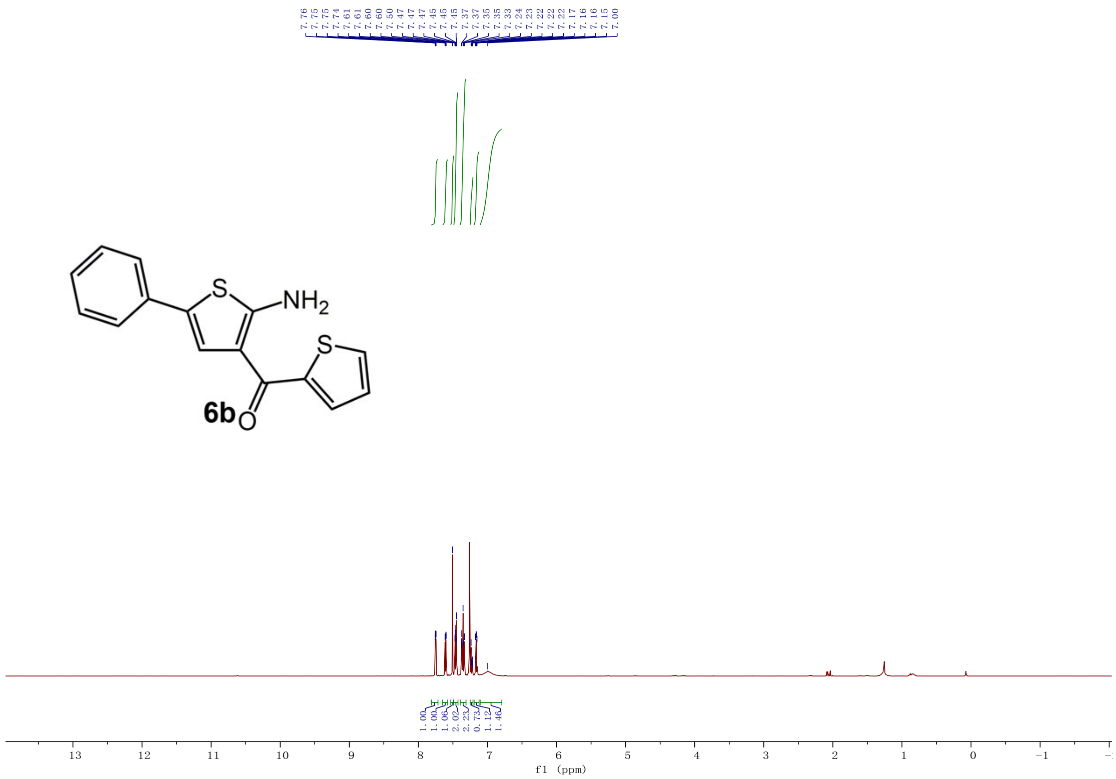
7.76 7.75 7.75 7.74 7.61 7.61 7.60 7.60 7.50 7.47 7.47 7.47 7.45 7.45 7.45 7.37 7.37 7.35 7.35 7.33 7.24 7.23 7.22 7.22 7.22 7.17 7.16 7.16 7.15 7.00
S
NH2
6b
O
1.00 1.00 1.06 2.02 2.23 0.73 1.12 1.46
13 12 11 10 9 8 7 6 5 4 3 2 1 0 −1
f1 (ppm)

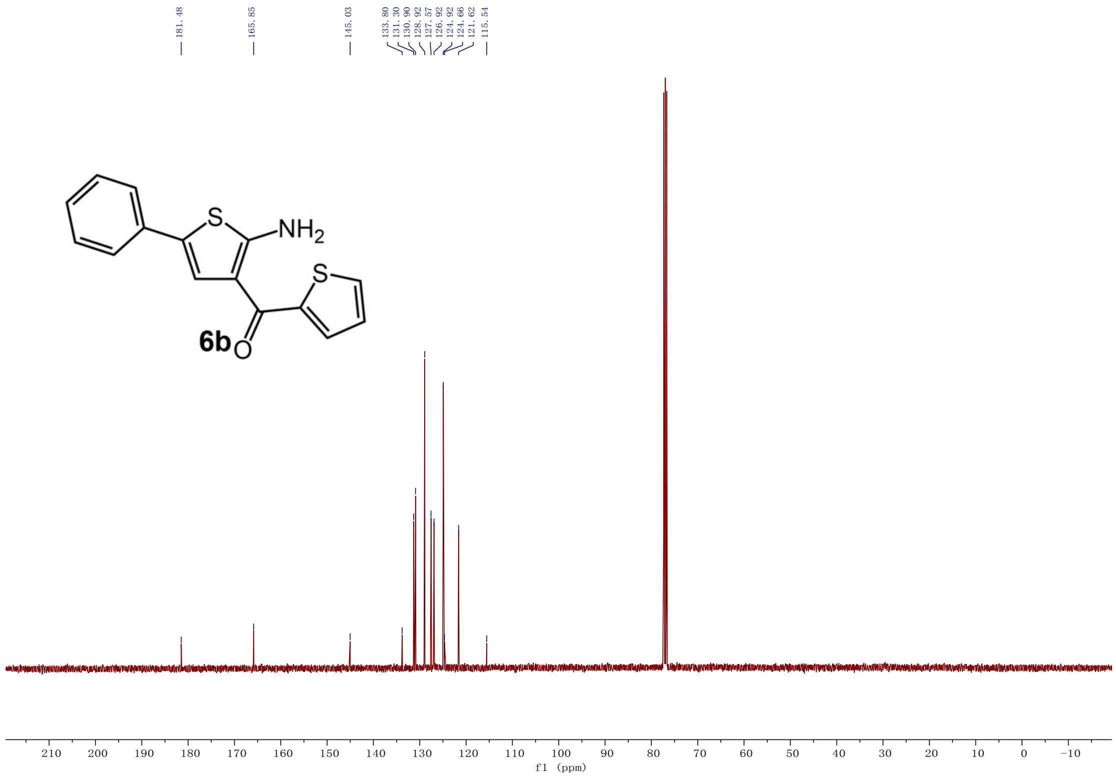
181.48
165.85
145.03
133.80 131.30 130.90 128.92 127.57 126.92 124.92 124.66 121.62 115.54
S
NH2
6b
O
210 200 190 180 170 160 150 140 130 120 110 100 90 80 70 60 50 40 30 20 10 0 −10
f1 (ppm)

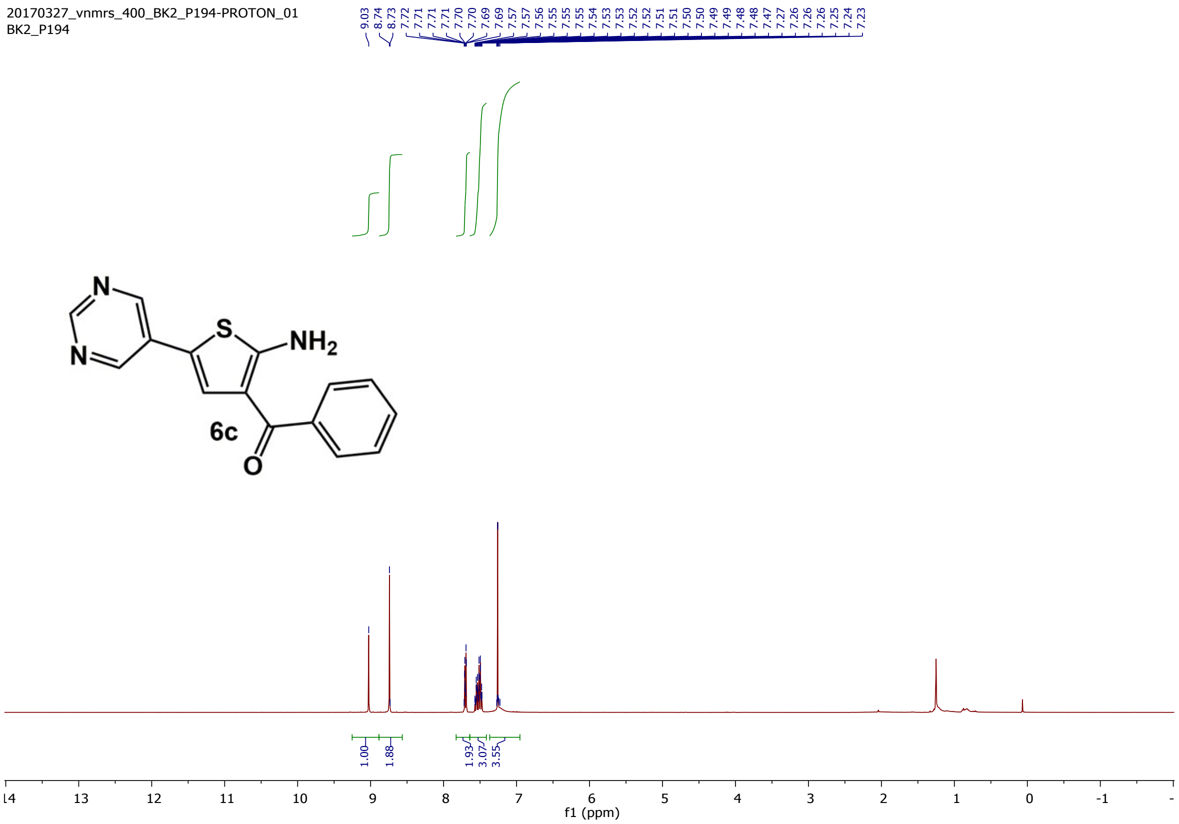
20170327_vnmrs_400_BK2_P194-PROTON_01
BK2_P194
9.03
8.74
8.73
7.72
7.71
7.71
7.70
7.70
7.69
7.69
7.57
7.57
7.56
7.55
7.55
7.55
7.54
7.53
7.53
7.52
7.52
7.51
7.51
7.50
7.50
7.49
7.49
7.48
7.48
7.47
7.27
7.26
7.26
7.26
7.25
7.24
7.23
N
N
S
NH2
6c
O
1.00
1.88
1.93
3.07
3.55
f1 (ppm)

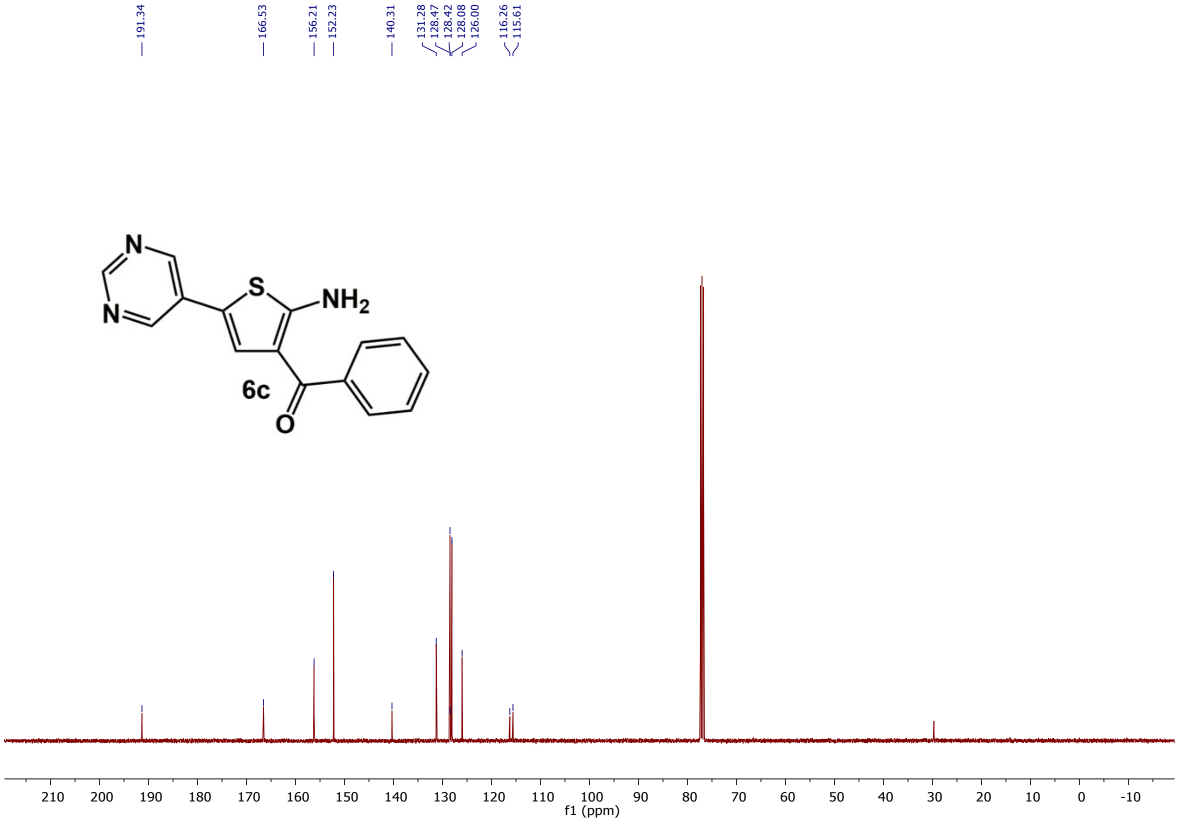
191.34
166.53
156.21
152.23
140.31
131.28
128.47
128.42
128.08
126.00
116.26
115.61
N
N
S
NH2
6c
O
f1 (ppm)

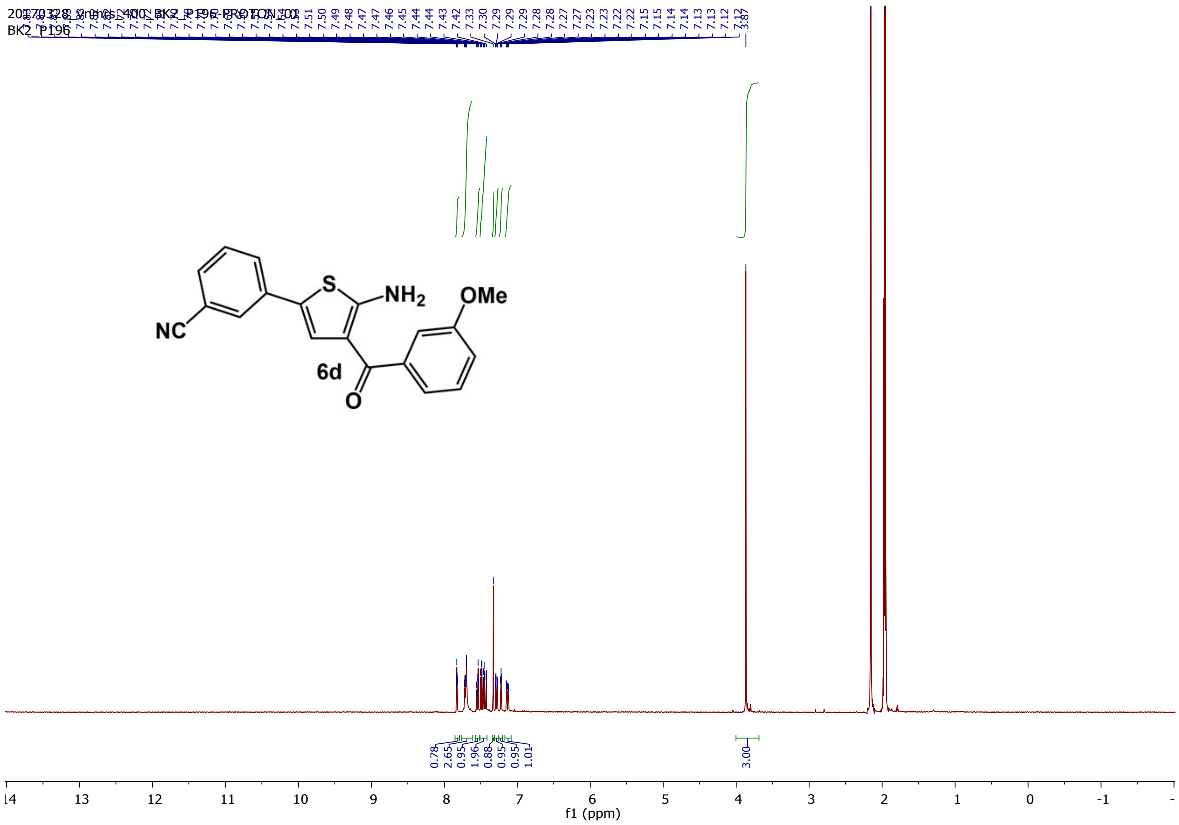
BK2_P196
NH2
OMe
NC
S
6d
O
3.00
f1 (ppm)

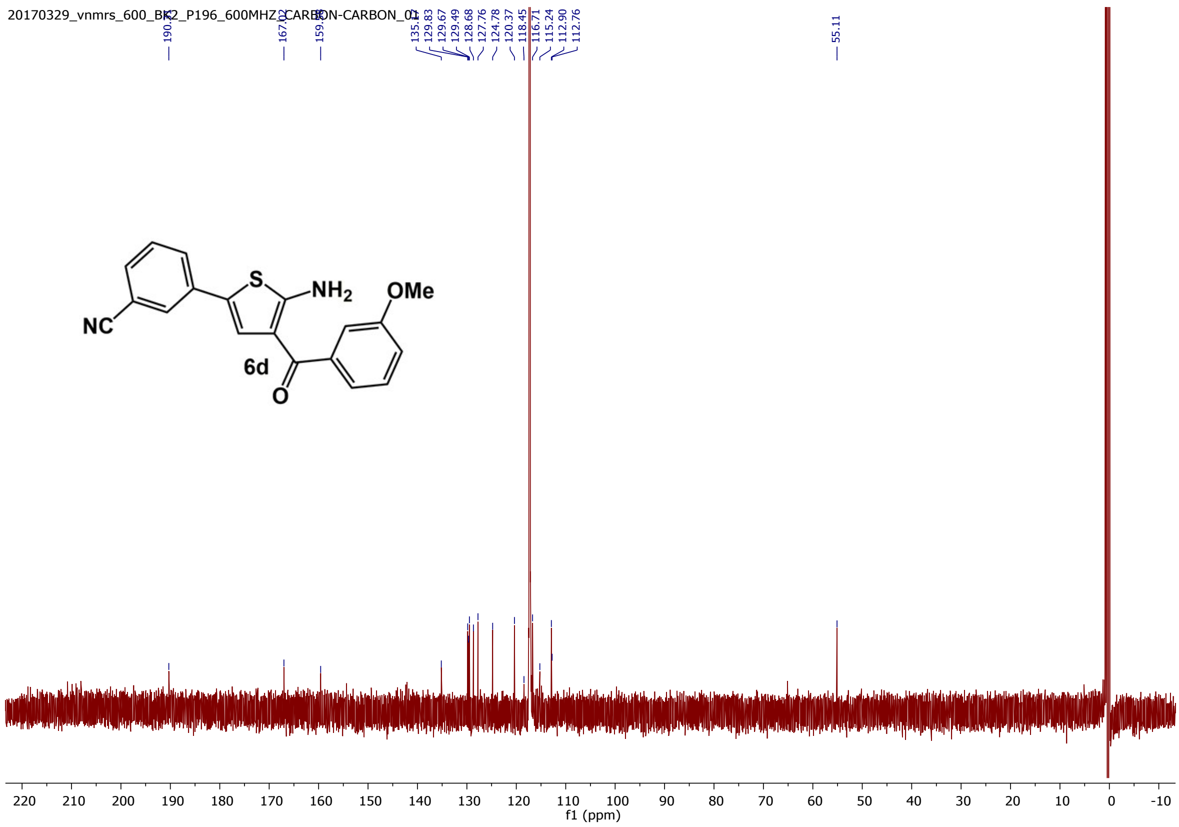
20170329_vnmrs_600_BK2_P196_600MHZ_CARBON-CARBON_01
129.83
129.67
129.49
128.68
127.76
124.78
120.37
118.45
116.71
115.24
112.90
112.76
55.11
NH2
OMe
NC
S
6d
O
f1 (ppm)

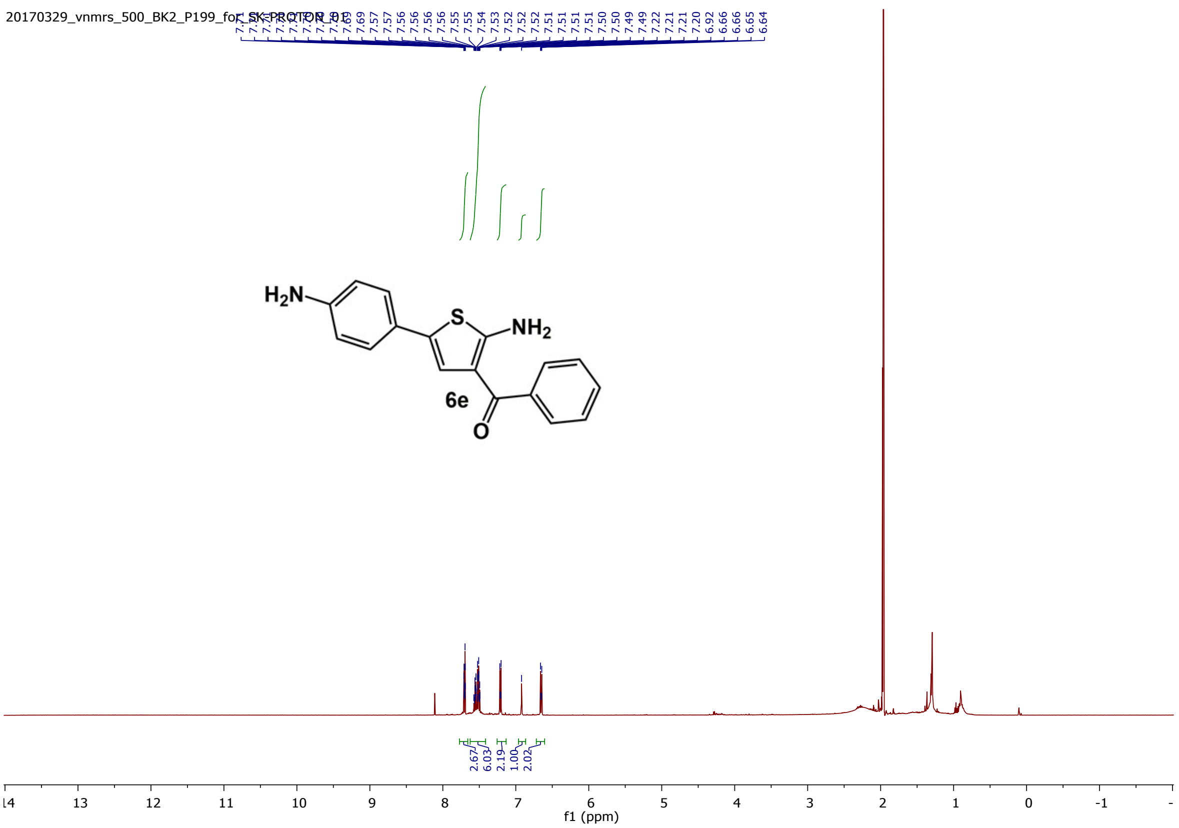

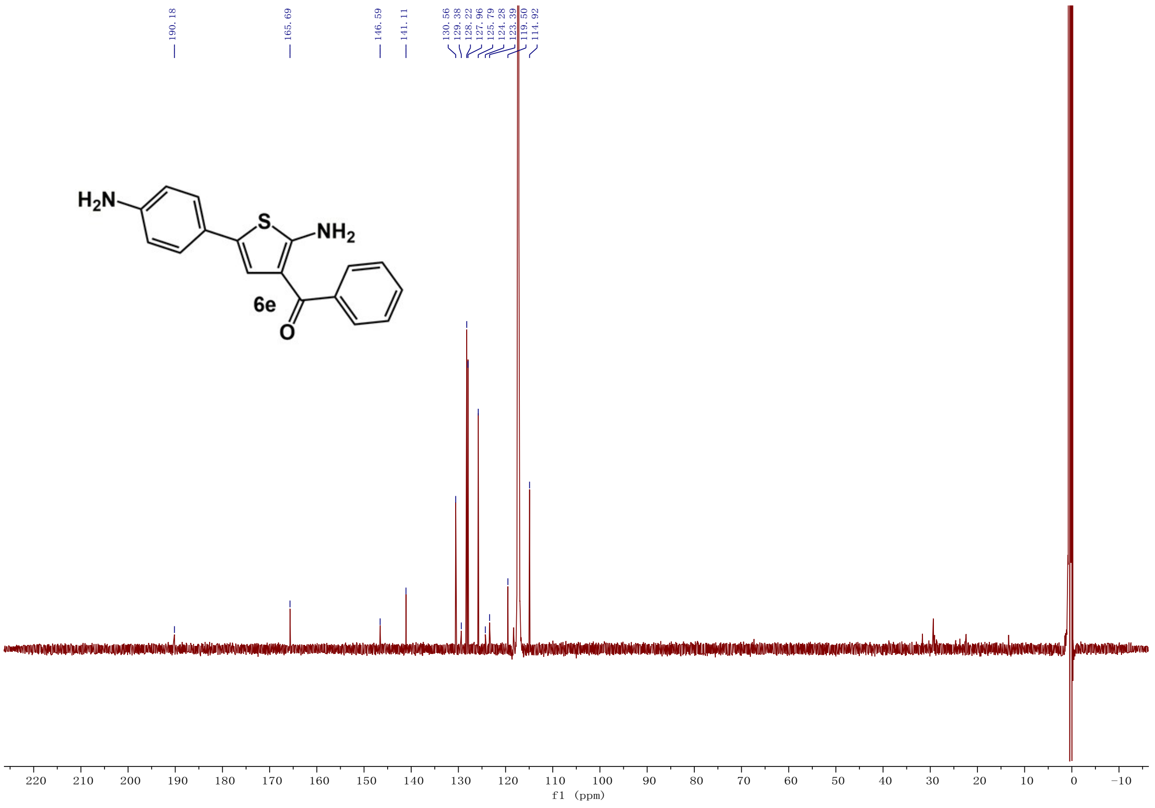

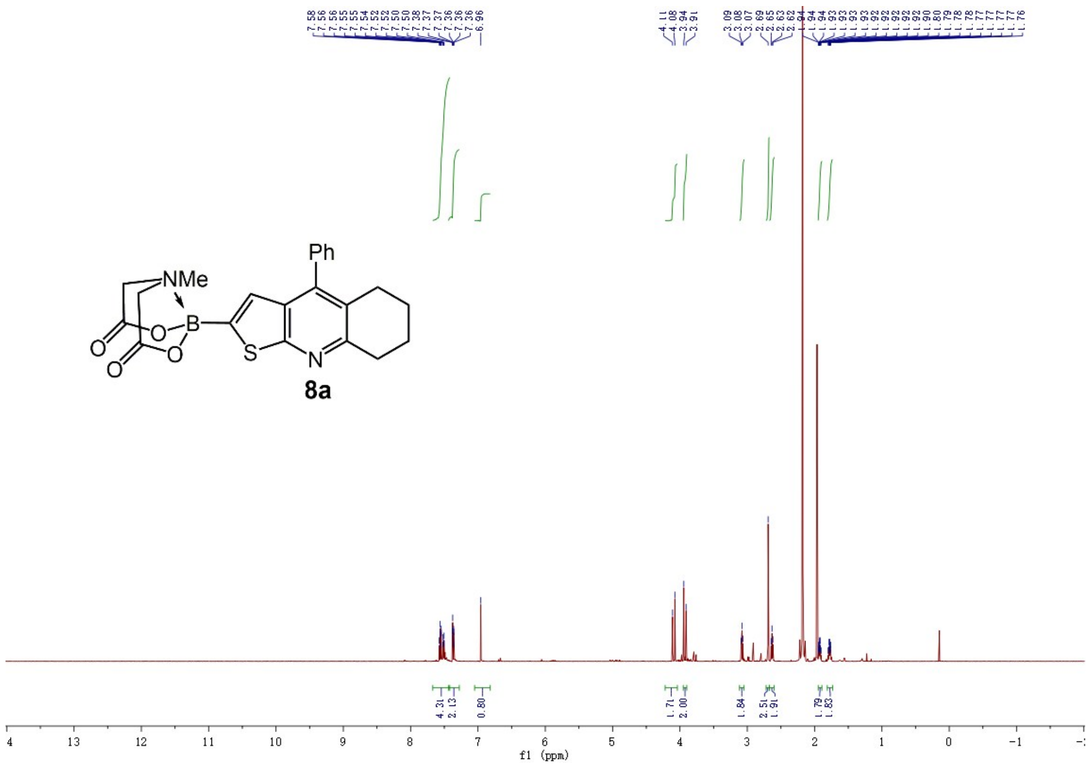

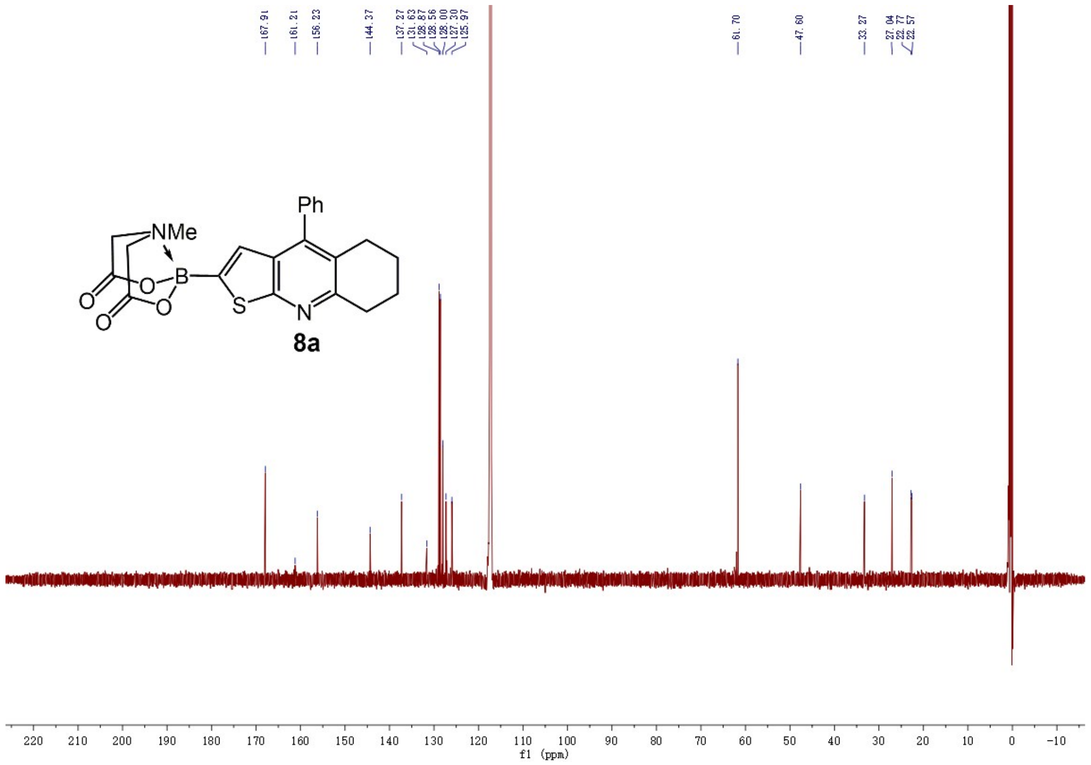

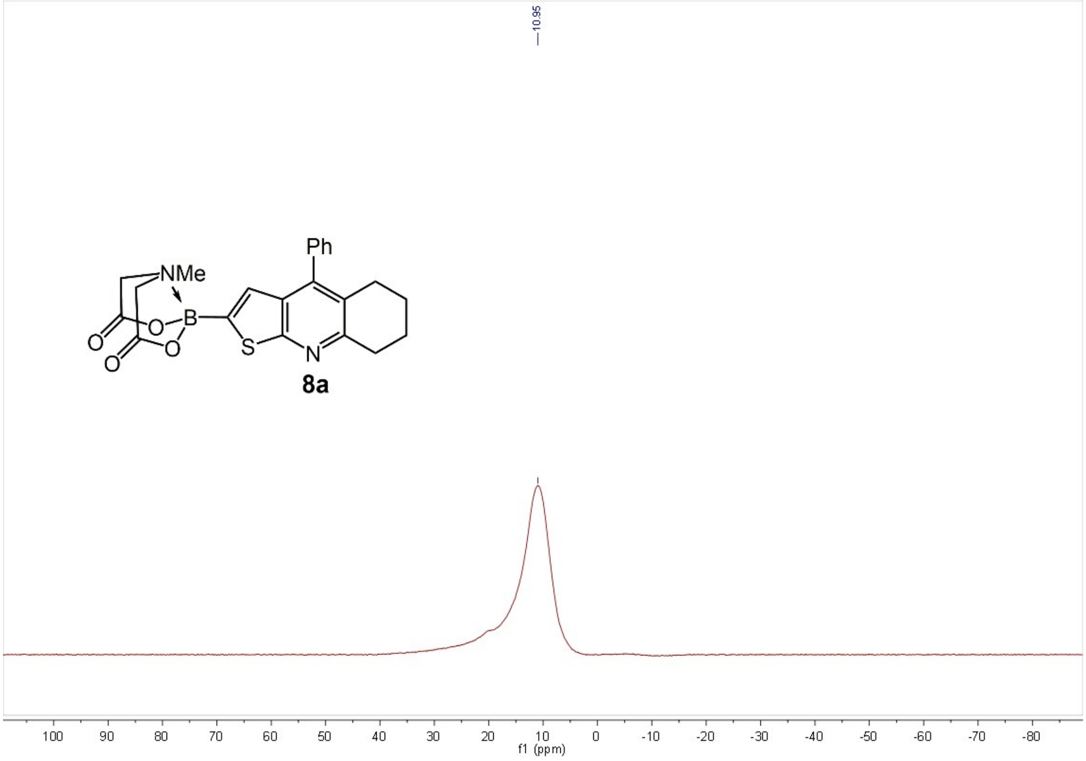

—10.95
Ph
NMe
B
O
O
O
O
S
N
8a
100 90 80 70 60 50 40 30 20 10 0 -10 -20 -30 -40 -50 -60 -70 -80
f1 (ppm)

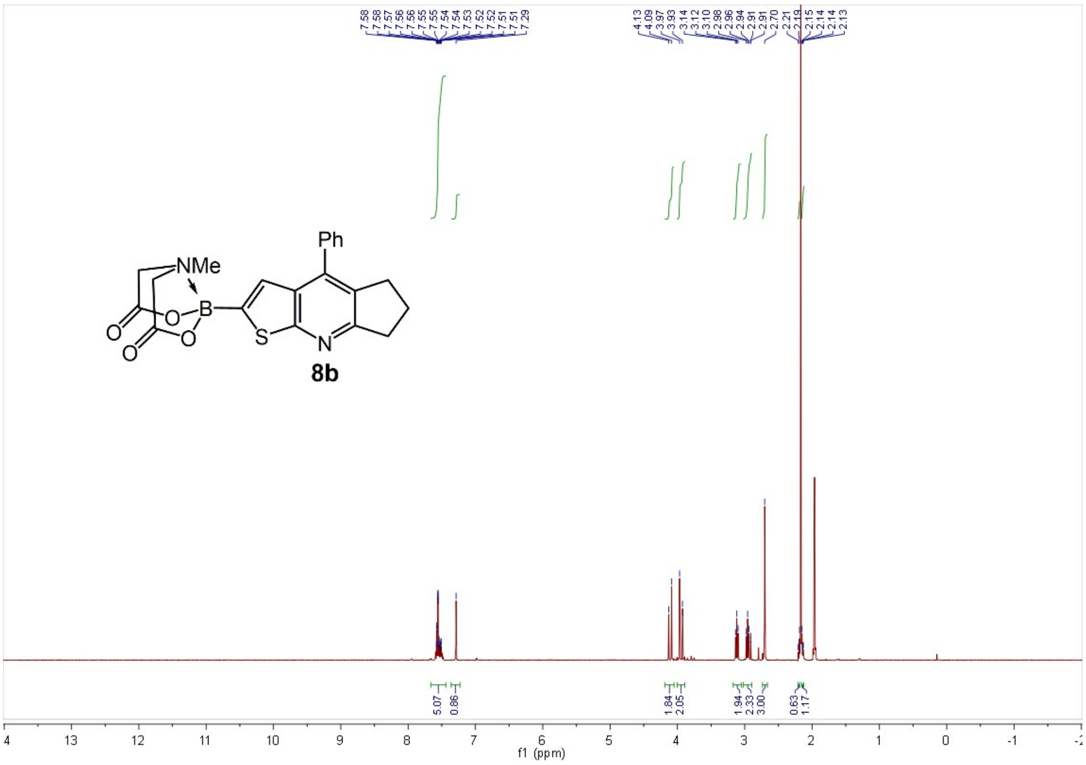

7.58 7.58 7.57 7.56 7.55 7.55 7.54 7.54 7.53 7.52 7.52 7.51 7.51 7.29
4.13 4.09 3.97 3.93 3.14 3.12 3.10 2.98 2.96 2.94 2.91 2.91 2.70 2.21 2.19 2.15 2.14 2.13
Ph
NMe
B
O
O
O
O
S
N
8b
5.07 0.86 1.84 2.05 1.94 2.33 3.00 0.63 1.17
13 12 11 10 9 8 7 6 5 4 3 2 1 0 -1
f1 (ppm)

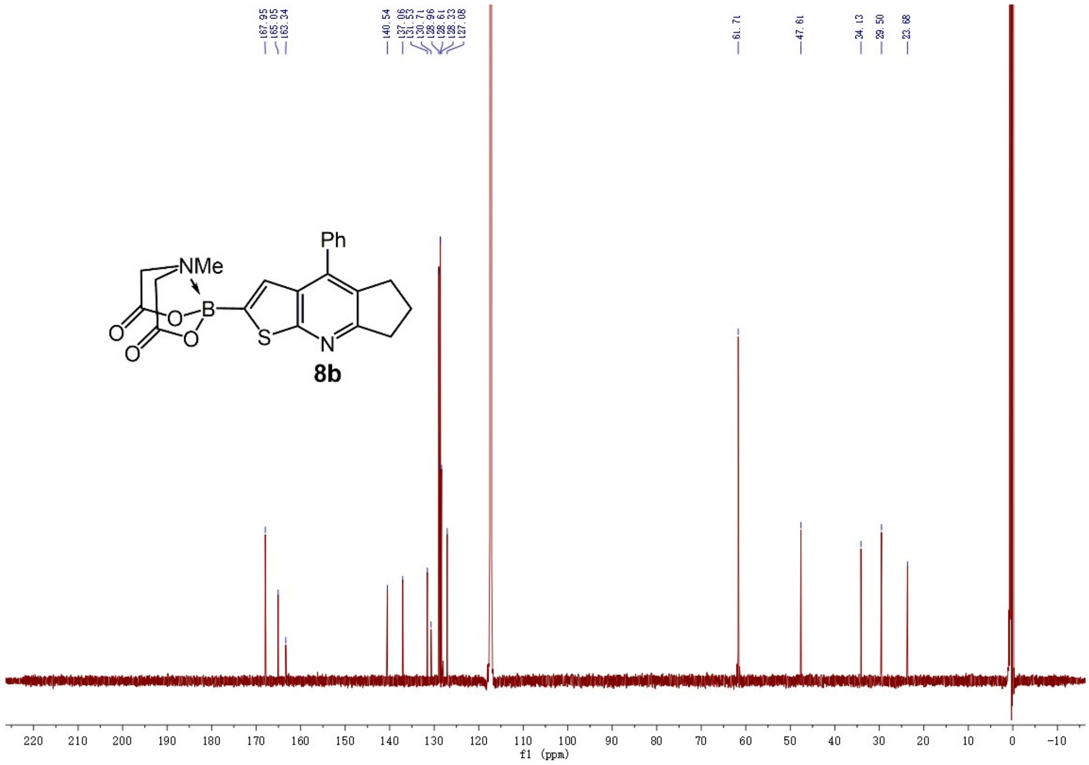
167.95
165.05
163.34
140.54
137.06
131.53
130.71
128.96
128.61
128.23
127.08
61.71
47.61
34.13
29.50
23.68
Ph
NMe
O
B
S
N
8b
220 210 200 190 180 170 160 150 140 130 120 110 100 90 80 70 60 50 40 30 20 10 0 -10
f1 (ppm)

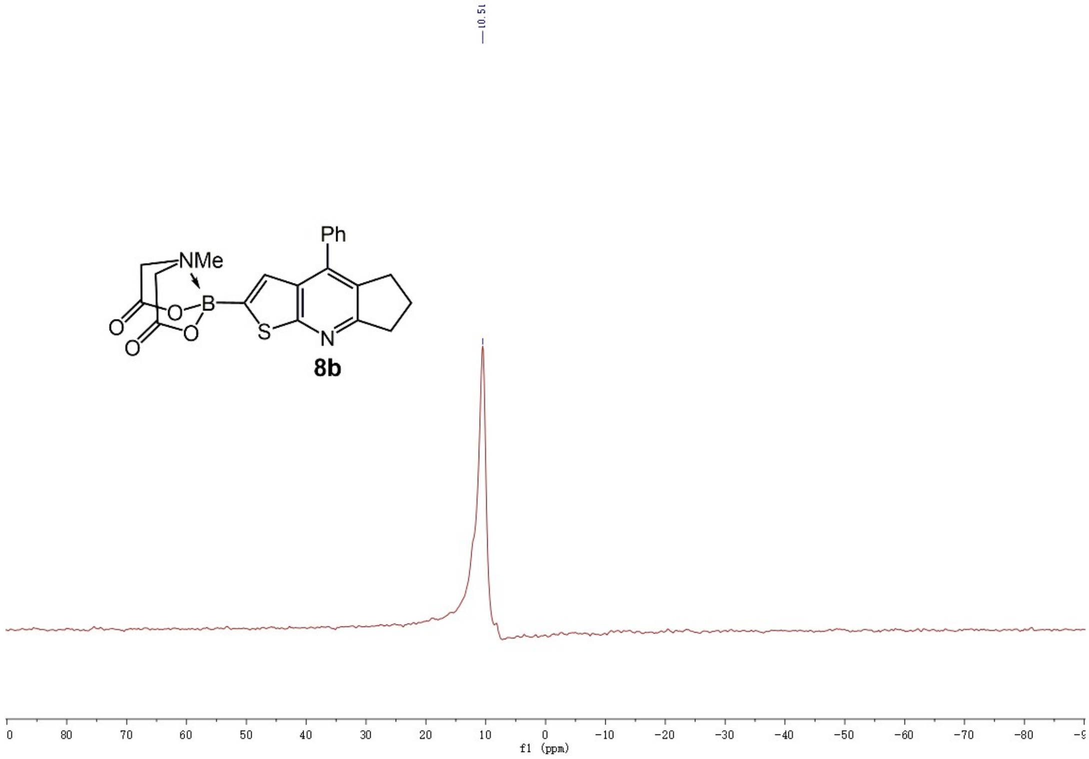
10.51
Ph
NMe
O
B
S
N
8b
80 70 60 50 40 30 20 10 0 -10 -20 -30 -40 -50 -60 -70 -80
f1 (ppm)

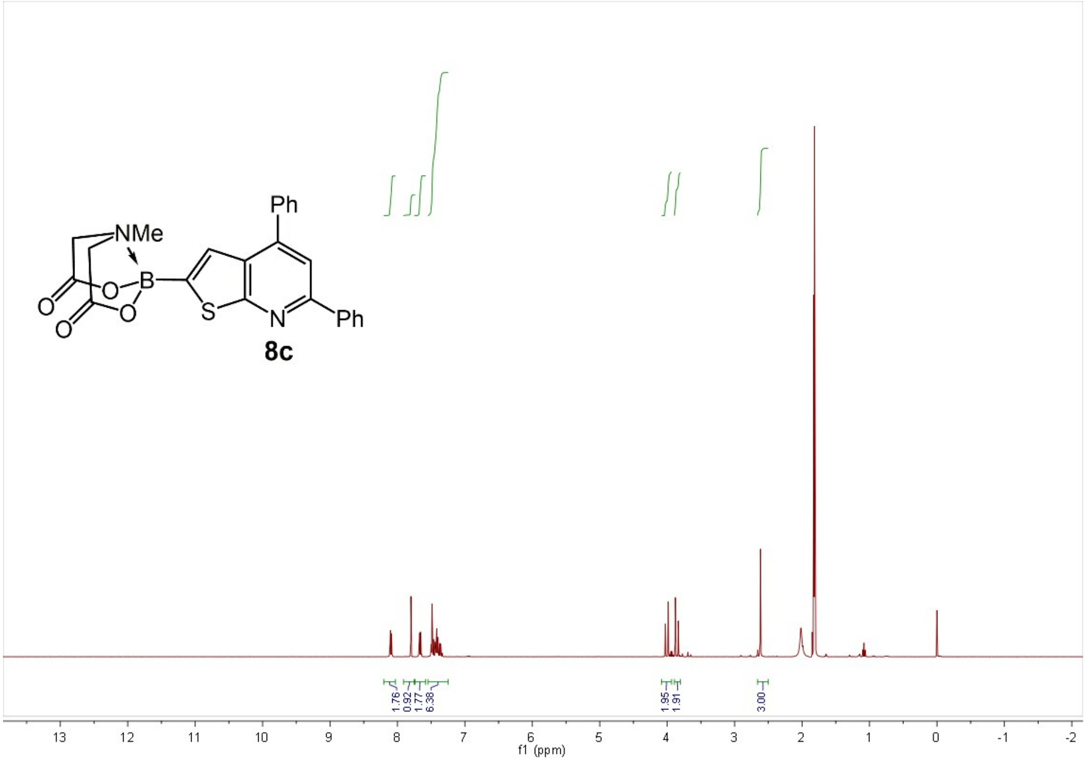
Ph
NMe
B
O
O
O
O
S
N
Ph
8c
1.76
0.92
1.77
6.38
1.95
1.91
3.00
f1 (ppm)

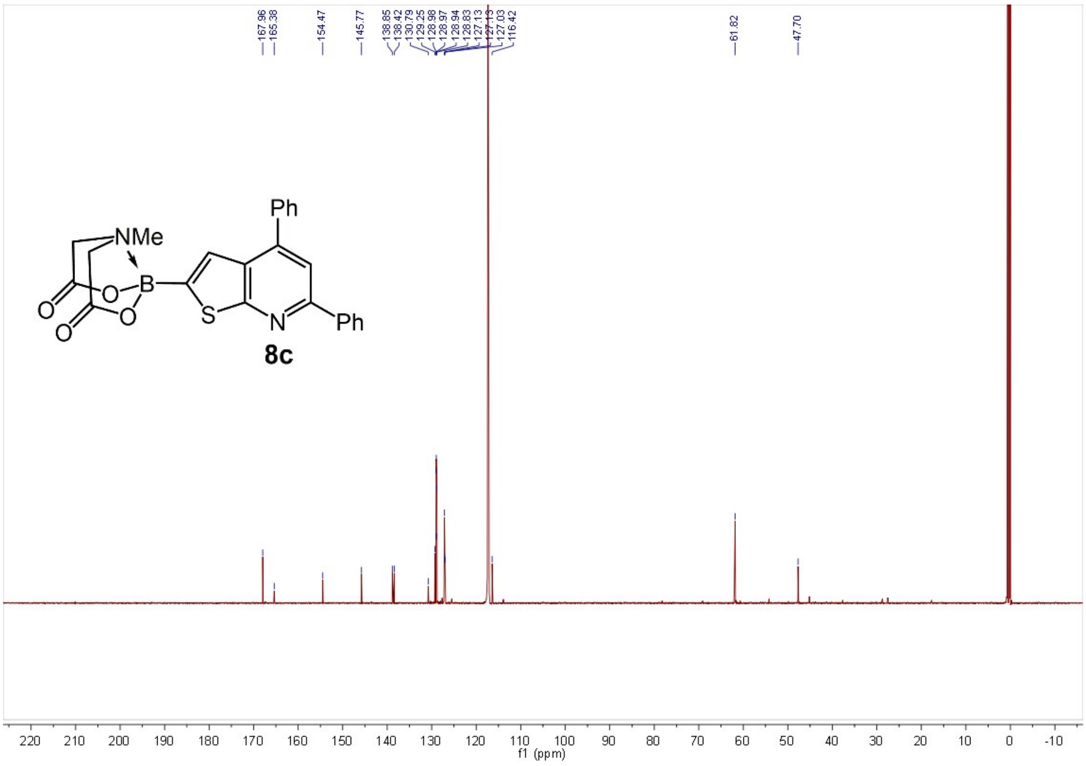
167.96
165.38
154.47
145.77
138.85
138.42
130.79
129.25
128.98
128.97
128.94
128.83
127.13
127.03
116.42
61.82
47.70
Ph
NMe
B
O
O
O
O
S
N
Ph
8c
f1 (ppm)

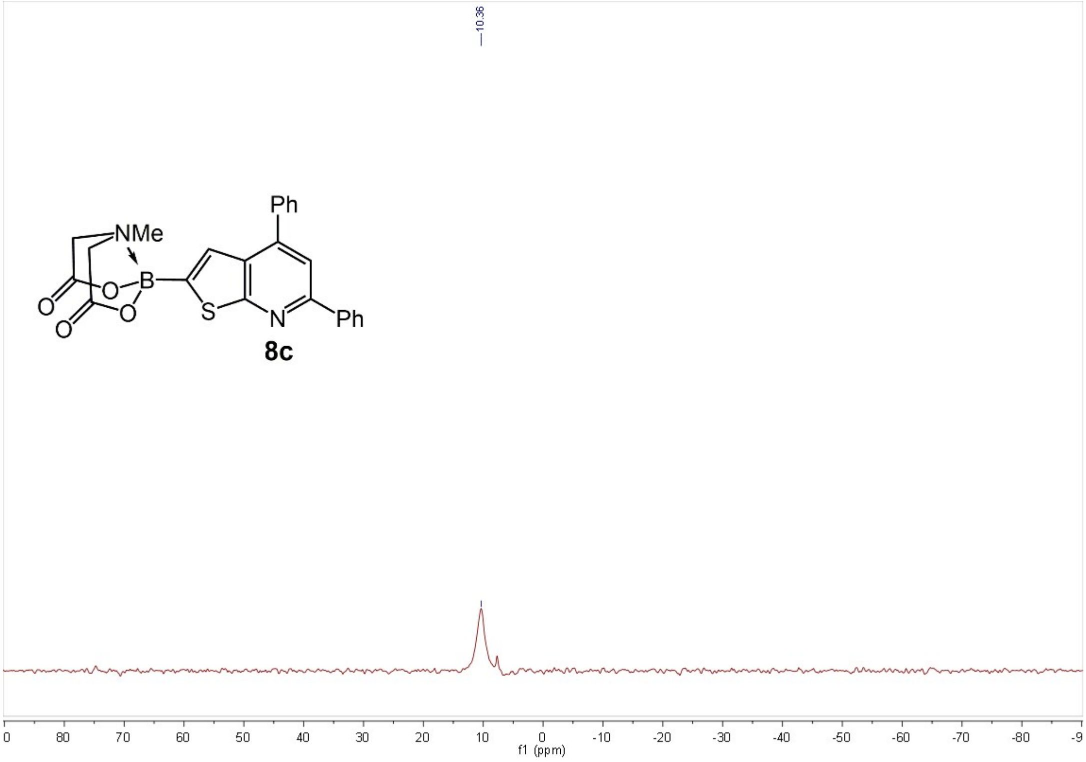
10.36
NMe
B
O
O
O
O
S
N
Ph
Ph
8c
f1 (ppm)

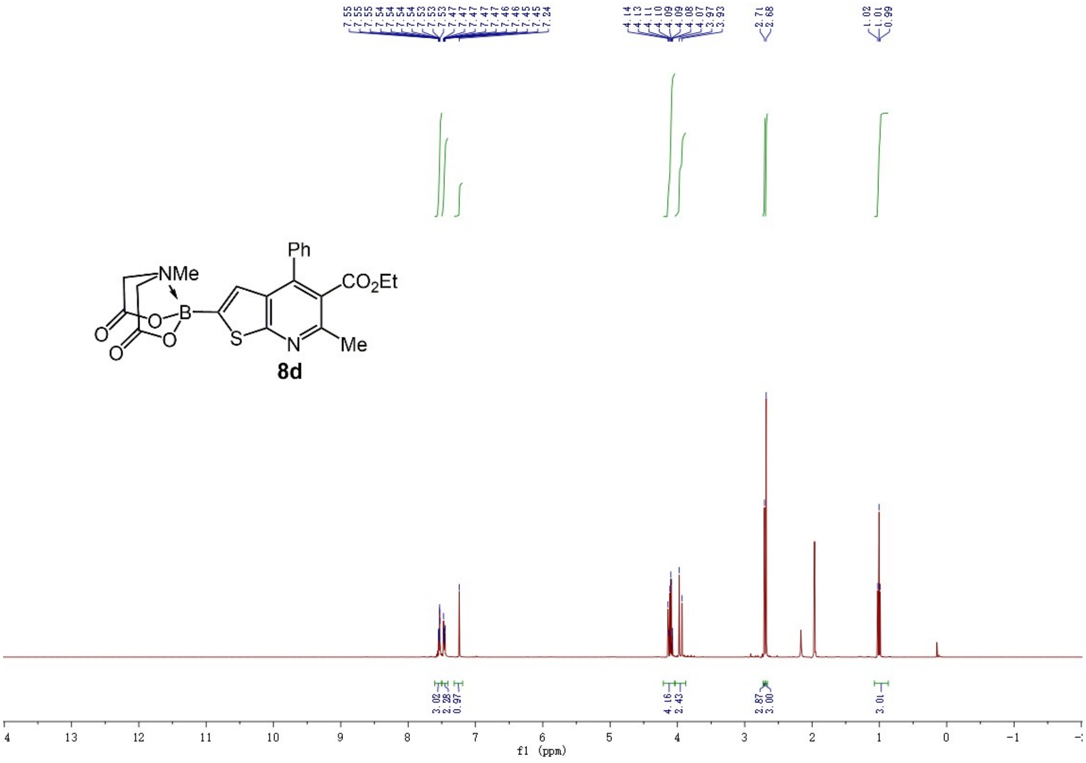
NMe
B
O
O
O
O
S
N
Ph
CO_2Et
Me
8d
f1 (ppm)

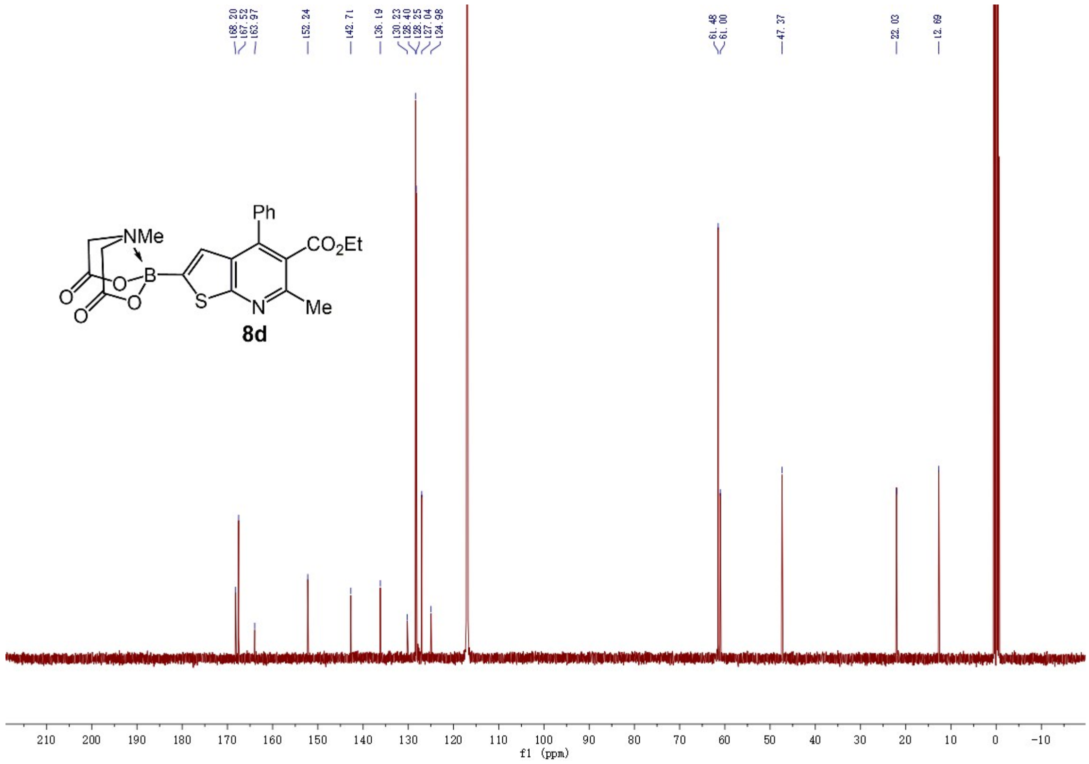
168.20
167.52
163.97
152.24
142.71
136.19
130.23
128.40
128.25
127.04
124.98
61.48
61.00
47.37
22.03
12.69
Ph
NMe
B
O
CO2Et
S
N
Me
8d
210 200 190 180 170 160 150 140 130 120 110 100 90 80 70 60 50 40 30 20 10 0 -10
f1 (ppm)

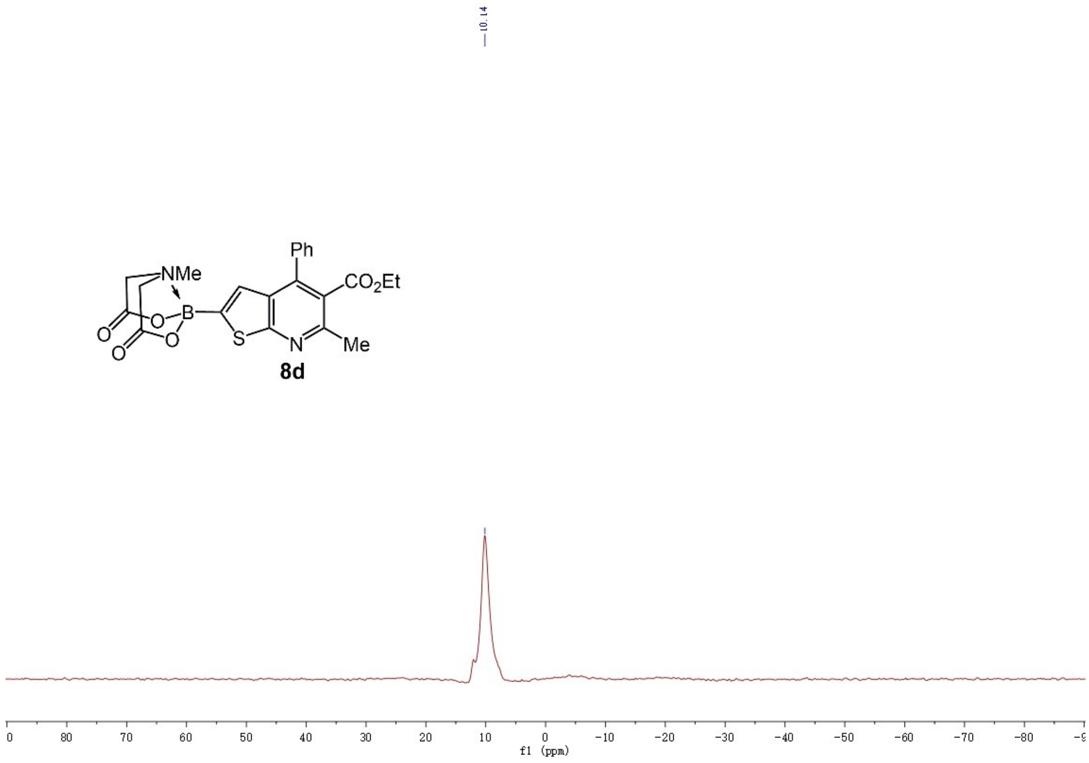
10.14
Ph
NMe
B
O
CO2Et
S
N
Me
8d
80 70 60 50 40 30 20 10 0 -10 -20 -30 -40 -50 -60 -70 -80
f1 (ppm)

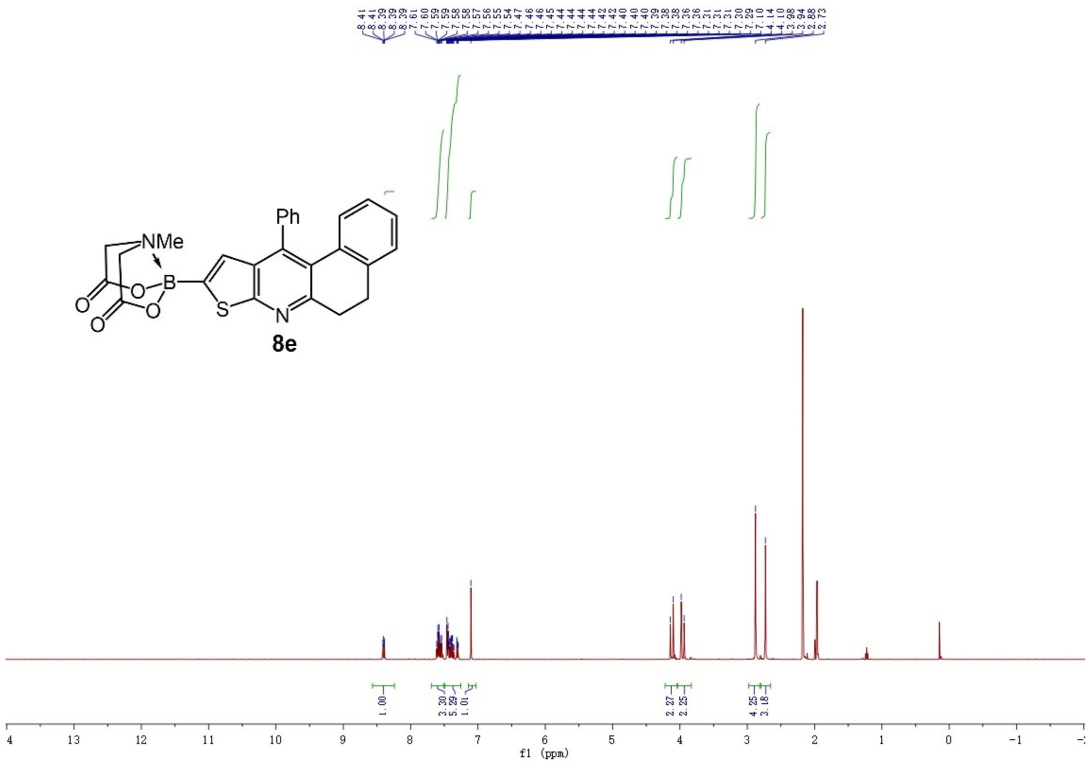

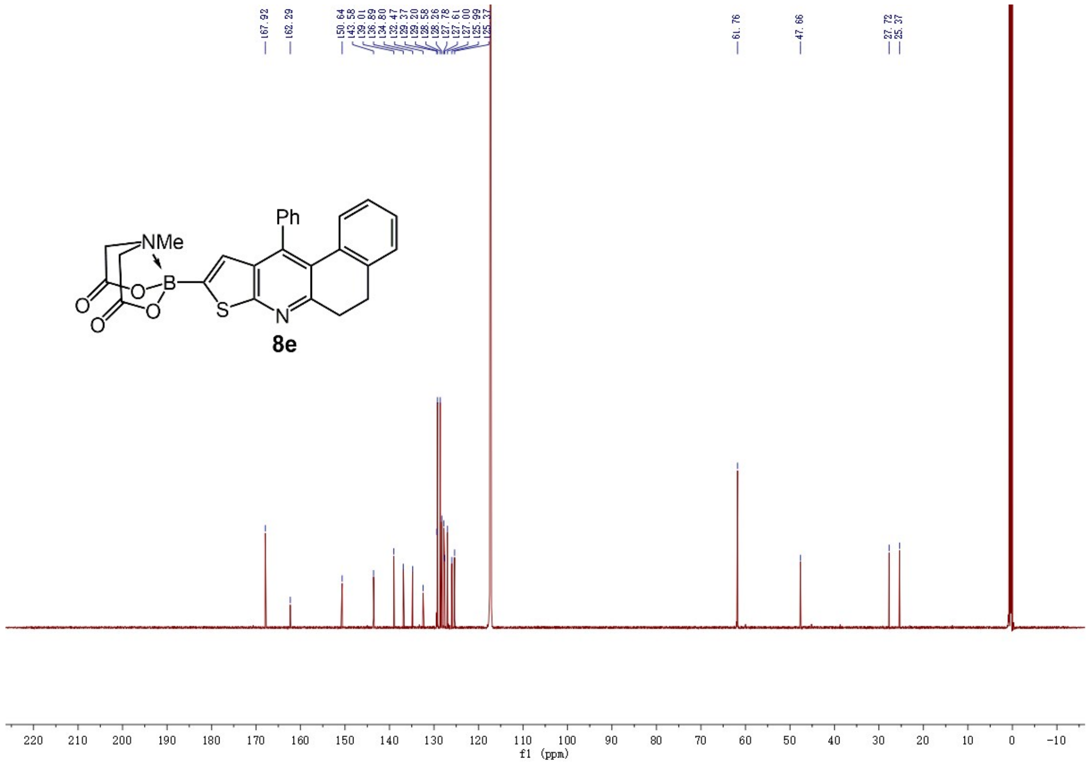

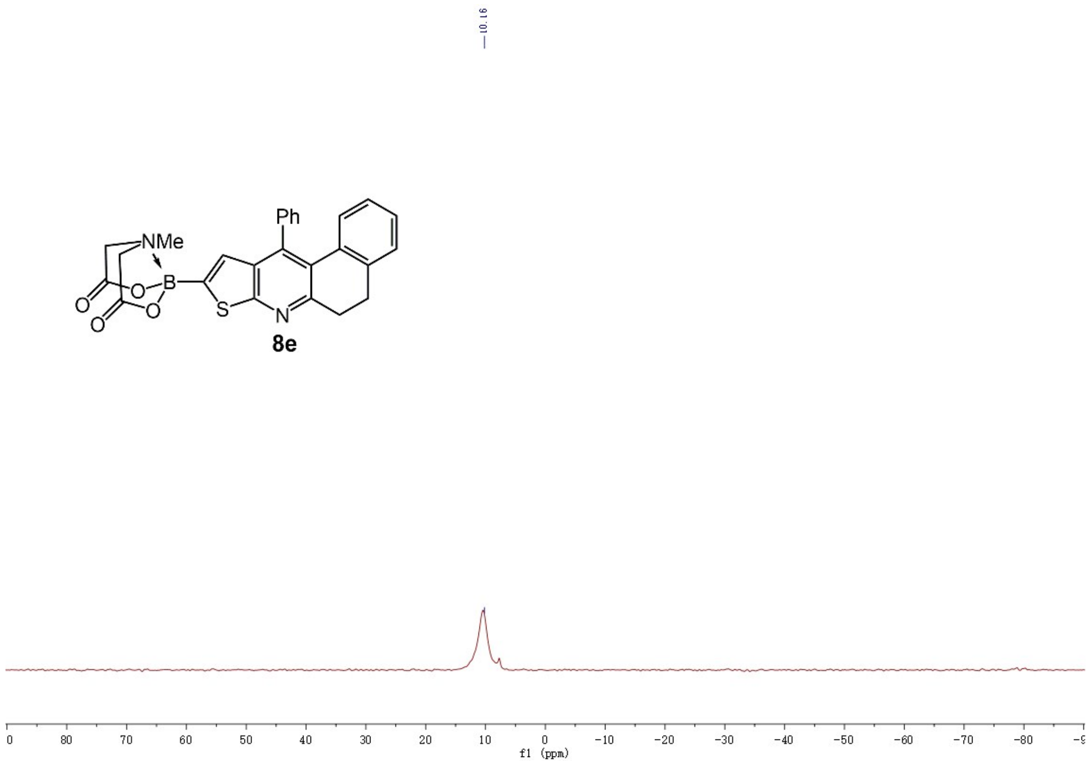

References

1. J. D. St Denis, A. Zajdlik, J. Tan, P. Trinchera, C. F. Lee, Z. He, S. Adachi and A. K. Yudin, *J. Am. Chem. Soc.*, 2014, **136**, 17669-17673.

2. L. Aurelio, C. Valant, B. L. Flynn, P. M. Sexton, J. M. White, A. Christopoulos and P. J. Scammells, *J. Med. Chem.*, 2010, **53**, 6550-6559.
